# EGFR-dependent delivery of phosphorylated IGFBP3 by cancer stem cells drives chemotherapy resistance in colorectal cancer

**DOI:** 10.1038/s41419-026-09009-0

**Published:** 2026-06-29

**Authors:** Xinyu Gao, Lian Zhang, Haibo Wang, Weiguo Zhang, Xiuhong Lu, Jingyi Wang, Liu Liu, You Zhang, Xinshuang Wang, Mengting Song, Wei Xie, Beibei Liang, Xiewei Kang, Jiaxin Zhang, Gang Huang, Hao Yang

**Affiliations:** 1 Shanghai Key Laboratory of Molecular Imaging, Pudong Gongli Hospital, Shanghai University of Medicine and Health Sciences, Shanghai, 201318 China; 2 Department of Radiology, Shanghai First Maternity and Infant Hospital, School of Medicine, Tongji University, Shanghai, 200092 China; 3 Department of Nanomedicine, Houston Methodist Research Institute, Houston, TX 77030 USA; 4 Department of Nuclear Medicine, Shanghai Chest Hospital, Shanghai Jiao Tong University School of Medicine, Shanghai, 200025 China; 5 Department of Nuclear Medicine, Institute of Clinical Nuclear Medicine, Renji Hospital, School of Medicine, Shanghai Jiao Tong University, Shanghai, 200127 China; 6 Key Laboratory of Structure-Based Drug Design & Discovery of Ministry of Education, Shenyang Pharmaceutical University, Shenyang, 110016 China

**Keywords:** Cancer therapeutic resistance, Phosphorylation

## Abstract

The primary systemic treatment for advanced colorectal cancer (CRC) is chemotherapy, but inevitable drug resistance leads to poor prognosis. Cancer stem cells (CSCs) are recognized as significant contributors to chemotherapy resistance, yet the mechanisms underlying their ability to induce widespread resistance across tumor cell populations remain poorly understood. In this study, we demonstrate a novel mechanism by which colorectal CSC-like spheroid cells (CCSCs) drive chemotherapy resistance through the release of small extracellular vesicles (sEVs) enriched with highly phosphorylated insulin-like growth factor binding protein-3 (IGFBP3) into the tumor microenvironment (TME). While cell-free IGFBP3 or endogenous IGFBP3 directly induces apoptosis in tumor cells, IGFBP3 originating from quiescent CCSCs functions through an epidermal growth factor receptor (EGFR)-dependent pathway to promote chemotherapy resistance. Mechanistically, the protein kinase VRK2, which is highly enriched in CCSCs, leads to the phosphorylation of IGFBP3 at the S201 site. This phosphorylation enables IGFBP3 to bind to EGFR, facilitating its sorting and subsequent recruitment into sEVs. Both EGFR and phosphorylated IGFBP3 are co-delivered via sEVs to recipient tumor cells, where they inhibit chemotherapy-induced DNA damage and promote resistance. Importantly, siRNA targeting VRK2 or the inhibitor D4476 significantly reduced IGFBP3 phosphorylation and its incorporation into sEVs, thereby reversing chemotherapy resistance. These findings highlight the dual role of IGFBP3 in CRC and provide critical insights into the core mechanism by which CCSCs drive chemotherapy resistance through EGFR and sEVs-mediated signaling, offering a novel therapeutic approach for personalized treatment of CRC.

## Introduction

According to the latest GLOBOCAN 2022 data, colorectal cancer (CRC) ranks as the third most commonly diagnosed cancer globally and is the second leading cause of cancer-related mortality [1]. Although chemotherapy has been the cornerstone of CRC treatment for decades, metastatic progression and disease recurrence remain the primary contributors to mortality in advanced stages, with acquired resistance further compromising treatment efficacy and patient survival [2]. Previous studies have highlighted the potential role of cancer stem cells (CSCs) as the key drivers behind tumor metastasis, recurrence, and therapeutic resistance [3, 4].

CSC-like cells constitute a distinct subpopulation within tumors, characterized by a slow cell cycle, tumor-initiating potential, and self-renewal capacity [5, 6]. The tumor-initiating ability of CSC-like cells refers to their capacity to initiate new tumor formation at metastatic sites or to overcome microenvironmental challenges. Self-renewal, a defining feature of CSC-like cells, provides long-term proliferative potential, thereby driving cancer progression, metastasis, and resistance to therapy [7]. Current evidence suggests that tumor stemness represents a plastic cellular state, wherein non-CSCs can acquire stem cell-like properties, thereby contributing to tumor progression and therapeutic resistance [8]. Additionally, CSC-like cells exhibit a quiescent state, which has been shown to facilitate their evasion of chemotherapeutic agents [9]. Interactions between tumor cells and immune cells or mesenchymal stromal cells within the tumor microenvironment (TME) play a crucial role by providing growth factors and paracrine signals, including soluble factors or extracellular vesicles, which support the maintenance of CSC-like cells' stemness and quiescence [5, 10].

Small extracellular vesicles (sEVs), also referred to as exosomes, were first identified in 1983 in sheep reticulocytes [11]. These vesicles represent a distinct subclass of extracellular vesicles, secreted by eukaryotic cells, with diameters ranging from 40 to 160 nm. sEVs are composed of a wide range of molecular components, including proteins, nucleic acids, lipids, and other functional biomolecules [12]. Recent studies have highlighted the role of sEVs in mediating intercellular communication by transferring bioactive cargo to both proximal and distal recipient cells through various internalization mechanisms, such as endocytosis, macropinocytosis, phagocytosis, and lipid raft-mediated uptake [13]. It has been demonstrated that the function of CSC-like cells can be influenced through the secretion of sEVs. Specifically, CSC-like cells-derived sEVs have been shown to restore the pluripotent differentiation potential of differentiated tumor cells and reactivate the self-renewal and quiescent properties of CSC-like cells by transferring key molecules, including stem cell-associated transcription factors and survival-promoting miRNAs [14, 15].

Our group previously identified that isocitrate dehydrogenase 1 (IDH1) modulated CRC sensitivity to 5-fluorouracil (5-FU) treatment through acetylation/deacetylation modifications and sEV-mediated transfer [16, 17]. However, whether colorectal CSC-like spheroid cells (CCSCs), as key cell types responsible for malignant properties, express aberrantly modified proteins or secrete sEVs that contribute to drug resistance remains to be explored. Our phosphoproteomic analysis of sEVs derived from CCSCs revealed the presence of insulin-like growth factor binding protein 3 (IGFBP3) and a high level of phosphorylation at the serine 201 (S201) site. IGFBP3, which can function both as a secretory protein and an intracellular protein, exhibits functional heterogeneity within tumors [18]. However, the dual regulatory role of IGFBP3 as both a tumor promoter and suppressor remains poorly understood. Elevated circulating levels of secretory IGFBP3 are positively correlated with CRC risk [19, 20], while the roles of intracellular IGFBP3 and IGFBP3 in sEVs remain unclear. The aim of this study is to elucidate the functional roles of IGFBP3 and its phosphorylation modifications in CRC growth and drug resistance, particularly focusing on the regulatory mechanisms mediated by sEVs derived from CCSCs. We identified EGFR as a functional switch for IGFBP3, demonstrating that EGFR-dependent phosphorylation of IGFBP3 is a critical driver of CRC progression and therapeutic resistance.

## Materials and methods

### Cell lines, plasmids, and reagents

Human Embryonic Kidney Cell Line 293T (ATCC, CRL-3216), colorectal cancer cell line RKO (ATCC, CRL-2577) and SW480 (ATCC, CCL-228) were cultured in high-glucose Dulbecco’s modified Eagle’s medium (DMEM, Gibco, NY, USA) supplemented with 10% fetal bovine serum (LAISI Biotechnology, Shanghai, China) and 1% penicillin-streptomycin (Gibco, MA, USA) at 37 °C under 5% CO_2_. All cell lines were authenticated by short tandem repeats (STR) profiling, and mycoplasma was tested every 2 weeks. For the sorting of CD44-positive and CD44-negative cells, RKO cells were incubated with 5 μL of CoraLitePlus 488-conjugated CD44 antibody (#CL488-65608, Proteintech, Wuhan, China) in 100 μL of cell suspension for 30 min, followed by cell sorting using a flow cytometer (BD FACSMelody, USA). The IGFBP3 knockout (KO) cell line was constructed using the Quick-KO CRISPR-Cas9 kit (Pregen, Shanghai, China). The sequences of multi-gRNAs for IGFBP3-KO include: GCCAGTGCACGCGCGTCGCACGG, TAGCGTTGACGCAGAGCCCGCGG, CTGACGTGCGCACTGAGCGAGG. The encoding genes of IGFBP3 wild-type (WT), non-phosphorylated S201A mutant, and phosphorylated mimic S201D were cloned into the pLV-C-GFPSpark vector (SinoBiological, Beijing, China), and then packaged into lentivirus for stable cell lines establishment. The encoding genes of protein kinases CK1E, VRK3, CK1G1, VRK2, CK1D, and CK1G2 were cloned into the pcDNA4/TO-HA vector and transfected into RKO cells, which were cultured in the presence of 1 μg/mL tetracycline for protein expression. Flag-tagged EGFR plasmid pCMV3-Flag-EGFR were purchased from SinoBiological (cat no. HG10001-NF). All constructs were sequence verified by Sanger sequencing. Oxaliplatin, 5-FU and D4476 were purchased from Selleck Chemical (Shanghai, China).

### CCSC enrichment

CCSCs were isolated from tumors of RKO and SW480 cells extracted from tumor-bearing mice. The tumors were washed with HBSS buffer (Adamas, Shanghai, China) and digested in 100 mg/mL collagenase type I (Yeasen Biotechnology, Shanghai, China), 3 mM CaCl_2_ (Titan, Shanghai, China) and 10 mg/mL hyaluronidase (Yeasen Biotechnology) at 37 °C for 4 h. After digestion, DMEM/F12 medium was added to terminate the digestion, and then the cells were filtered through a 40-μm cell membrane (Corning, NY, USA). The single-cell suspension was spread into low adsorption plates and cultured in serum-free DMEM/F12 medium with 2% B27 supplement (Yeasen Biotechnology), 1% N2 supplement (Yeasen Biotechnology), 20 ng/mL EGF (R&D SYSTEMS, MN, USA), and 10 ng/mL bFGF (PeproTech, NJ, USA).

### CCSC identification

Sphere formation, flow cytometry, cell cycle assay, and cellular membrane PKH67 labeling were used for CCSC identification. The cells were distributed into low-adhesion plates at a density of 2 × 10^4^ cells/mL, and cultured in DMEM/F12 medium with 2% B27, 1% N2, 20 ng/mL EGF, and 10 ng/mL bFGF for 2 weeks. The cells were observed to transform into suspended spheres that formed clusters.

Flow cytometry was used to quantify cell populations with CD44+, CD166+, and CD24+. RKO, SW480, or CCSC cells were incubated with 2 μL PerCP or Vio Bright FITC conjugated-antibody (Miltenyi Biotec, Cologne, Germany) in 100 μL cell suspension at 4 °C for 20 min. The samples were analyzed using a flow cytometer (Novocyte, Agilent Technologies, CA, USA), selecting fluorescence channels based on the antibody specifications. Flow data were processed with NovoExpress (version 1.5.0) or FlowJo (version 10.8.1) software.

For the cell cycle assay, cells were fixed in pre-cooled 70% ethanol at 4 °C overnight. About 500 μL PI/RNase staining buffer was added to the cells and incubated for 15 min at room temperature. DNA content was measured using flow cytometry and analyzed using Flowjo software.

Quiescent CCSCs were identified using cellular membrane PKH67 labeling. RKO or CCSC cells were stained with the lipophilic fluorescent dye PKH67 (Merck, Darmstadt, Germany). Briefly, cells were washed and incubated with the dye for 5 min. The staining was subsequently blocked using 1% bovine serum albumin (Beyotime, Shanghai, China), and the cells were cultured in the supplemented medium as previously described. The medium was replaced every 3 days, and cell images were captured using a fluorescence microscope (Leica, Germany) to monitor the dilution of the fluorescent signal.

### Isolation and characterization of sEVs

Cells were cultured in serum-free DMEM or DMEM/F12 for 72 h. The medium was collected using a 0.45-μm filter (MilliporeSigma, Darmstadt, Germany). The suspended CSC spheres were collected by centrifugation of the supernatant. The supernatant filtrate was centrifuged at 120,000×*g* for 90 min at 4 °C using an Optima XPN-100 ultracentrifuge (Beckman Coulter, CA, USA). The pellet was resuspended in PBS and stored at −80 °C. sEVs concentration and size distribution were analyzed using nanoparticle tracking analysis (NTA, ZetaView Particle Metrix, Meerbusch, Germany). sEV morphological size was assessed using transmission electron microscopy (TEM, FEI Tecnai G2 Spirit, OR, USA), and protein markers were identified by Western blot.

### Western blot

For drugs or sEVs-treated cells, proteins were extracted by lysing cells cultured in six-well plates using RIPA buffer (Keygen Biotech, Nanjing, China). Proteins in the lysate were separated by electrophoresis via SDS-PAGE (Epizyme, Shanghai, China). The proteins were then transferred to a PVDF membrane (Millipore, MA, USA) and blocked in a buffer solution containing 5% milk for 2 h. The membrane was incubated overnight at 4 °C with primary antibodies: EpCAM (#93790), TSG101 (#72312), and IGFBP3 (#64143S) from Cell signaling Technogy (MA, USA); CD44 (A1351), CD24 (A2207), CD166 (A9727), CD133 (A0818), CD63 (A5271), and β-Actin (AC026) from ABclonal (Wuhan, China); GRP94 (R381784), EGFR (250142), phospho-EGFR (R24173), DNA-PKcs (R381174), phospho-DNA-PKcs (310043), H2A.X (R24564), and γH2A.X (381558) from ZENBIO (Chendu, China); GFP (AF1483) from Beyotime (Shanghai, China), and VRK2 (sc-365199) from Santa cruz biotechnology (TX, USA). In particular, the anti-phospho-IGFBP3 S201antibody (anti-pS201) was obtained by immunoreacting a synthetic phosphorylated peptide (c-TDTQNFS(p)SESKRE) in a rabbit by Absin Bioscience (Shanghai, China). The membranes were washed with TBST to remove nonspecific bindings, then incubated with HRP-labeled secondary antibody (Abclonal) for 1 h. Acquisition of images using a chemiluminescence imager (Tanon 5200 multi, Shanghai, China).

### Cellular uptake of sEVs

For in vitro uptake analysis, sEVs were labeled with PKH67 dye following the manufacturer’s instructions as described above. Labeled sEVs were centrifuged to remove excess dye, resuspended in PBS, and added to CRC cells cocultured under 10^9^ particles/mL concentration. After 24 h of incubation, cells were fixed and stained with DAPI (Beyotime, Shanghai, China) to visualize the nucleus and phalloidin (Yeasen Biotechnology) to stain the cytoskeleton. Cell imaging was performed using a confocal microscope (TCS-SP8, Leica, Heidelberg, Germany).

### Cell survival assay under drug stress

Cell viability in response to or 5-FU was assessed using the Cell Counting Kit-8 (Yeasen Biotechnology). Cells were seeded in 96-well plates at 8000 cells/well and exposed to various drug concentrations for 48 h. Optical density at 450 nm was measured, and analyze the data calculation.

### Cell apoptosis assay

Apoptosis was detected using Annexin V-FITC/PI or Annexin V-PerCP/PI apoptosis kit (Yeasen Biotechnology). SW480 or RKO cells were spread into 12-well plates at 4 × 10^5^ cells with 5-FU and Oxaliplatin treatment according to different experimental conditions for 48 h. The cell precipitates were washed with PBS, and then 100 μL of 1× binding buffer was used to resuspend the precipitates. 5 μL of Annexin V and 5 μL of PI staining solution were added and incubated with cells. The Annexin V or PI positive cells were analyzed using a flow cytometer (Novocyte), and data were processed using NovoExpress software.

### Tandem mass tags phosphoproteomics analysis

sEVs collected from supernatant isolation were subjected to proteomic analysis using LC-MS/MS at Shanghai Luming BioTech. Briefly, the phosphorylated peptides of sEVs proteins were enriched and labeled with tandem mass tags (TMT) reagents. The chromatography conditions involved loading the samples at a flow rate of 300 nL/min onto a pre-column (Acclaim PepMap100 100 µm × 2 cm, RP-C18, Thermo Fisher, USA) followed by separation on an analytical column (Acclaim PepMap RSLC, 75 µm × 15 cm, RP-C18, Thermo Fisher). Subsequent mass spectrometric analysis was conducted on a Fusion mass spectrometer (Thermo Fisher) equipped with an Easyspray source (Thermo Fisher). Phosphorylated peptides and phosphorylation sites were identified by comparing the mass spectrometry data with the UniProt protein database using MaxQuant software. Principal component analysis (PCA) was performed to assess inter-sample differences using R software. Motif analysis, based on a data-driven mathematical-statistical model, was employed to identify protein kinase recognition patterns for specific residues, indicating kinase preferences for substrates. Differential phosphorylation site motifs were analyzed using MOMO software [21].

### Immunoprecipitation

Cell lysates were prepared using RIPA buffer, and the supernatant was subsequently mixed with the specific antibody or IgG control. The mixture was incubated overnight at 4 °C to allow for the binding of the antibody to the target protein. The antibody-protein complex was then added to Protein A/G magnetic beads (Share-bio, Shanghai, China), and the incubation continued overnight at 4 °C. Afterward, the beads were washed, and loading buffer was added to elute the protein complexes. Finally, the expression of the target protein was assessed by Western blot.

### Multiplex immunofluorescence

Multicolor immunofluorescence staining was performed using the Tyramide Signal Amplification (TSA) assay kit (Abclonal, Wuhan, China) according to the manufacturer’s protocol. Cell samples were fixed with 4% paraformaldehyde (Share-bio, Shanghai, China) for 20 min and subsequently permeabilized with 0.1% Triton X-100 (Adamas-beta) for 20 minutes. Following permeabilization, the samples were treated with 3% hydrogen peroxide (Sangon Biotech). The samples were then blocked with 3% BSA for 30 min. Diluted primary antibodies were added to the samples and incubated overnight at 4 °C. After removing the primary antibody, a HRP-conjugated secondary antibody from the same species as the primary antibody was applied. Tyramide fluorescent dye was then introduced, and the reaction was allowed to proceed for 10 min. This process was repeated for the second and third rounds of labeling with additional tyramide fluorescent dyes. The nuclei were counterstained with DAPI. The samples were ready for observation and imaging using a confocal microscope.

### Molecular docking analysis

The three-dimensional structure of the small-molecule compound D4476 (PubChem CID: 6419753) was obtained from the PubChem database. The predicted structure of the IGFBP3 protein (Accession: AF-P17936-F1-v4) was retrieved from the AlphaFold Protein Structure Database [22]. Crystal structures of VRK2 (PDB ID: 8Q1Z) and EGFR (PDB ID: 8SC7) proteins were acquired from the Protein Data Bank (PDB). Prior to docking, all protein and ligand structures were preprocessed using AutoDock 4.6.2. This included the addition of polar hydrogen atoms and the removal of crystallographic water molecules to optimize docking conditions. To assess potential interactions between D4476 and the VRK2 protein, molecular docking simulations were performed using AutoDock. The docking results were visualized using PyMOL version 2.6.0. Furthermore, to evaluate the potential protein–protein interaction between IGFBP3 and EGFR, docking was conducted using the HDOCK server with default parameters. The resultant complexes were similarly visualized using PyMOL version 2.6.0.

### Comet assay

DNA damage was assessed using a commercial Comet assay kit (Beyotime) according to the manufacturer’s protocol. Cells were embedded in agarose on slides, lysed with pre-cooled lysis solution at 4 °C for 2 h, and subjected to electrophoresis under alkaline conditions. Following electrophoresis, slides were stained with propidium iodide, washed, and visualized using confocal microscopy. DNA damage was indicated by the presence of comet-like tails, and the extent of damage was quantitatively analyzed using Image J software based on tail length and DNA content.

### RNA silencing

CCSCs or RKO cells were transfected with siRNA using Lipofectamine 3000 transfection reagent (Thermo Fisher) according to the manufacturer’s protocol. The sequences for the VRK2-targeting siRNA and the control siRNA are as follows: siVRK2-1: UGGAUGUACUGGAAUAUAUTT(sense)/AUAUAUUCCAGUACAUCCAAC(anti-sense); siVRK2-2: CAAACUAUCAAGCCCUCAATT(sense)/ UUGAGGGCUUGAUAGUUUGGC(anti-sense); siNC; UUCUCCGAACGUGUCACGUTT(sense)/ACGUGACACGUUCGGAGAATT(anti-sense).

### Protein kinase prediction

Kinase prediction was performed using the online resource NetPhos 3.1 [23]. The IGFBP3 protein sequence in FASTA format was input, followed by neural network-based integration to predict serine phosphorylation sites within IGFBP3 in eukaryotic proteins. Kinases with scores greater than 0.5 were considered significant. Additionally, based on the protein–protein interaction (PPI) network, the iGPS 1.0 software was utilized to predict the protein kinases that interact with the phosphorylation site S201 of IGFBP3 in eukaryotes [24].

### Xenograft tumor and imaging

All animal experiments were performed in accordance with the guidelines provided by the Animal Ethics Committee of the Shanghai University of Medicine and Health Sciences. Lentivirus with luciferase (GenePharma, Shanghai, China) was infected with RKO to obtain RKO⁃luc cells. The luciferase-expressing RKO-luc cells were cultured in large quantities, and the logarithmic growth phase cells were collected. About 8 × 10^6^/mL RKO⁃luc cells were slowly injected into the axilla of 20 Balb/c mice (Jihui Laboratory Animal Care, Shanghai, China) with 200 μL of cell suspension subcutaneously, and the tumor volume was recorded once every 3 days. When the tumor volume reached 200 mm^3^, it was randomly divided into four groups for administration of treatment every 3 days: solvent of equal volume, 10 mg/kg 5-FU, 20 mg/kg D4476, 10 mg/kg 5-FU + 20 mg/kg D4476. For bioluminescence imaging, Balb/c mice were injected intraperitoneally with 10 mL/kg of D⁃Luciferin (Yeasen Biotechnology) and imaged using an IVIS Spectrum (PerkinElmer, USA). For micro- PET/CT, mice were food-restricted for 8 h before PET/CT imaging (Siemens Medical, Germany) to ensure optimal glucose uptake. Each mouse was injected with 200 μCi ^18^F⁃FDG imaging agent through the tail vein and imaged. After imaging, HE staining and immunohistochemistry was performed to assess protein expression in ex vivo tumor tissues.

### Immunohistochemical analysis

Tumor tissues were fixed in 4% paraformaldehyde for 3 h, and part of the tissue was embedded in paraffin. The 2–3-μm slices were subjected to blocking, deparaffinization, and rehydration, followed by incubation in a high-temperature antigen retrieval solution (Beyotime) for 20 min. After cooling, the slices were treated with 3% H_2_O_2_ for 10 min, and then blocked with 3% BSA at room temperature for 1 h. After blocking, the slices were incubated with the primary antibody, washed three times with PBST, and then incubated with the secondary antibody. Color development was carried out with 1×DAB (Beyotime), and the slices were observed and photographed under a microscope.

### Clinical data preparation and analysis

Colorectal cancer tissue microarrays (containing paired cancer and adjacent normal tissues from 48 patients) were obtained from Shanghai Weiaobio, followed by immunohistochemical analysis. All the participants have signed the INFORMED CONSENT form. The Omicshare online analysis tool (Guangzhou Gene Denovo) was used to analyze the RNA-seq expression differences of IGFBP3 between cancer and adjacent tissues across 21 cancer types in the TCGA database. The GEPIA online database (gepia.cancer-pku.cn) was used to analyze RNA expression differences of IGFBP3, EGFR, and VRK2 in cancer or adjacent tissues of COAD and READ. All experiments were performed in accordance with the guidelines provided by the Ethics Committee of the Shanghai University of Medicine and Health Sciences.

### Statistical analysis

The experimental data were statistically analyzed and plotted as bar charts using SPSS 25.0 and GraphPad Prism 9.5 software, and all results between groups were expressed as the mean ± standard deviation of at least three replicates. Comparisons between the two groups were made using *t*-tests, and one-way analysis of variance (ANOVA) was used for multiple comparisons between groups. Spearman’s coefficient was used to determine the correlation between two variables. *P* < 0.05 (∗) is being taken as a statistically significant difference.

## Results

### CCSC secretion of sEVs promotes chemoresistance in CRC

**Fig. 1:**
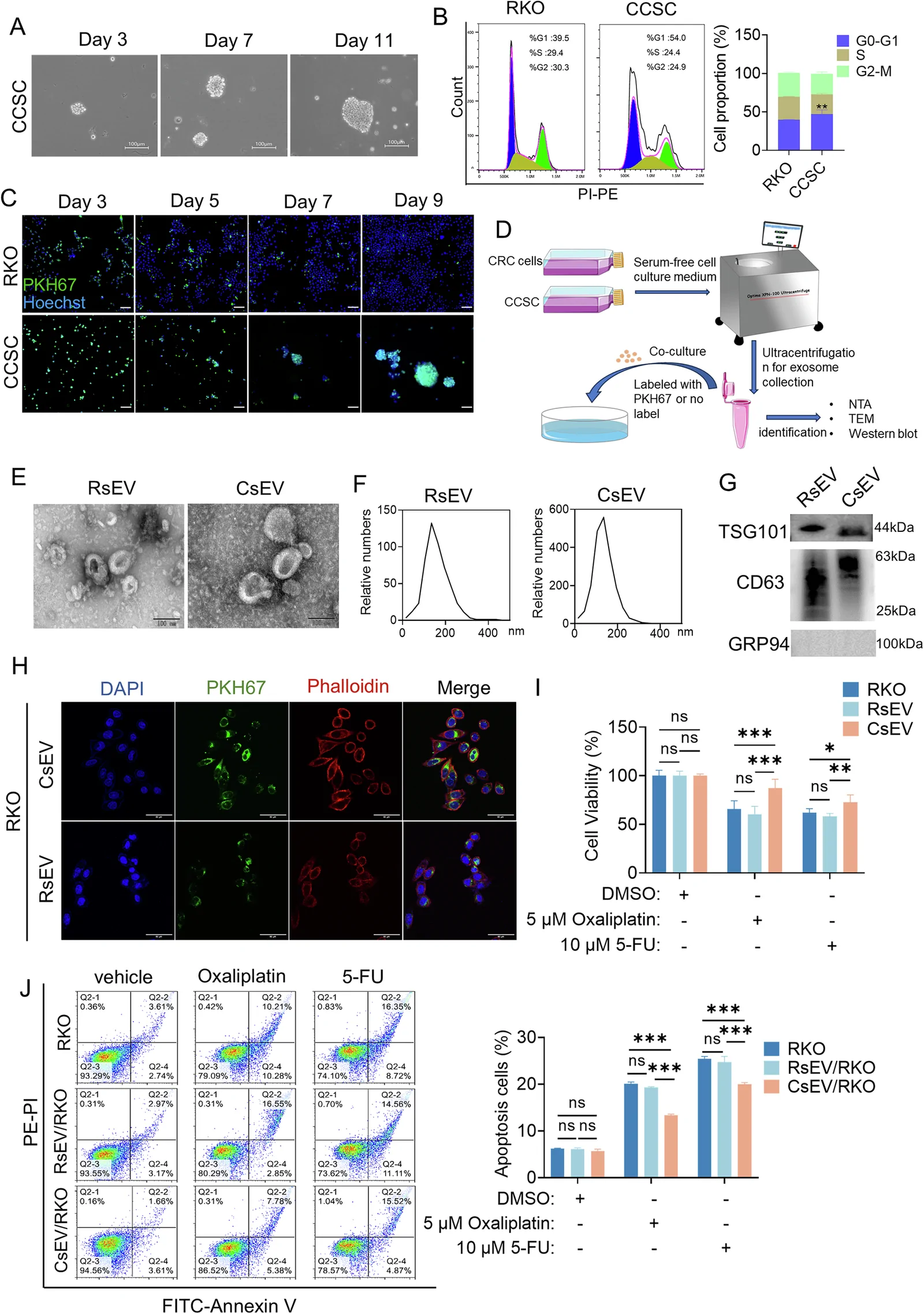
Identification of CCSCs-derived sEVs and their role in promoting chemotherapy resistance. **A** RKO-derived CCSCs were enriched in a low-adhesion plate using stem cell culture medium supplemented with growth factors, and sphere formation was observed. Scale bar: 100 μm. **B** Cell cycle analysis of CCSCs and RKO cells, with the proportions of each phase calculated. **C** Identification of quiescent CCSCs, visualized by PKH67 and Hoechst 33342 staining under fluorescence microscopy. Scale bar: 50 μm. **D** Schematic showing the isolation of sEVs from different cell populations by ultracentrifugation, followed by functional characterization after co-culturing the sEVs with RKO cells. **E** Transmission electron microscopy images of RKO and CCSC-derived sEVs (RsEV and CsEV) morphology. Scale bar: 100 nm. **F** Nanoparticle tracking analysis of the sEVs particle size distribution. **G** Western blot analysis of sEVs-positive markers TSG101 and CD63, and negative marker expression. **H** Confocal microscopy images of sEVs uptake, with sEVs stained with PKH67 (green), cell nuclei stained with DAPI (blue), and cytoskeleton stained with phalloidin (red). Scale bar: 50 μm. **I** RKO cells were cocultured with RsEV or CsEV at a concentration of 10^9^ particles/mL and treated with 5 μM oxaliplatin or 10 μM 5-FU for 48 h. Cell viability was measured using a CCK-8 assay. **J** Cell apoptosis was measured using Annexin V-FITC/PI dual staining. Data were presented as mean ± SD (*n* = 3; ns not significant, *P < 0.05, **P < 0.01, and ***P < 0.001).

We isolated colorectal CSC-like spheroid cells (CCSCs), including SW480 and RKO cells (Supplementary Fig. 1A). Briefly, subcutaneous tumors derived from RKO or SW480 cells in mice were digested into single-cell suspensions using collagenase, followed by three-dimensional suspension culture in low-attachment plates with cytokine-enriched medium to promote CCSC enrichment. These CCSCs demonstrated the ability to form spheres (Fig. 1A and Supplementary Fig. 1B). Importantly, their cell cycle was predominantly residing in the G0/G1 phase (Fig. 1B and Supplementary Fig. 1C). While the cell membrane PKH67 fluorescence signal in two-dimensional cultured RKO cells was diluted in accordance with their higher proliferative activity, CCSCs retained the original PKH67 fluorescence over the same experimental period (Fig. 1C). This suggests cells within three-dimensional CCSCs spheroids exhibit a reduced proliferation rate. Furthermore, the expression levels of self-renewal markers, including CD24, CD44, and CD166, were significantly higher in the CCSCs compared to the initial RKO cells (Supplementary Fig. 1D, E). Given our previous findings that small extracellular vesicles (sEVs) secreted by quiescent CSC have potential chemoresistance effects [25, 26], we isolated sEVs from RKO cells (RsEVs), SW480 cells (SsEVs), and CCSCs (CsEVs). These sEVs were confirmed through electron microscopy (Fig. 1E and Supplementary Fig. 1F), NTA analysis (Fig. 1F and Supplementary Fig. 1G), and the expression of vesicle surface markers (Fig. 1G and Supplementary Fig. 1H). Isolated RsEVs, SsEVs, and CsEVs were labeled with the PKH67 dye. The labeled sEVs were then incubated with RKO and SW480 cells, respectively. Fluorescence microscopy images demonstrated that PKH67-labeled CsEVs were efficiently internalized by RKO cells (Fig. 1H) and SW480 cells (Supplementary Fig. 2A), leading to an increased resistance of parental cells to chemotherapy agents such as oxaliplatin and 5-FU (Fig. 1I, J). Compared to RsEV or SsEVs, the IC50 values of oxaliplatin and 5-FU were significantly increased in RKO and SW480 cells cocultured with CsEVs (Supplementary Fig. 2B–E).

To determine whether the production of CsEVs that attenuate chemotherapeutic efficacy is a feature of spheroids under three-dimensional culture conditions or an intrinsic property of stemness, CD44-positive and CD44-negative RKO cells were sorted based on the stem cell marker CD44 (Supplementary Fig. 2F, G). CD44+ CCSCs were further enriched from CD44 + RKO cells, and their derived sEVs (CD44+ CsEVs) were collected. However, due to the lack of stemness, CD44− CCSCs could not be successfully established in the three-dimensional culture system. Drug cytotoxicity assays demonstrated that, compared with RsEVs derived from CD44− or CD44 + RKO cells, co-culture with CD44+ CsEVs or total CsEVs significantly increased resistance to oxaliplatin and 5-FU, with no significant difference observed between CD44+ CsEVs and CsEVs (Supplementary Fig. 2H). These findings indicate that CCSC spheroids formed under three-dimensional culture conditions possess both stem-like and quiescent characteristics, and that the CsEVs they secrete contribute to enhanced chemoresistance.

### Increased IGFBP3 phosphorylation in CCSC-derived sEVs

**Fig. 2:**
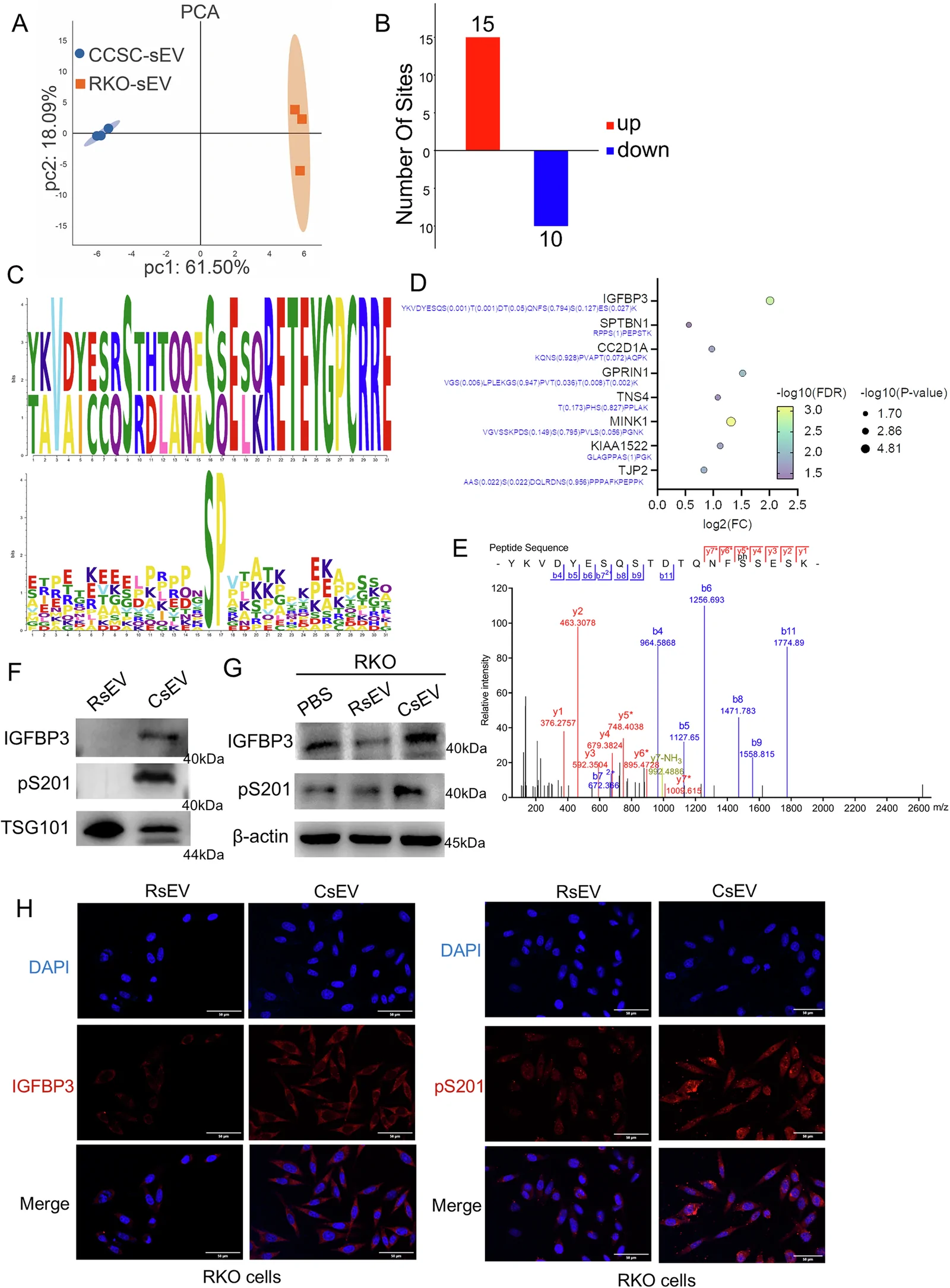
Phosphorylation profiling of sEVs proteins reveals phosphorylation of IGFBP3 in CCSCs. **A** TMT phosphoproteomics analysis indicated a significant difference in the principal component analysis (PCA) of sEVs secreted by CCSCs and RKO cells. **B** Quantification of differentially phosphorylated proteins in sEVs. **C** The two predominant phosphorylation motifs at phosphorylation sites within sEVs derived from CCSCs. **D** Expression analysis of differentially expressed proteins matching both phosphorylation motifs. **E** Secondary mass spectrometry analysis of the phosphopeptide at the S201 site of IGFBP3. **F** Western blot detecting the expression of IGFBP3 and pS201 in sEVs derived from RKO and CCSCs. **G**, **H** After incubation of sEVs secreted by CCSCs and RKO cells in RKO cells for 48 h, levels of IGFBP3 and pS201 were analyzed using Western blot and immunofluorescence.

Post-translational modifications of proteins, such as phosphorylation, play a crucial role in determining the distinct activities of proteins at various stages of cancer progression. To elucidate the mechanism by which CsEVs induce chemoresistance, we identified differentially phosphorylated proteins in CsEVs through mass spectrometry (Fig. 2A). All phosphorylated protein cargos identified in CsEVs and RsEVs are listed in Supplementary Table 1. A total of 25 differentially phosphorylated proteins were detected in CsEVs (Fig. 2B). These differential phosphorylation modifications, primarily occurring on serine residues, predominantly exhibited specific motifs, including “xxxxxxxxxxxxxxx_S_Pxxxxxxxxxxxxxx” or “xxVxxxxxSxxxxxx_S_xExxxxxxxxxxxxx” (Fig. 2C). Among the phosphorylated proteins that align with these motifs, the phosphorylation of serine at position 201 (pS201) in IGFBP3 showed the most significant differential expression between RsEVs and CsEVs (Fig. 2D, E). Western blot analysis revealed that both IGFBP3 and its pS201 modification were significantly upregulated in CsEVs (Fig. 2F and Supplementary Fig. 3A). Co-culturing CsEVs with RKO or SW480 cells also led to an increased expression of IGFBP3 pS201 in the recipient cells (Fig. 2G, H and Supplementary Fig. 3B). Therefore, the specific encapsulation of highly phosphorylated IGFBP3 in CsEVs may play a role in the biological processes underlying CRC treatment resistance.

### sEVs-delivered phosphorylated IGFBP3 promotes chemoresistance via an EGFR-dependent mechanism

**Fig. 3:**
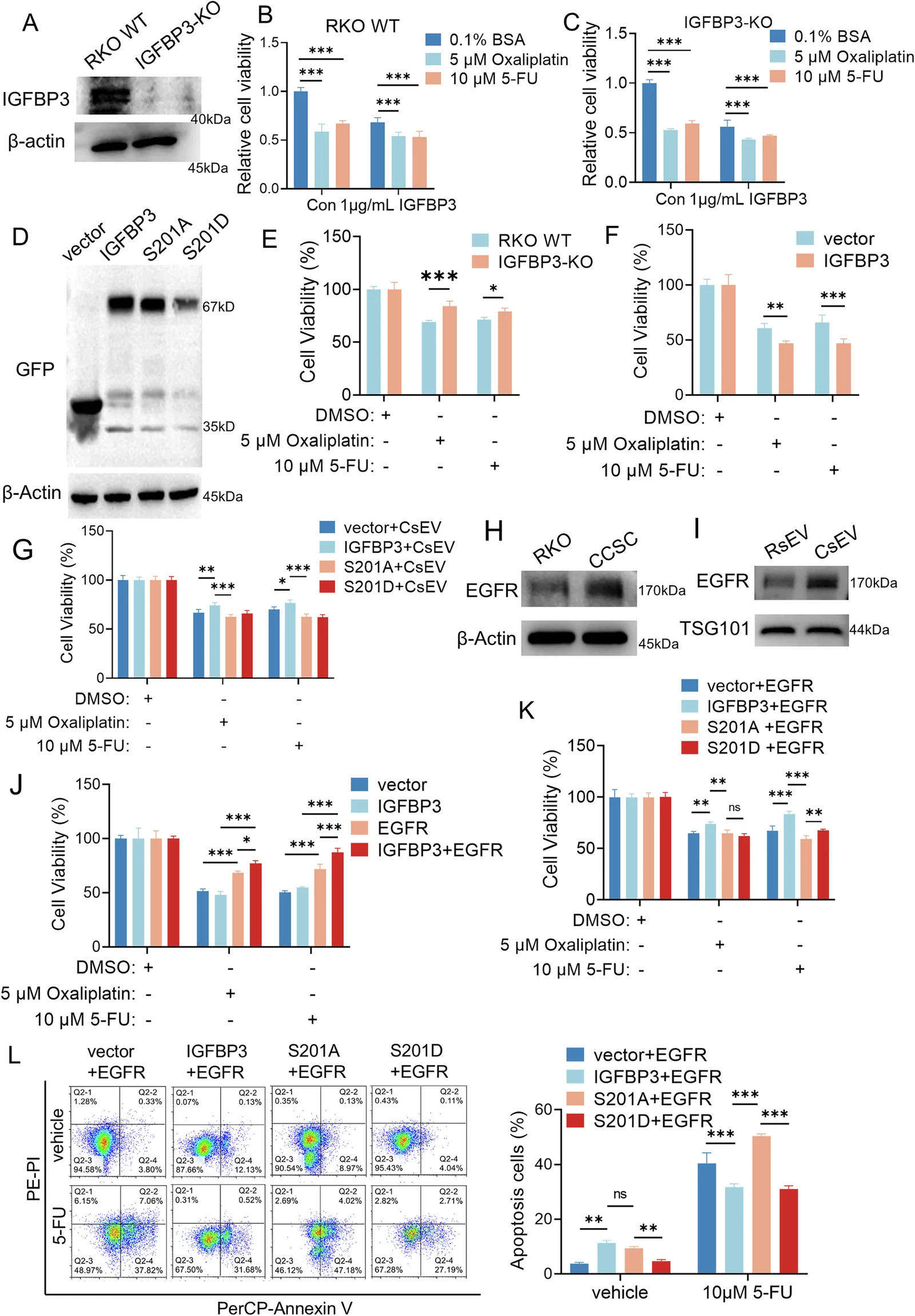
Phosphorylation of IGFBP3 in CCSC-derived sEVs promotes chemoresistance. **A** Construction of the IGFBP3 knockout (IGFBP3-KO) RKO cell line using CRISPR-Cas9. **B**, **C** In serum-free conditions, 1 μg/mL IGFBP3, 5 μM oxaliplatin, and/or 10 μM 5-FU were added to the culture media of RKO and IGFBP3-KO RKO cells for 48 h, followed by CCK-8 assay to assess cell viability. **D** GFP-vector, GFP-tagged IGFBP3, non-phosphorylated mutant S201A, and phosphomimetic mutant S201D were introduced into IGFBP3-KO RKO cells via lentiviral transduction. A Western blot was performed to verify stable expression of these proteins. **E**, **F** RKO, IGFBP3-KO RKO, and IGFBP3-overexpressing RKO cells were treated with 5 μM oxaliplatin or 10 μM 5-FU for 48 h, and cell viability was assessed by CCK-8 assay. **G** 10^9^ particles/mL CsEVs were added to RKO cells expressing IGFBP3, S201A, or S201D, followed by 48-h treatment with 5 μM oxaliplatin or 10 μM 5-FU. Cell viability was measured by the CCK-8 assay. **H**, **I** Western blot analysis of EGFR expression in RKO cells, CCSC cells, and their derived sEVs. **J**, **K** Flag-tagged EGFR plasmid or control plasmid was transfected into the four cell lines. After transfection, the cells were treated with 5 μM oxaliplatin or 10 μM 5-FU for 48 h, and cell survival was assessed using CCK-8. **L** Cell apoptosis was evaluated by Annexin V-PerCP and PI double staining after 48-h treatment with 10 μM 5-FU. Data were presented as mean ± SD (*n* = 3; ns not significant, *P < 0.05, **P < 0.01, and ***P < 0.001).

Given that IGFBP3 may have dual functions in promoting tumor growth and apoptosis, our aim is to explore the precise mechanisms of IGFBP3 in CRC progression and treatment resistance. IGFBP3 can exist as an extracellular secreted protein or an intracellular protein. Extracellular IGFBP3 may directly bind to its cell membrane receptor (IGFBP3R) or target IGFs receptors after binding to IGFs [27]. We found that extracellular IGFBP3 inhibited RKO cell growth and acted as a tumor suppressor, enhancing the therapeutic efficacy of oxaliplatin or 5-FU, regardless of whether the cells are wildtype or IGFBP3 knockout (KO) (Fig. 3A–C). In the presence of IGF1, extracellular IGFBP3 still inhibited RKO cell growth (Supplementary Fig. 4A). For the functional study of intracellular IGFBP3, we stably overexpressed IGFBP3 and its S201 phosphorylation mutant protein in IGFBP3-KO cells (Fig. 3D). The results showed that the absence of intracellular IGFBP3 increased resistance to oxaliplatin or 5-FU (Fig. 3E). Overexpression of intracellular IGFBP3 in RKO cells enhanced their sensitivity to chemotherapy (Fig. 3F and Supplementary Fig. 4B). These results demonstrate that both extracellular secreted IGFBP3 (whether via an IGF1-dependent pathway or not) and intracellular IGFBP3 primarily act as tumor suppressors, inhibiting CRC cell growth and treatment resistance.

In contrast, CsEVs, as a carcinogenic component, encapsulate high levels of IGFBP3 and its phosphorylation, suggesting that IGFBP3 has a different function from previous cognition. RKO cells with high IGFBP3 expression, cocultured with CsEVs, showed increased resistance to chemotherapy compared to wild-type cell lines. In contrast, cells expressing the low-phosphorylation mutant protein IGFBP3 S201A partially restored sensitivity to chemotherapy (Fig. 3G). This indicates that other components carried by CsEVs play a role in determining the function of IGFBP3. Previous studies have shown that IGFBP3 activated EGFR, leading to tumor resistance, and EGFR played a critical role in the formation of CCSCs [28, 29]. Therefore, we hypothesize that CsEVs carry EGFR derived from CCSCs, which, in combination with phosphorylated IGFBP3, synergistically promotes chemotherapy resistance. Both CCSC cells and the CsEVs they secrete contained high levels of EGFR protein (Fig. 3H, I). We overexpressed exogenous EGFR in RKO cells (Supplementary Fig. 4C) and observed that under EGFR overexpression conditions, IGFBP3 enhanced resistance to oxaliplatin or 5-FU (Fig. 3J). In the presence of EGFR, cells expressing the S201A mutant of IGFBP3 showed increased sensitivity to chemotherapy drugs compared to wild-type IGFBP3 (Fig. 3K, L). Compared with S201A, cells expressing the IGFBP3 phosphorylation-mimetic mutant S201D exhibited increased resistance to chemotherapeutic treatment (Fig. 3K, L). Therefore, the high phosphorylation of IGFBP3 in CsEVs, under EGFR-dependent conditions, promotes chemotherapy resistance.

### Phosphorylated IGFBP3 binds to EGFR and alleviates chemotherapy-induced DNA damage

**Fig. 4:**
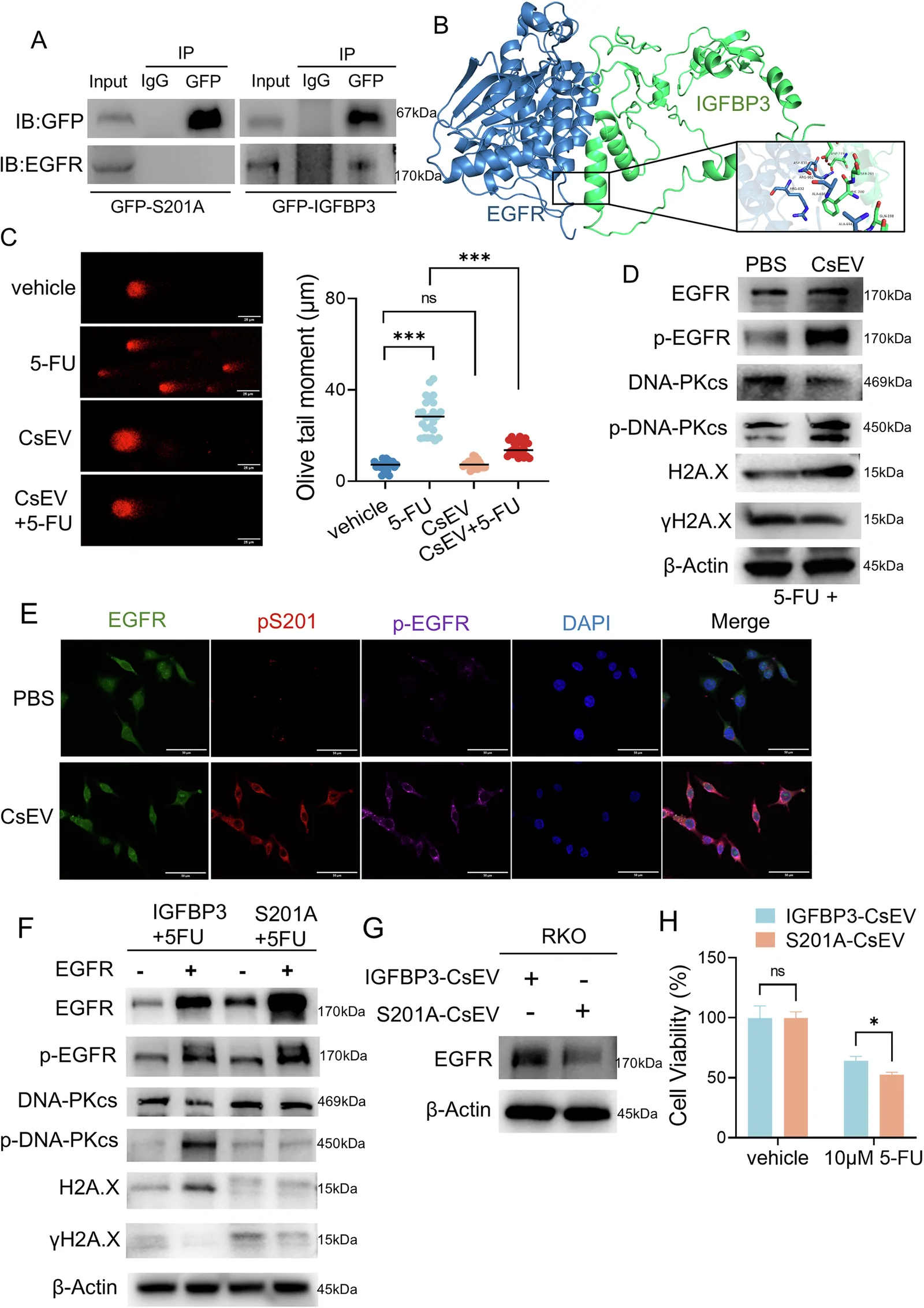
Phosphorylated IGFBP3 in CCSC-derived sEVs promotes DNA damage repair in an EGFR-dependent manner. **A** Lysates from RKO cells stably expressing GFP-IGFBP3 or GFP-S201A were subjected to immunoprecipitation using anti-GFP or control IgG antibodies, followed by Western blot analysis to assess the interaction between IGFBP3 and EGFR. **B** Molecular docking simulation was performed to predict the interaction interface between IGFBP3 and EGFR proteins. **C** A comet assay was employed to evaluate DNA strand breaks in RKO cells following treatment with 10 μM 5-FU, 10⁹ particles/mL CsEVs, or their combination. **D** Western blotting was conducted to analyze the expression of DNA damage and repair-associated proteins in RKO cells treated with 10 μM 5-FU and 10⁹ particles/mL CsEVs for 48 h. **E** Multiplex immunofluorescence staining was used to visualize the co-localization of IGFBP3 pS201 with EGFR and p-EGFR in RKO cells after exposure to 10⁹ particles/mL CsEVs. **F** Flag-tagged EGFR was transfected into RKO cells stably expressing IGFBP3 or the S201A mutant. Cells were then treated with 10 μM 5-FU, and DNA damage/repair-related protein expression was examined by Western blot. **G** sEVs were isolated from CCSCs, which were enriched from RKO cells stably expressing IGFBP3 (IGFBP3-CsEV) or S201A (S201A-CsEV). RKO recipient cells were incubated with 10⁹ particles/mL of these sEVs for 48 h, and EGFR expression was evaluated by Western blot. **H** RKO cells were treated with 10⁹ particles/mL of IGFBP3-CsEV or S201A-CsEV in combination with 10 μM 5-FU for 48 h. Cell viability was assessed using the CCK-8 assay. Data were presented as mean ± SD (*n* = 3; ns not significant, *P < 0.05).

The phosphorylation of IGFBP3 in CsEVs and its co-delivery with EGFR is dependent on their interaction. Co-immunoprecipitation experiments revealed a binding between IGFBP3 and EGFR proteins, with the S201A mutation in IGFBP3 causing a loss of binding affinity for EGFR (Fig. 4A). Molecular docking results from the HDOCK Server demonstrated the interaction between IGFBP3 and EGFR (Fig. 4B). At the docking interface, the S201 and phenylalanine (F) 200 sites of IGFBP3 formed amino acid interactions with the alanine (A) 698 site of EGFR. This suggests that S201 is essential for the binding of IGFBP3 to EGFR.

The reason why phosphorylated IGFBP3 and EGFR carried by CsEVs promote chemotherapy resistance is the enhanced DNA damage repair process. Comet assays demonstrated that CsEVs repaired 5-FU-induced DNA damage (Fig. 4C). Additionally, CsEVs increased the phosphorylation of EGFR (Fig. 4D, E and Supplementary Fig. 5A) and reduced the S139 phosphorylation of H2A.X (γ-H2A.X), a marker of DNA double-strand breaks (DSB), while increasing the phosphorylation modification of the DSB repair component DNA-PKcs (Fig. 4D). Further investigation revealed that, compared to cells expressing IGFBP3 alone, RKO cells co-expressing IGFBP3 and EGFR exhibited stronger damage repair. However, the IGFBP3 S201A mutant protein lost its ability to synergistically promote damage repair with EGFR (Fig. 4F). CsEVs carry high levels of EGFR protein to recipient cells, a process governed and determined by phosphorylated IGFBP3 (Fig. 4G). This is because there was no significant difference in EGFR expression between CCSC cells expressing wild-type IGFBP3 and the S201A mutant (Supplementary Fig. 5B). However, CsEVs expressing phosphorylated IGFBP3 sorted higher levels of EGFR, compared to the S201A mutant (Supplementary Fig. 5C). CsEVs carrying the S201A mutation partially restored 5-FU sensitivity (Fig. 4H) and increased DNA damage repair (as evidenced by reduced phosphorylation of DNA-PKcs and increased γ-H2A.X, Supplementary Fig. 5D). These data suggest that CCSC cells sort phosphorylated IGFBP3 and EGFR into sEVs, enhancing DNA damage repair in recipient cells after chemotherapy-induced DNA damage.

### VRK2 phosphorylates IGFBP3 in CCSCs

**Fig. 5:**
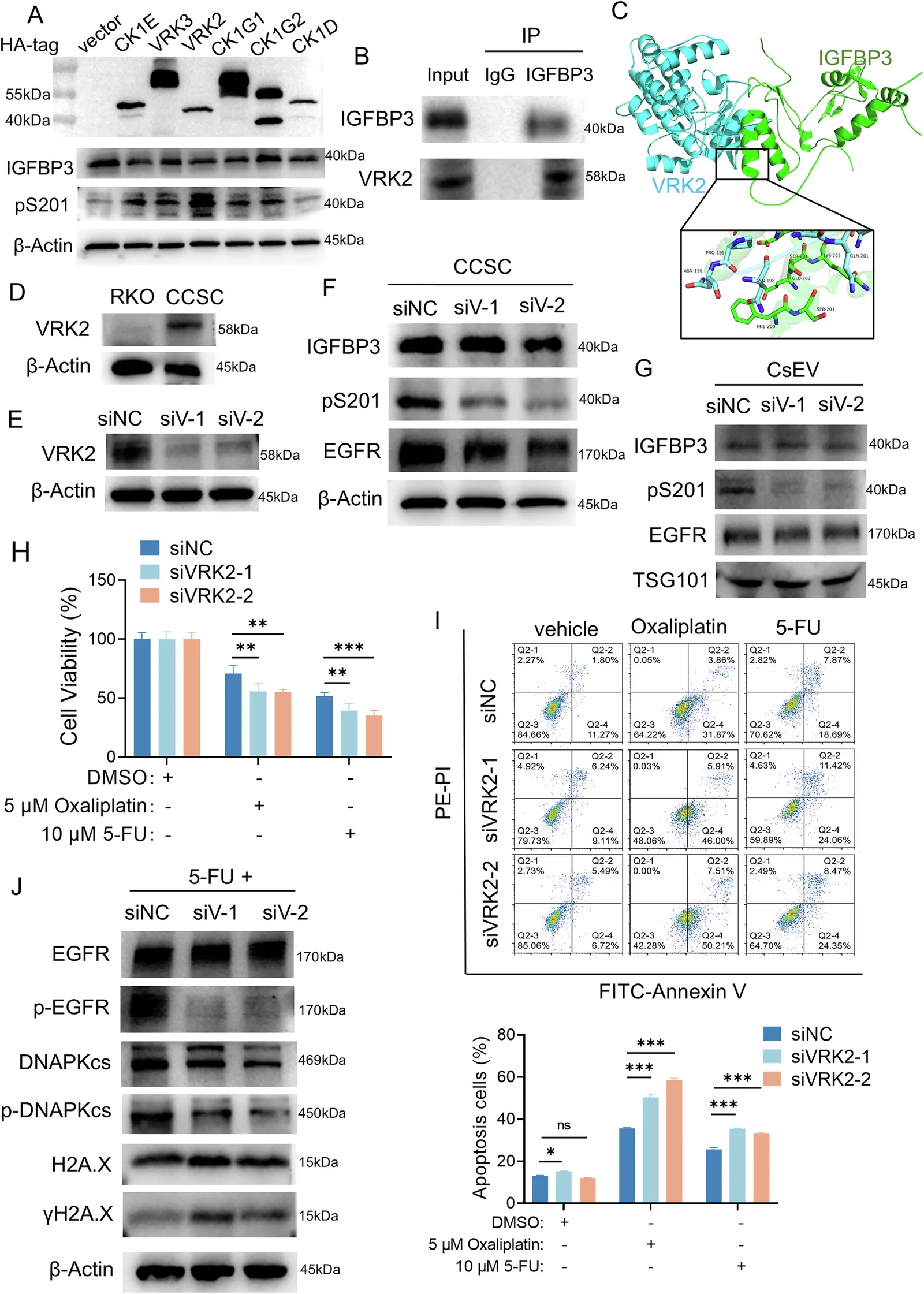
VRK2 phosphorylates IGFBP3 in CCSCs. **A** RKO cells were transfected with HA-tagged constructs encoding members of the CK1 family. Western blot analysis was performed to assess the effects of overexpressed proteins, including VRK2, on the levels of IGFBP3 and pS201. **B** Cell lysates from RKO-derived CCSCs were subjected to immunoprecipitation using antibodies against IGFBP3 or IgG control, followed by Western blot to evaluate the interaction between VRK2 and IGFBP3. **C** Molecular docking analysis was conducted to model the interaction between IGFBP3 and VRK2 at the protein level. **D** Western blot analysis was performed to compare the expression levels of VRK2 protein in RKO cells and CCSCs. **E** CCSCs were transfected with VRK2-targeting siRNAs (siV-1, siV-2) or a negative control siRNA (siNC), and VRK2 expression was assessed by Western blot. **F** Expression levels of IGFBP3, pS201, and EGFR were analyzed by Western blot in CCSCs transfected with the aforementioned siRNAs. **G** sEVs were isolated from siRNA-transfected CCSCs, and the presence of IGFBP3, pS201, and EGFR in the sEVs was evaluated by Western blot. **H** RKO cells transfected with siNC or siVRK2 were treated with 5 μM oxaliplatin and 10 μM 5-FU for 48 h, and cell viability was measured using the CCK-8 assay. **I** Apoptotic rates of the above cells were determined using Annexin V-FITC/PI double staining followed by flow cytometry. **J** RKO cells transfected with siNC or siVRK2 were treated with 10 μM 5-FU for 48 h, and Western blot analysis was used to assess the expression of proteins involved in DNA damage and repair pathways. Data were presented as mean ± SD (*n* = 3; ns not significant, *P < 0.05, **P < 0.01, **P < 0.001).

To determine the phosphorylation mechanism of IGFBP3 in CCSCs, we explored the protein kinases involved in IGFBP3 modification. The NetPhos 3.1 tool predicted that the phosphorylation of the IGFBP3 S201 site (pS201) might be catalytically regulated by the casein kinase 1 (CK1) family of protein kinases (Supplementary Table 2). Using the iGPS software to analyze protein kinases at the IGFBP3 S201 site, we identified eight CK1 family members (CK1E, TTBK1, VRK3, CK1G1, TTBK2, VRK2, CK1D, and CK1G2) that might be involved in its phosphorylation modification (Supplementary Table 3). We overexpressed six CK1 family proteins in RKO cells (excluding TTBK1 and TTBK2, which are specifically expressed in brain tissue) and found that overexpression of VRK2 significantly enhanced IGFBP3 pS201 levels (Fig. 5A). Co-immunoprecipitation assays confirmed the interaction between IGFBP3 and VRK2 (Fig. 5B). The HDOCK Server predicted the binding interface between IGFBP3 and VRK2 (Fig. 5C), where the IGFBP3 S201 residue interacted with VRK2 Q201 through amino acid interactions. Notably, VRK2 expression in CCSC was higher than in tumor cells (Fig. 5D). To ascertain whether the elevated expression of VRK2 and phosphorylated IGFBP3 was primarily attributable to three-dimensional spheroid culture conditions or was an intrinsic characteristic of stemness, we conducted investigations in the sorted CD44-positive and negative RKO cells, as well as in CD44 + CCSC. Our findings revealed that the levels of VRK2 and pS201 were significantly higher in CD44 + RKO cells compared to their CD44- counterparts. Furthermore, both CCSC and CD44 + CCSC exhibited substantially elevated levels of VRK2 and pS201 when compared to RKO cells grown under conventional two-dimensional culture conditions (Supplementary Fig. 5E). Analyses of sEVs derived from both CCSC and CD44 + CCSC demonstrated a greater abundance of encapsulated pS201 and EGFR when compared to sEVs isolated from either CD44+ or CD44- RKO cells cultured in two-dimensional (Supplementary Fig. 5F). These findings indicate that the increased phosphorylation of IGFBP3 in CCSCs and their secreted CsEVs may be a synergistic outcome resulting from the stemness and the specific microenvironment provided by three-dimensional culture conditions.

Next, we knocked down VRK2 in CCSC cells using siRNA (Fig. 5E) and found that VRK2 knockdown reduced the levels of IGFBP3 pS201 both intracellularly (Fig. 5F) and in the secreted CsEVs (Fig. 5G). The use of siRNA targeting VRK2 in combination with chemotherapy drugs significantly inhibited RKO cell proliferation and promoted cell apoptosis compared to drug treatment alone (Fig. 5H, I). Furthermore, these siRNAs reduced the phosphorylation of EGFR and DNA-PKcs and increased γH2A.X, demonstrating a significant DSB driving effect (Fig. 5J). Therefore, VRK2 serves as the “origin” of phosphorylated IGFBP3/EGFR axis-mediated CCSC chemoresistance and may represent a potential target for reversing the resistance.

### Targeted inhibition of VRK2 restores chemotherapy sensitivity

**Fig. 6:**
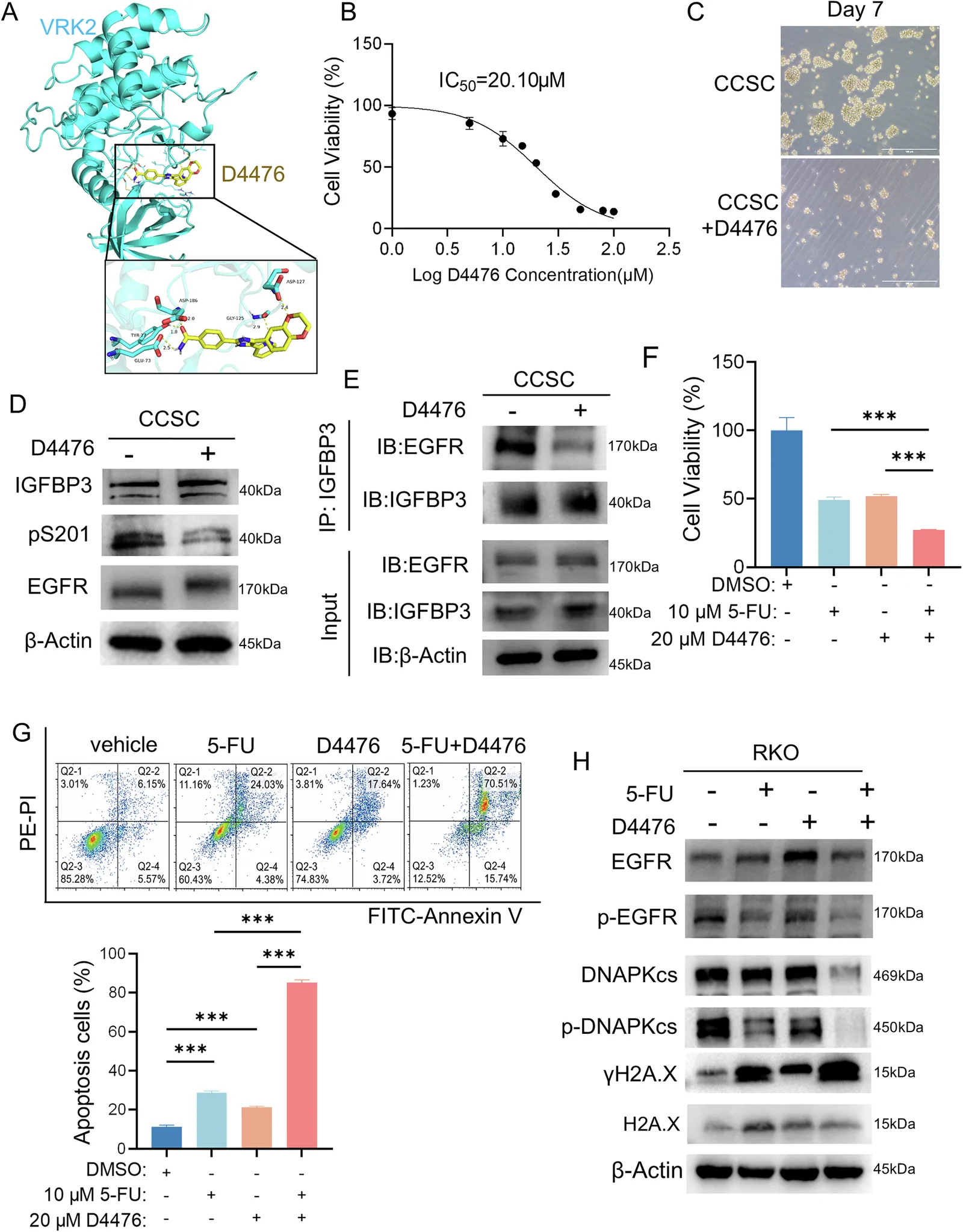
D4476 targeting the VRK2/IGFBP3 pathway enhances chemosensitivity in CRC. **A** Molecular docking of the VRK2 protein and D4476 compound. **B** CCSCs were treated with D4476 at concentrations of 1, 5, 10, 20, 50, and 100 μM for 48 h, and cell viability was assessed using the CCK-8 assay to calculate the half-maximal inhibitory concentration (IC50) for each concentration. **C** CCSCs were cultured with 20 μM D4476, and on day 7, the spheroid formation of CCSCs was observed under a microscope. **D** CCSCs were treated with 20 μM D4476, and Western blot analysis was performed to detect the expression levels of IGFBP3, pS201, and EGFR. **E** Immunoprecipitation assay was conducted to assess the interaction between EGFR and IGFBP3 in CCSCs after treatment with D4476. **F** RKO cells were treated with 10 μM 5-FU, 20 μM D4476 alone, or in combination for 48 h, and cell viability was measured using the CCK-8 assay. **G** Apoptosis was detected using Annexin V-FITC/PI double staining. **H** Western blot analysis was performed to detect protein expression in RKO cells treated with 5-FU, D4476, or their combination. Data were presented as mean ± SD (*n* = 3; ns not significant, **P < 0.01 and ***P < 0.001).

**Fig. 7:**
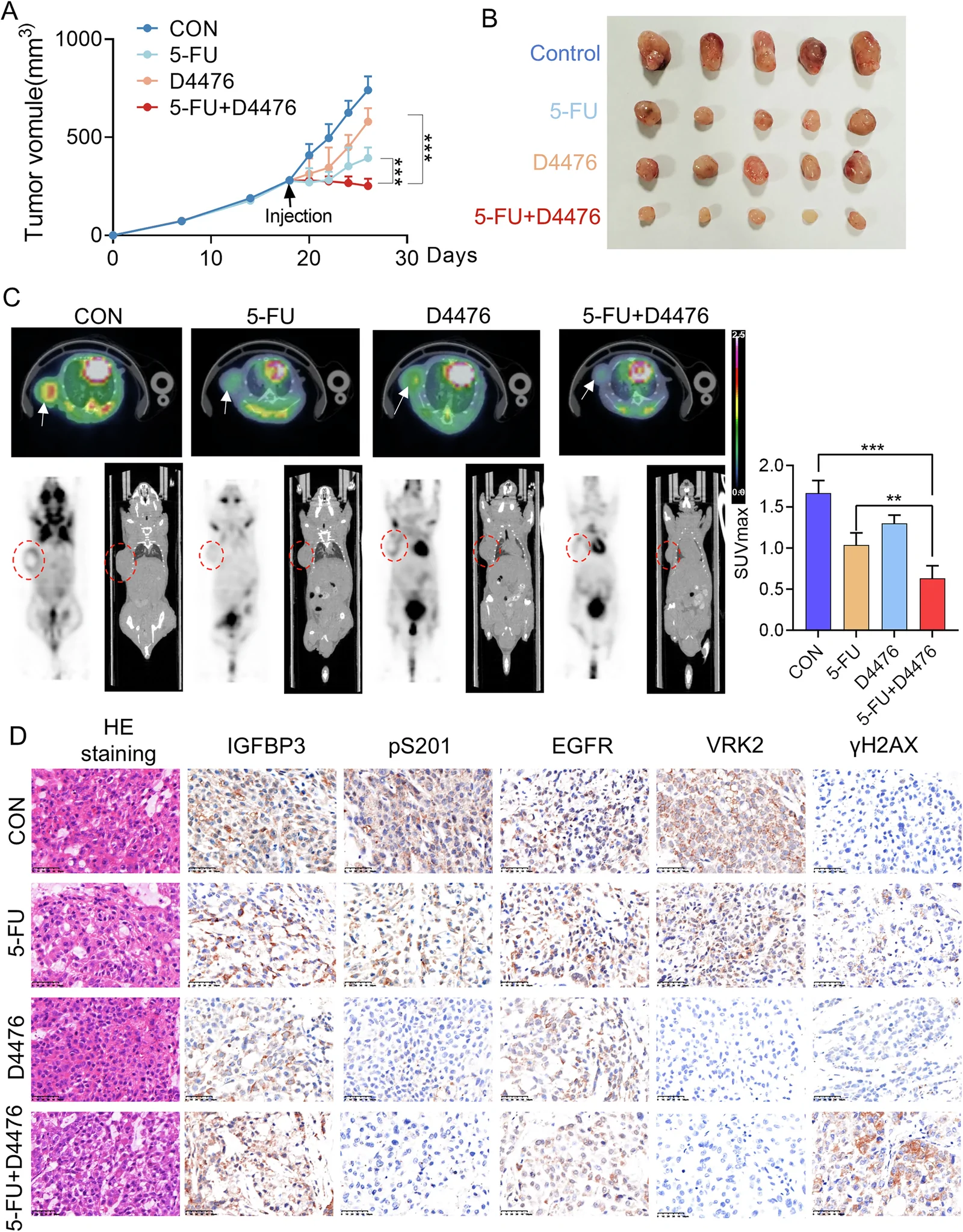
In vivo therapeutic effects of 5-FU combined with D4476 on CRC. **A**, **B** Subcutaneous tumor implantation in mice using RKO cells expressing luciferase, followed by treatment with 10 mg/kg 5-FU, 20 mg/kg D4476, or 10 mg/kg 5-FU + 20 mg/kg D4476 for 10 days. Tumor size and volume were recorded and photographed. **C** Representative ^18^F-FDG PET/CT images of tumor-bearing mice and the maximum standardized uptake value (SUVmax) were calculated. **D** H&E staining of tumor tissues, and expression of IGFBP3, pS201, EGFR, VRK2, and γH2AX was detected by IHC. Data were presented as mean ± SD (*n* = 5; ns not significant, **P < 0.01 and ***P < 0.001).

In addition to siRNA, appropriate pharmacological tools targeting VRK2 are necessary to enhance the efficacy of chemotherapy. D4476 is a selective CK1 inhibitor that broadly inhibits the phosphorylation of intracellular proteins [30]. Autodock molecular docking revealed that D4476 formed hydrogen bond interactions with the VRK2 protein at residues E73, Y77, G125, D127, and D186 (Fig. 6A). We found that D4476 exhibited growth inhibition effects on CCSCs (IC50 = 20.1 μM, Fig. 6B) and suppressed the stemness and sphere formation of CCSCs (Fig. 6C). As a potential VRK2 inhibitor, D4476 reduced the level of IGFBP3 pS201 in CCSCs (Fig. 6D) and disrupted the interaction between IGFBP3 and EGFR (Fig. 6E). In terms of drug treatment, compared to 5-FU alone, the combination of D4476 and 5-FU significantly decreased RKO and CCSC cell proliferation and increased apoptosis (Fig. 6F, G and Supplementary Fig. 5G). Furthermore, the combination of D4476 and 5-FU promoted the occurrence of DSBs in RKO cells (Fig. 6H). In vivo, the combination treatment of D4476 and 5-FU effectively suppressed the growth of subcutaneous RKO-based CRC tumors in mice (Fig. 7A, B). Bioluminescence imaging and ^18^F-FDG PET/CT imaging demonstrated that the combined administration of D4476 and 5-FU inhibited both tumor growth and glucose metabolism (Fig. 7C and Supplementary Fig. 5H). Immunohistochemical analysis of tumor tissues revealed that the combination therapy reduced the expression levels of VRK2 and IGFBP3 pS201, while promoting the γH2A.X expression indicative of DSBs (Fig. 7D). As a targeted pharmacological approach for VRK2, D4476 holds promise as a strategy to reverse chemotherapy resistance.

### CRC patients with high expression of phosphorylated IGFBP3 have potential chemoresistance

**Fig. 8:**
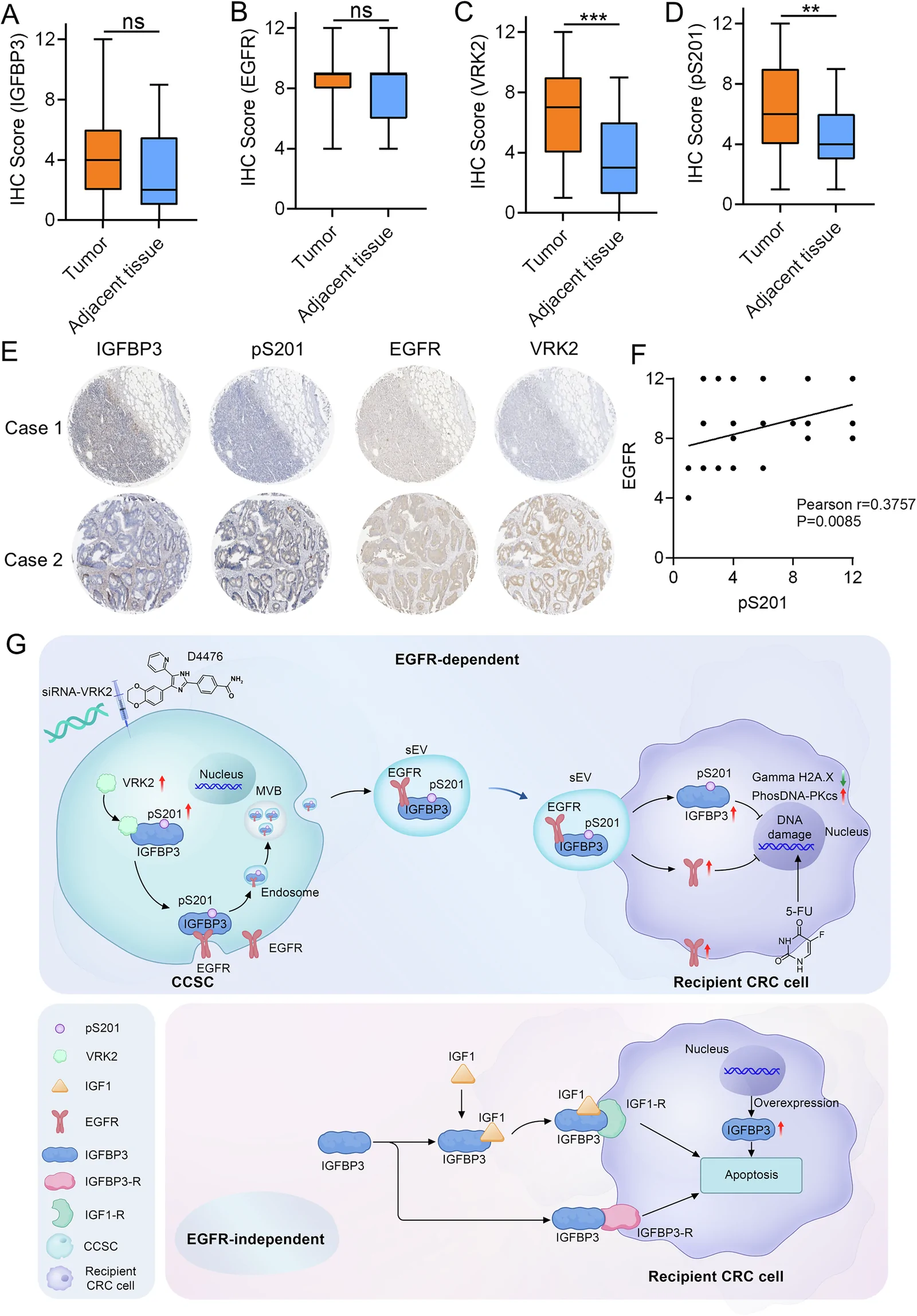
Correlation between phosphorylated IGFBP3, EGFR, and VRK2 expression in human colorectal cancer tissues. **A**–**D** Quantitative immunohistochemical analysis of IGFBP3, EGFR, VRK2, and pS201 expression in cancerous and adjacent non-cancerous tissues in human CRC tissue microarrays. Data were presented as mean ± SD (*n* = 48; ns not significant, **P < 0.01, ***P < 0.001). **E** Representative images of IGFBP3, EGFR, VRK2, and pS201 expression in the cancer tissues from two patients. **F** Pearson correlation analysis of the expression correlations between pS201 and EGFR in cancer tissues. **G** Schematic diagram illustrating the mechanism by which EGFR-dependent secretion of sEVs containing IGFBP3 pS201 promotes CRC chemoresistance in CCSCs. Poorly differentiated CCSCs were shown to deliver sEVs to well-differentiated CRC cells, which enhanced DNA damage repair under chemotherapy and increased resistance. Targeting the VRK2 kinase with siRNA or inhibitors could potentially address the source of CCSC-derived sEVs, offering a strategy to improve chemotherapy sensitivity. CRC colorectal cancer, CCSC colorectal cancer stem cell-like spheroids cells, MVB multivesicular bodies, sEV small extracellular vesicle, pS201 phosphorylation of IGFBP3 at the serine 201 site, IGF1 insulin-like growth factor 1, IGF1-R IGF1 receptor, IGFBP3 insulin-like growth factor binding protein 3, IGFBP3R IGFBP3 receptor.

We validated the EGFR-dependent IGFBP3 phosphorylation pathway in clinical CRC tissues. According to the RNA-seq expression data from the TCGA public database, IGFBP3 showed minimal expression differences across various tumors and normal tissues, including COAD and READ (Supplementary Fig. 6A). We collected cancer tissues and adjacent non-cancerous tissues from 48 CRC patients and found that the protein levels of IGFBP3 and EGFR showed no significant difference between tumors and adjacent tissues (Fig. 8A, B). Additionally, the protein kinase VRK2 was significantly upregulated in cancer (Fig. 8C). These results were generally consistent with the RNA expression in TCGA data conducted using the GEPIA tool (Supplementary Fig. 6B–D). Importantly, the expression level of IGFBP3 phosphorylated at S201 (pS201) was significantly higher in cancer tissues compared to adjacent non-cancerous tissues (Fig. 8D). We observed that pS201, EGFR, and VRK2 were concurrently upregulated or downregulated in the tumor tissues of certain patients (Fig. 8E). Correlation analysis revealed no significant association between EGFR and IGFBP3 expression, nor between VRK2 and IGFBP3 expression in cancer tissues (Supplementary Fig. 6E, F). However, significant correlations were identified between EGFR and pS201 expression, as well as between VRK2 and pS201 expression (*p* < 0.05; Fig. 8F and Supplementary Fig. 6G). Based on these findings, we propose a model in which an EGFR-dependent phosphorylation of IGFBP3 occurs in CRC tissues (Fig. 8G). This phosphorylation is driven by VRK2, which is highly expressed in CCSCs, ultimately contributing to chemoresistance in CRC.

## Discussion

CRC is characterized as a highly heterogeneous tumor type, and the chemotherapy resistance and recurrence associated with it derive from the intrinsic resistance conferred by CCSCs. CCSCs, similar to CSC-like cells in other tumor types, exhibit phenotypic plasticity that allows them to transition into a resistant phenotype under the influence of various stimuli. These stimuli include extracellular vesicles or exosomes. Fibroblast-derived sEVs induced the dedifferentiation of CCSCs, thereby promoting chemotherapy resistance in CRC [31]. In our study, poorly differentiated CCSCs were found to deliver sEVs to well-differentiated CRC cells. Although this interaction did not alter the differentiation status of the CRC cells, it enhanced the DNA damage repair in recipient CRC cells under chemotherapy, leading to increased resistance. As a therapeutic approach, targeting the VRK2 kinase with siRNA or inhibitor offers a potential strategy for addressing the “source” of CCSC-derived sEVs, showing promise in improving chemotherapy sensitivity (Fig. 8G). Employing treatments that target CCSC-derived sEVs necessitates the confirmation of a patient’s classification as having a high distribution of poorly differentiated CCSCs or elevated levels of CCSC sEVs within the microenvironment. Nanotechnology provides us with viable tools for the detection of CCSC-derived sEVs [32], and this non-invasive diagnostic approach also opens up possibilities for targeted CCSC therapies.

Although sEVs derived from sources such as CSCs, resistant cells, immune cells, and fibroblasts are being demonstrated as contributors to tumor resistance, the occurrence of chemotherapy resistance may be a multi-stage process driven by sEVs. Recent perspectives suggest that the core of resistance evolution lies in the initiation of phenotypic adaptation mechanisms by cells that do not harbor resistance mutations or sensitive cells under drug pressure, aiming for survival [33]. This adaptation includes genetic mutations, changes in protein excretion, metabolic reprogramming, and remodeling of the microenvironment, with sEVs being comprehensively involved in resistance evolution. For example, sEVs from tumor cells carry KRAS G12D or metabolically regulatory circRNA, promoting the chemotherapy resistance of pancreatic cancer to gemcitabine [34, 35]. Our research indicates that CCSCs with resistant characteristics extrude EGFR and phosphorylated IGFBP3 via sEVs, and that these extruded proteins drive resistance. In fact, preparations for resistance commence in sEVs shortly after the initiation of drug treatment. Studies have shown that docetaxel induced breast cancer cells to secrete sEVs containing stemness-regulating RNAs shortly after treatment onset, leading to sustained activation of CSC formation [15]. Therefore, sEVs are intricately involved in every aspect of resistance, and these multifunctional roles contribute to the heterogeneity of tumor resistance. This also elucidates the formidable environmental adaptability of tumor cells, which poses significant challenges for effective treatment.

The high heterogeneity of drug resistance driven by CSC-like cell or other cell-derived sEVs is also attributed to the variability in cargo loading. IGFBP3 cargo is one of the regulatory proteins involved in this heterogeneity. IGFBP3 exists within tumor cells, tumor endothelial cells, or as an extracellular soluble protein, and has traditionally been considered a tumor-suppressive protein that promotes apoptosis [36, 37]. However, in paired clinical tumor samples, we found that IGFBP3 did not show differential expression compared to normal tissues in the majority of cancer types. High levels of secreted IGFBP3 and the IGF1 ligand in the blood of CRC patients are associated with an increased risk of CRC, suggesting that IGFBP3 has distinct regulatory roles in CRC progression [19]. We propose that in drug-resistant or highly metabolic CRC tissues, IGFBP3 exerts oncogenic effects. In CRC tissues with mitochondrial metabolic dysfunction, particularly in CRC liver metastasis, IGFBP3 expression is higher compared to adjacent tissues [38]. Our research demonstrates that in CRC types with high EGFR expression, IGFBP3 and its phosphorylated forms promote chemotherapy resistance. EGFR inhibitors have been found to suppress IGFBP3 function, thus disrupting olaparib resistance [28]. We attribute the EGFR-dependent resistance activity of IGFBP3 to its phosphorylation at the S201 site, revealing the critical role of this activation site in CSC-driven resistance.

EGFR is widely overexpressed in CRC; however, the clinical benefit of EGFR inhibitors in CRC treatment is often limited in duration. The mechanisms underlying this resistance may involve KRAS mutations, BRAF mutations, or CSC-related pathways [39]. EGFR signaling plays a critical role in driving CRC tumorigenesis and progression within CSC populations [40]. We employed the VRK2 inhibitor D4476 as a pharmacological tool to counteract CSC-like cells functionality and restore chemosensitivity, which may be particularly applicable in EGFR-positive CRC subtypes. Therefore, the assessment of EGFR and CSC marker expression may be essential for guiding CRC treatment strategies. One limitation of this study is the absence of a direct comparison between the therapeutic efficacy of EGFR inhibitors and D4476, as D4476 is considered a more selective inhibitor of IGFBP3 phosphorylation. Given the rarity of EGFR mutations in CRC, EGFR inhibitors tend to be more effective in tumor types harboring such mutations [41, 42]. Another limitation of this study is that we did not clearly distinguish whether the therapy resistance mediated by CCSC-derived sEVs originates from the intrinsic quiescent state of CSCs or is induced by the three-dimensional (3D) culture conditions. In this study, we defined CCSCs as CSC-like spheroids, as they are derived from 3D culture systems and exhibit partial stemness features. It should be noted that 3D-cultured cells or organoid models may contain non-CSC populations, which can remain sensitive to therapeutic interventions [43]. Nevertheless, 3D culture conditions are known to promote the growth and survival of CSCs, thereby enhancing the overall stemness of the tumor cell population [44]. Our findings suggest that the elevated phosphorylation of IGFBP3 in CCSC-derived sEVs is associated with both 3D culture conditions and increased stemness characteristics. However, due to technical limitations, we were unable to obtain 3D cultures with lower stemness features for direct comparison. In this regard, patient-derived organoid models may provide a more physiologically relevant system to further address this issue in future studies. Consequently, the combined inhibition of EGFR and IGFBP3 may represent a promising therapeutic approach for CRC and warrants further investigation.

## Supplementary information

Supplementary Figures

Supplementary Table 1

Supplementary Table 2

Supplementary Table 3

original Western blots

## Data Availability

The remaining data and original Western blots of this study are included in the Supplementary files. Further data supporting the results of this study can be provided by the corresponding author upon reasonable request.
