# Supplementary material for: EGFR-dependent delivery of phosphorylated IGFBP3 by cancer stem cells drives chemotherapy resistance in colorectal cancer
Source: Cell Death Dis. 2026 Jun 29;17(1):804. doi: 10.1038/s41419-026-09009-0 (PMC13578265; doi:10.1038/s41419-026-09009-0)

Fig1 G

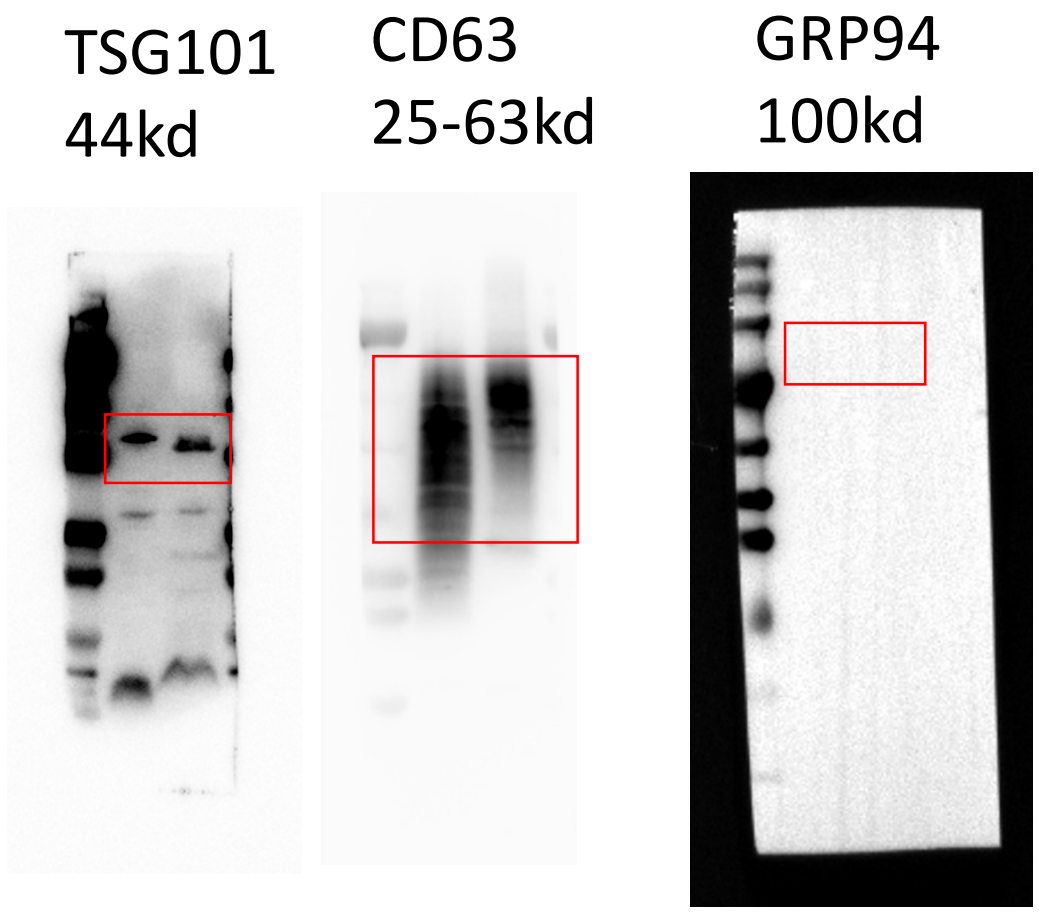

Fig2 F

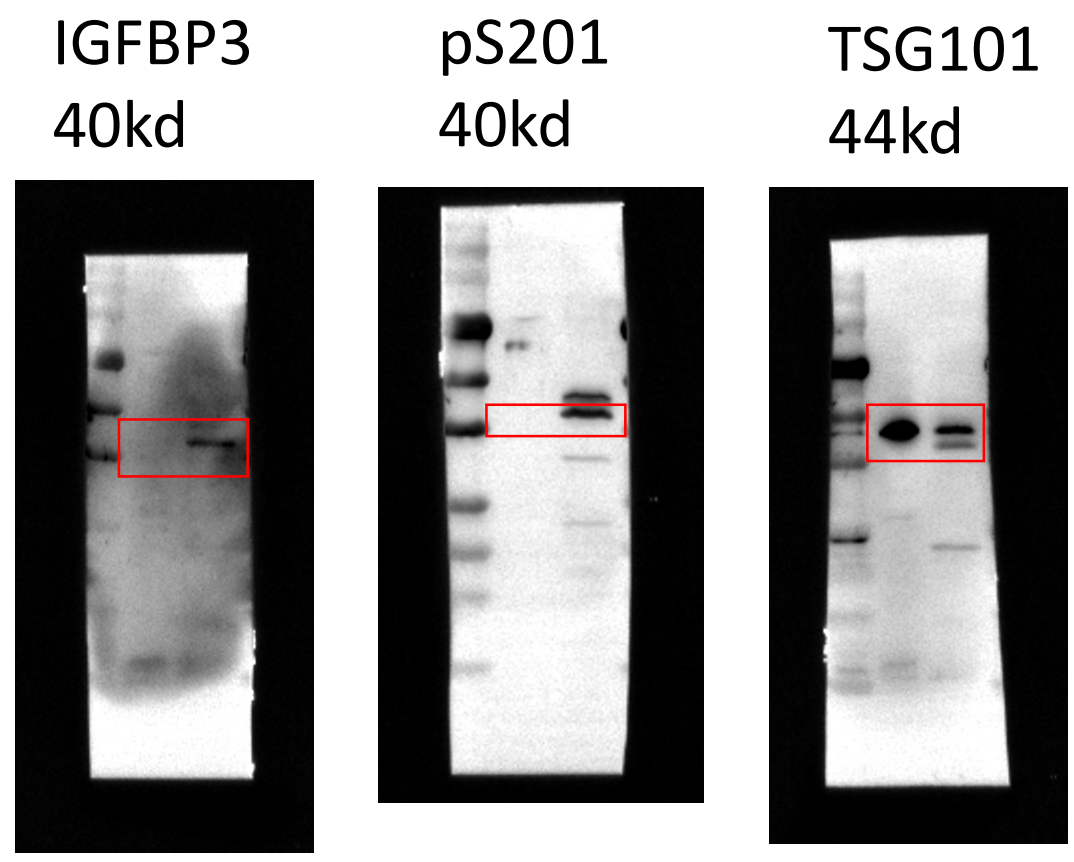

Fig2 G

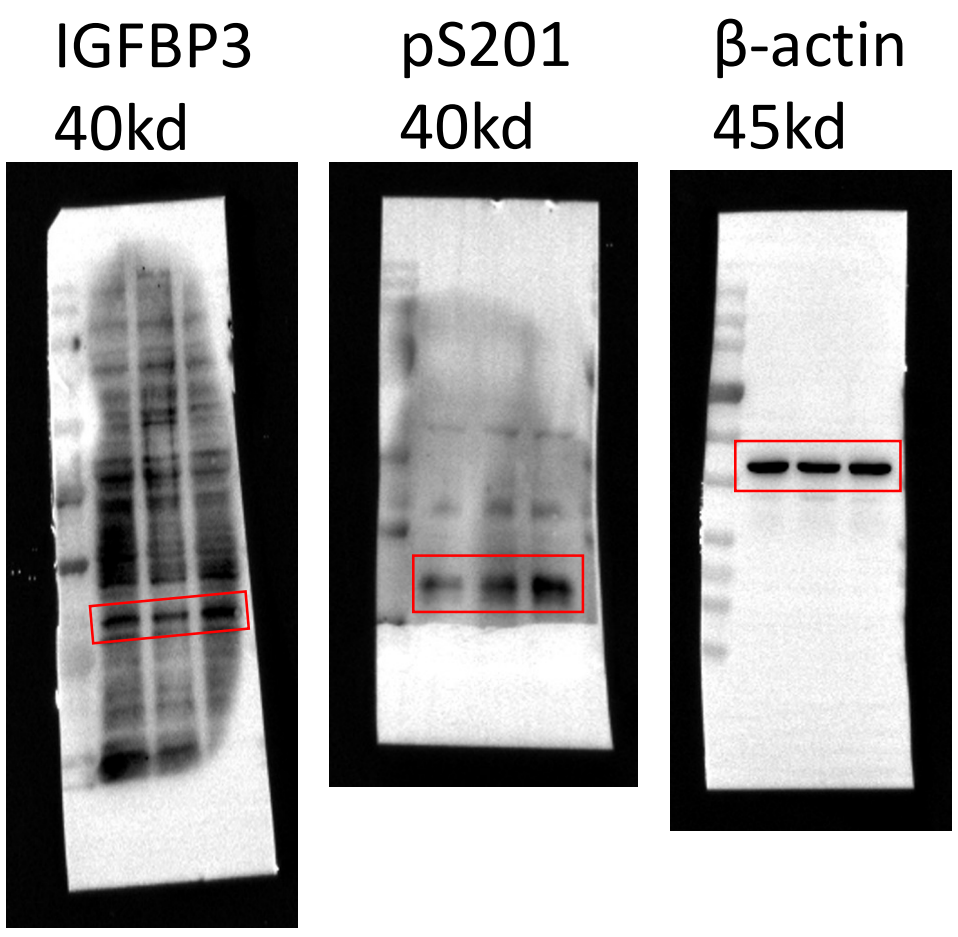

Fig3 A

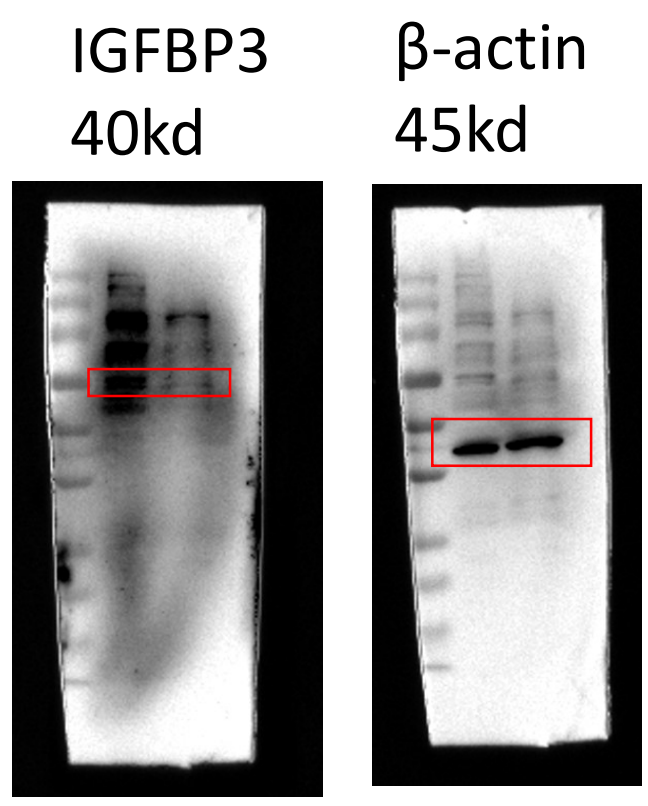

Fig3 D

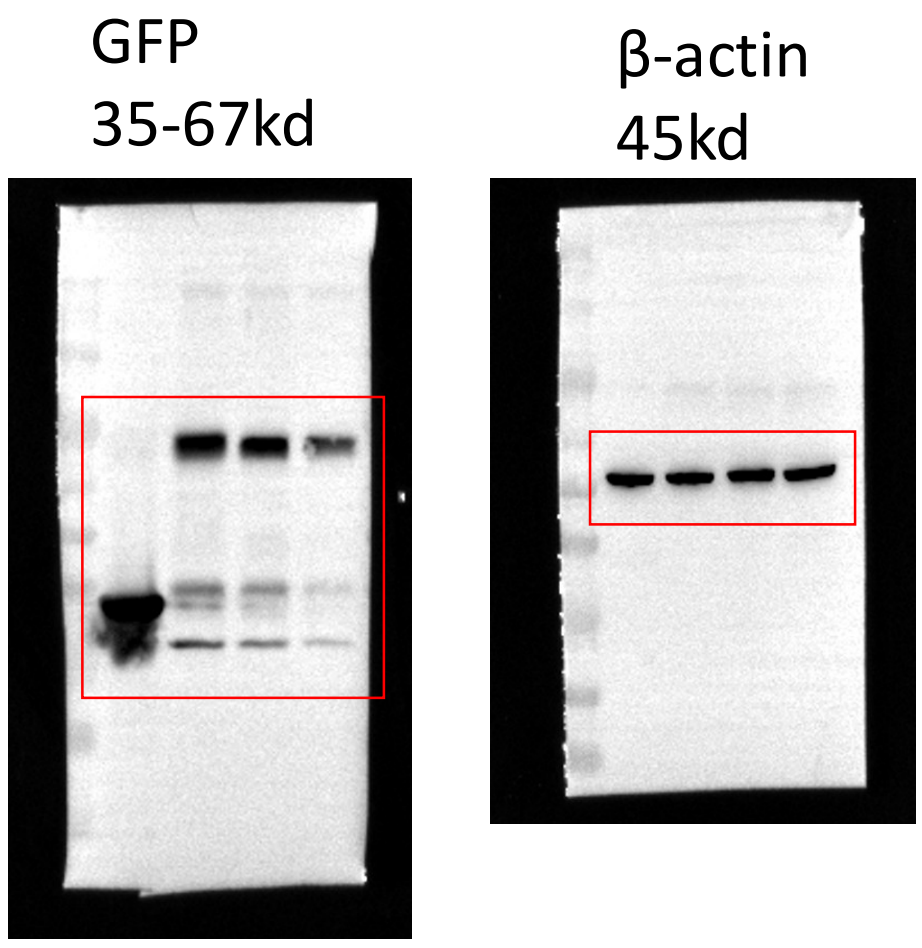

Fig3 H

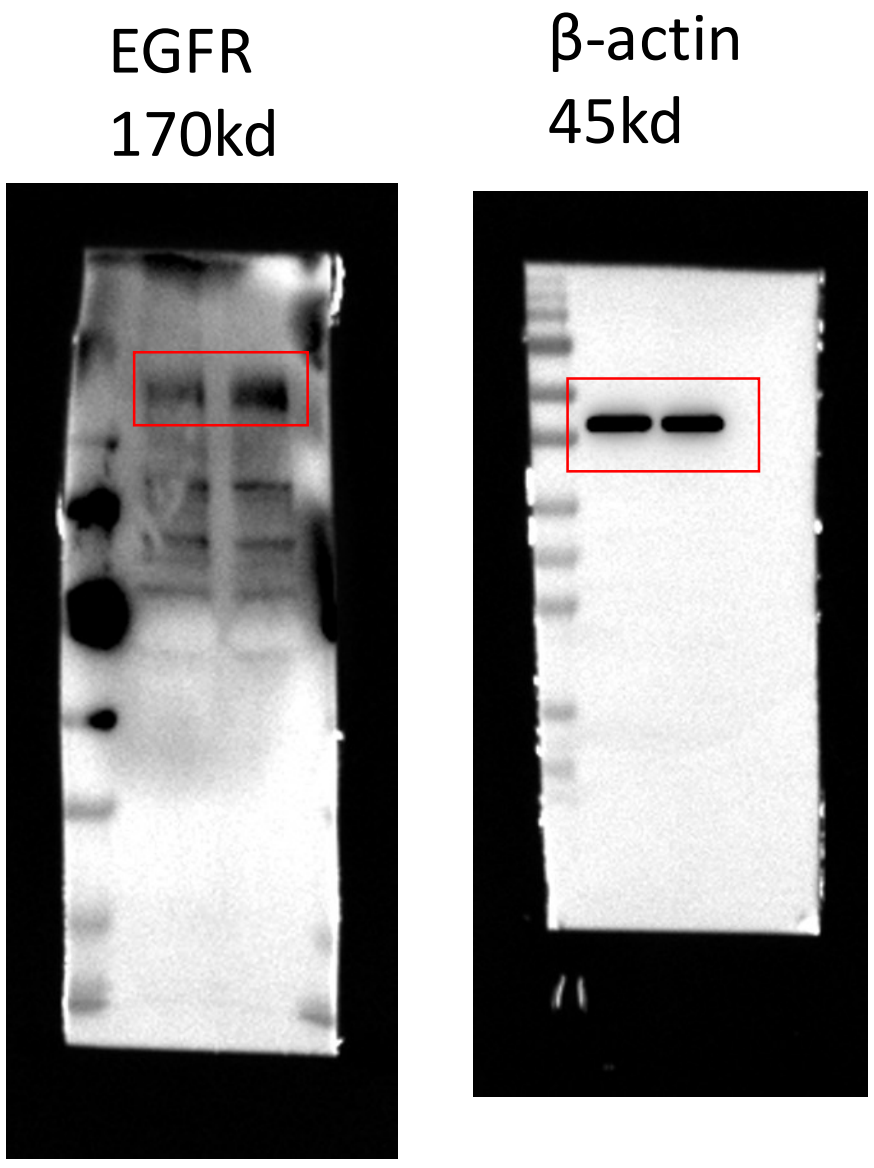

Fig3 I

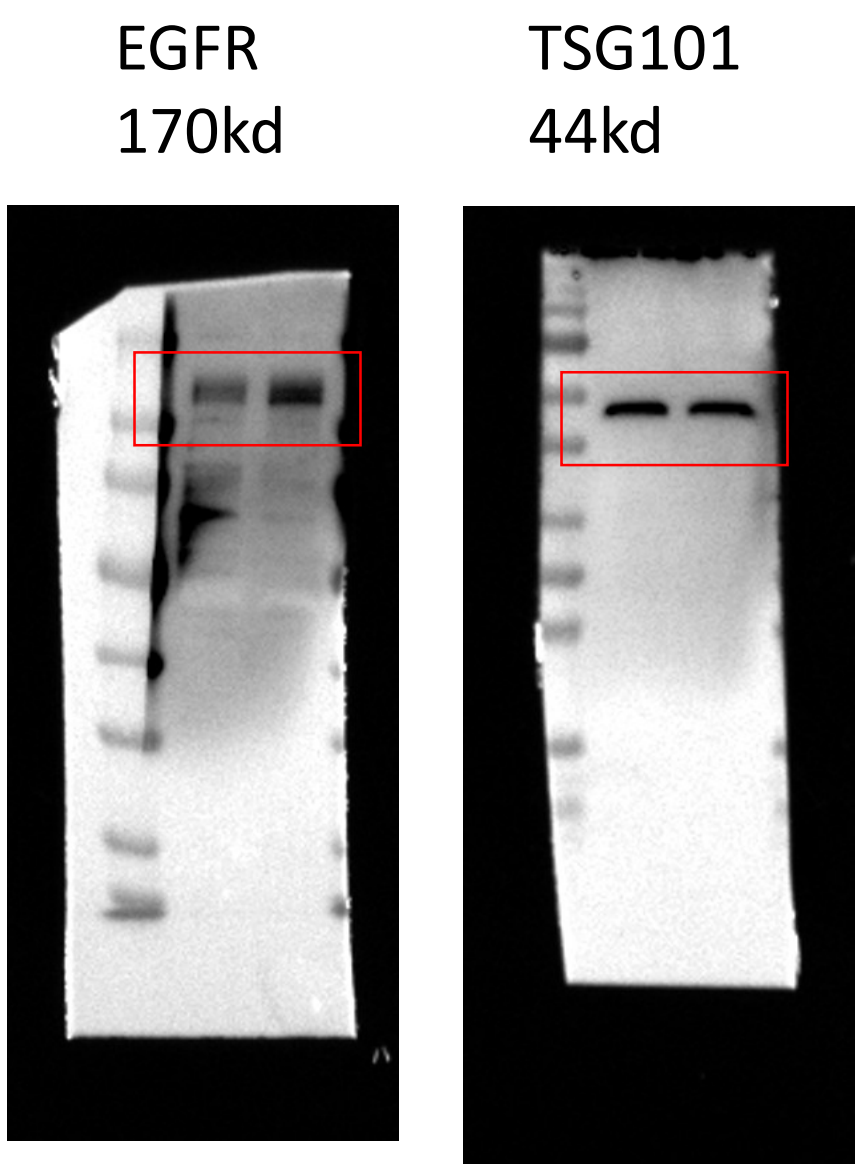

Fig4 A

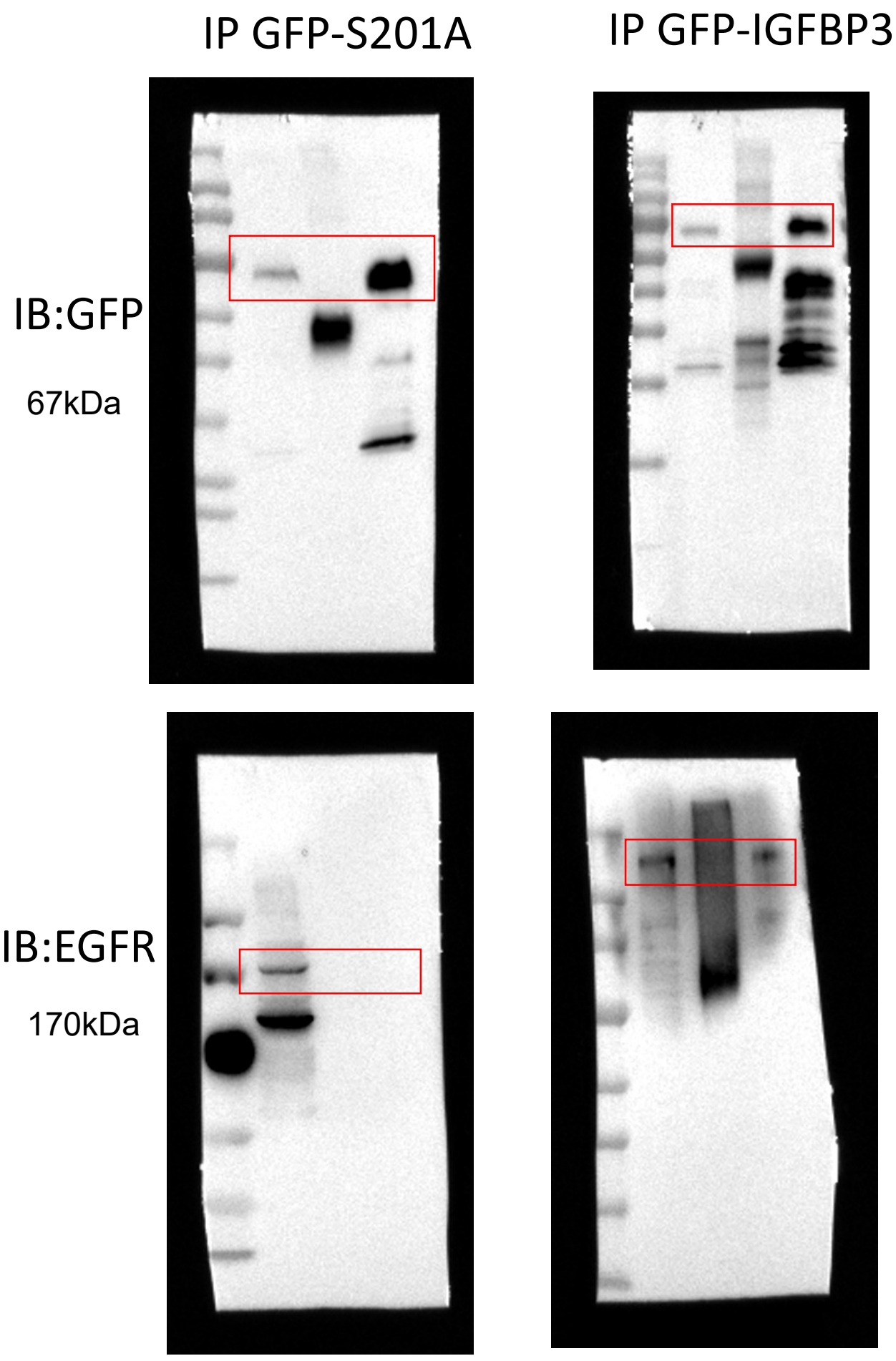

Fig4 D

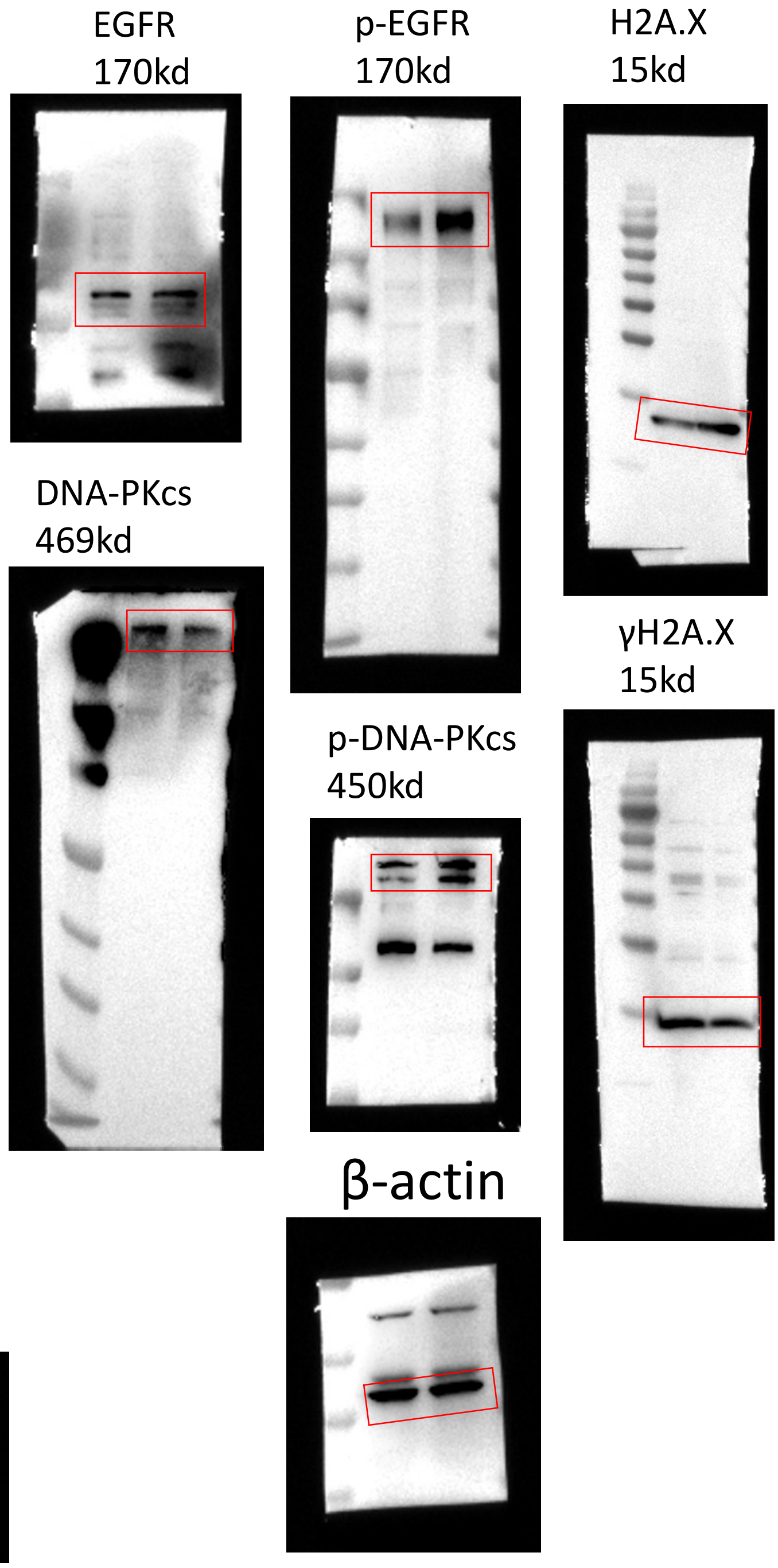

Fig4 F

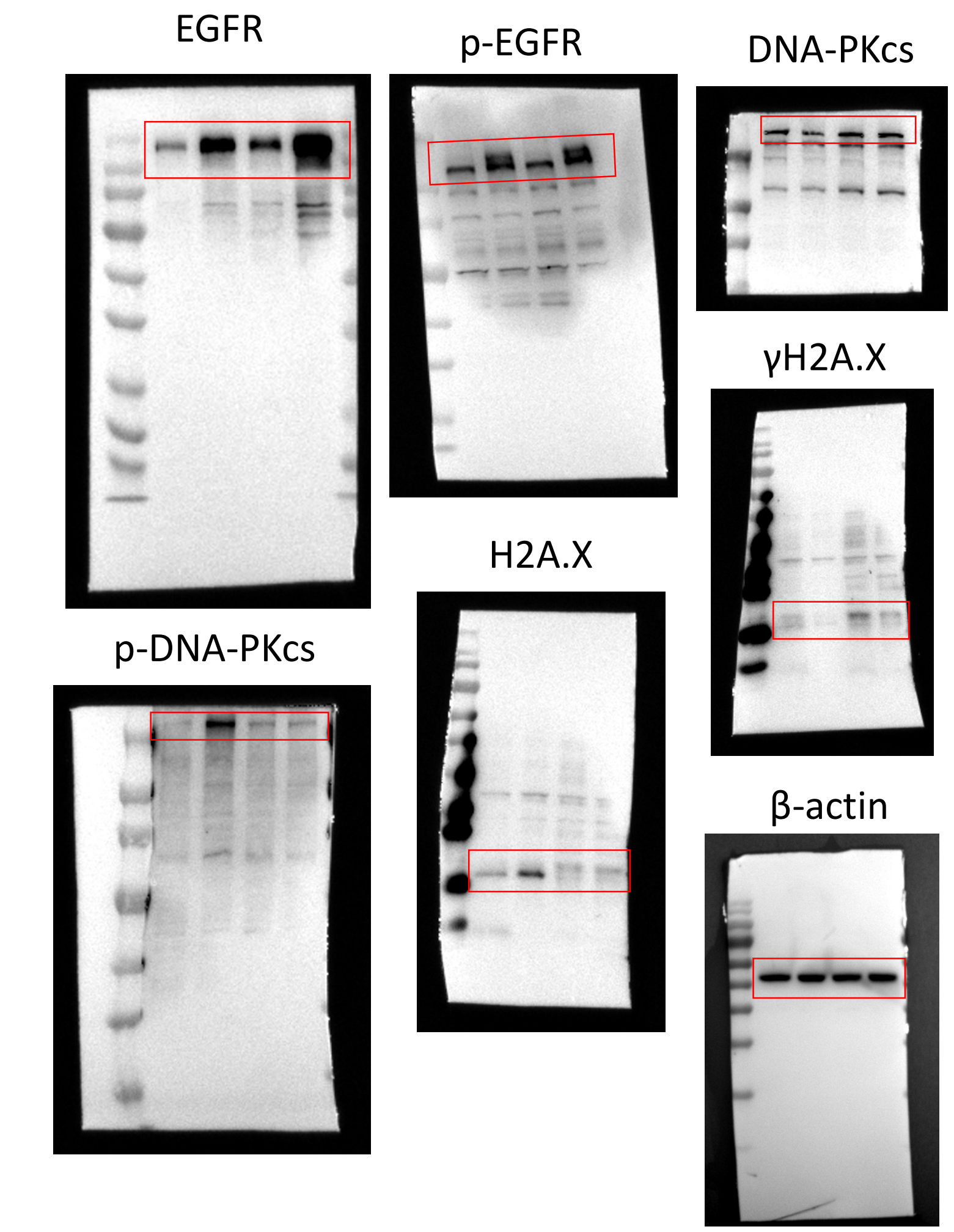

Fig4 G

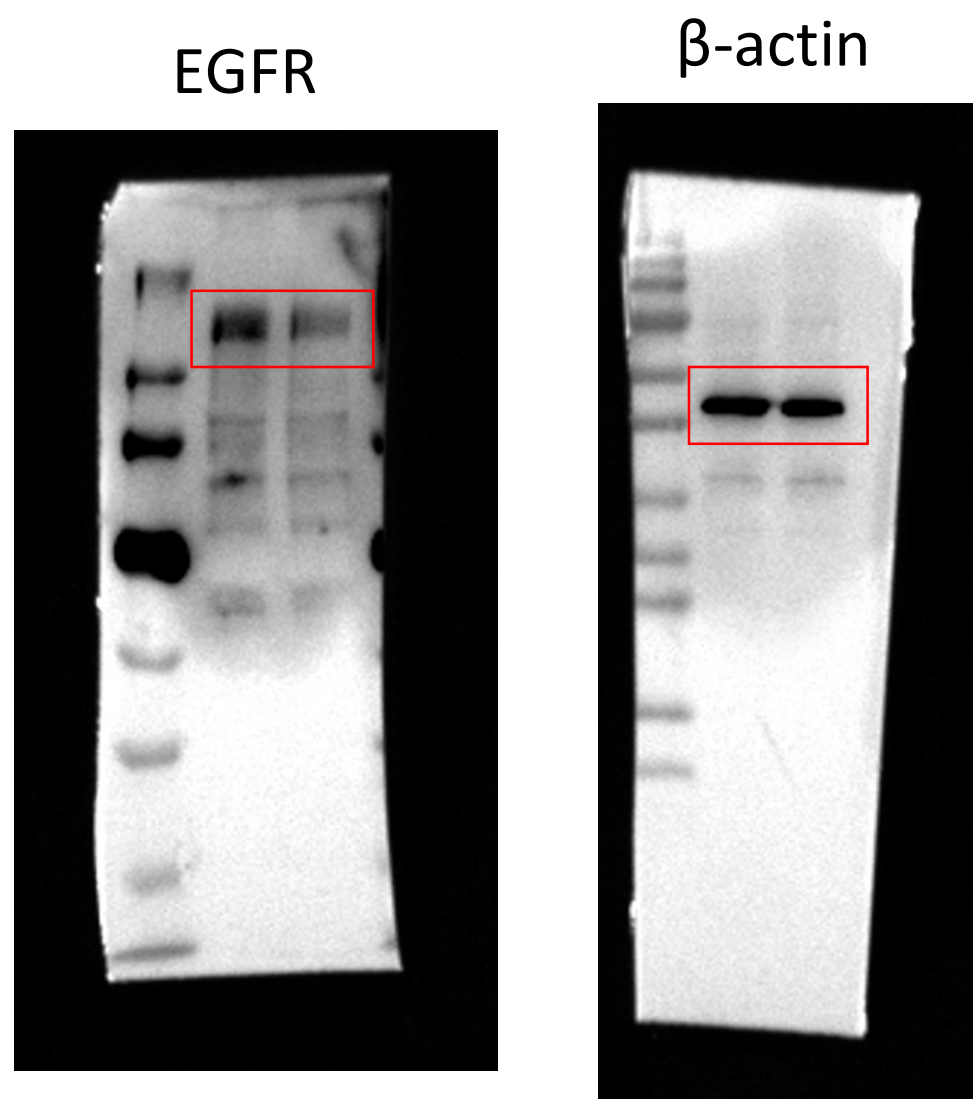

Fig5 A

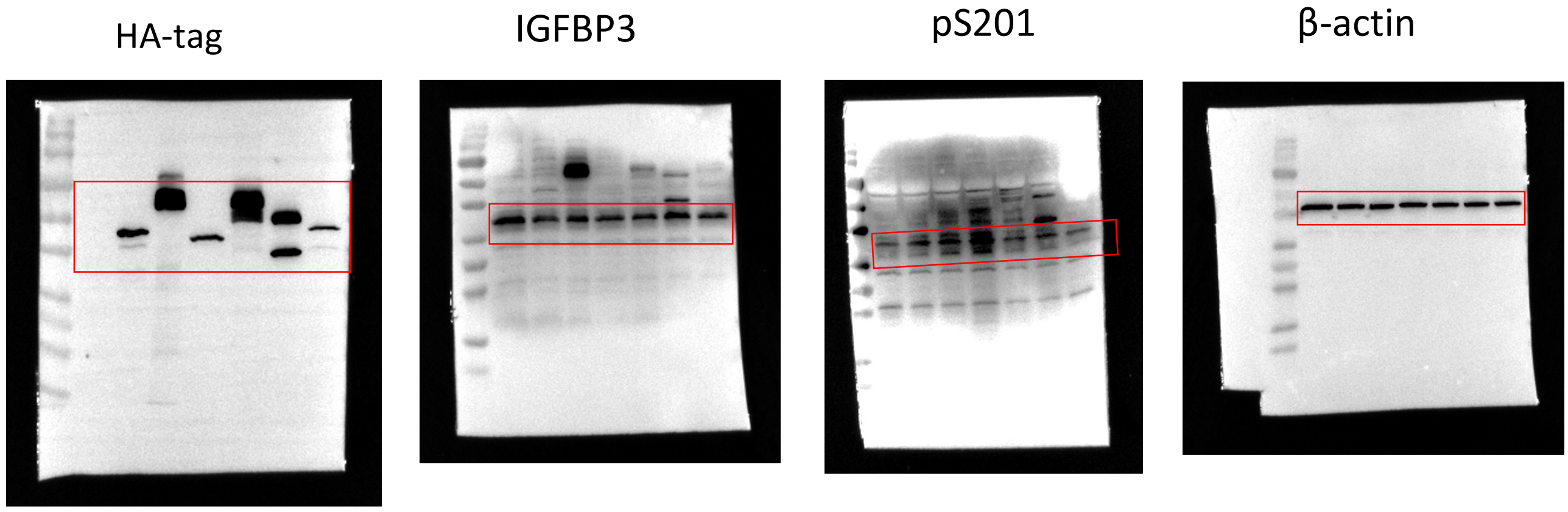

Fig5 B

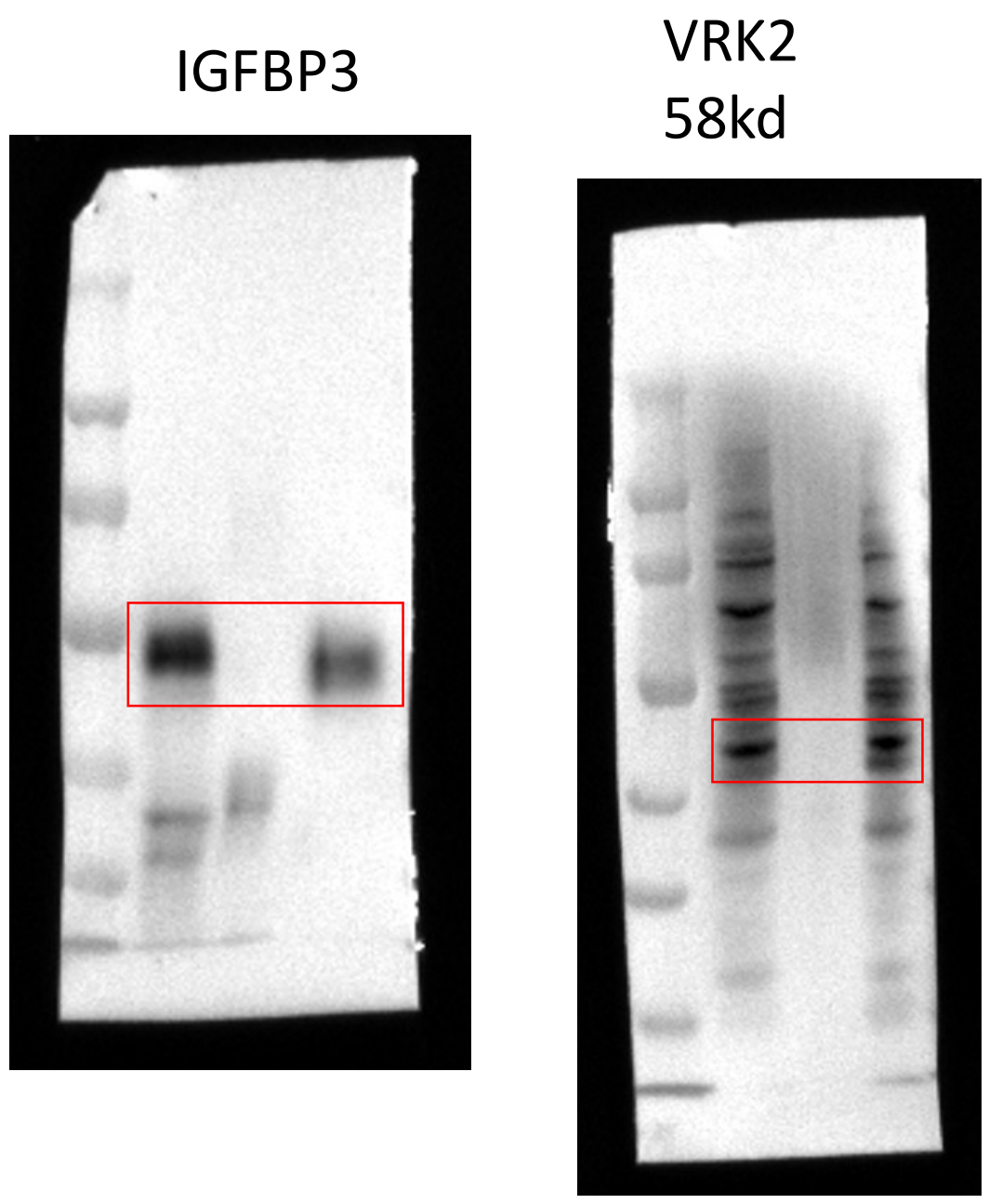

Fig5 D

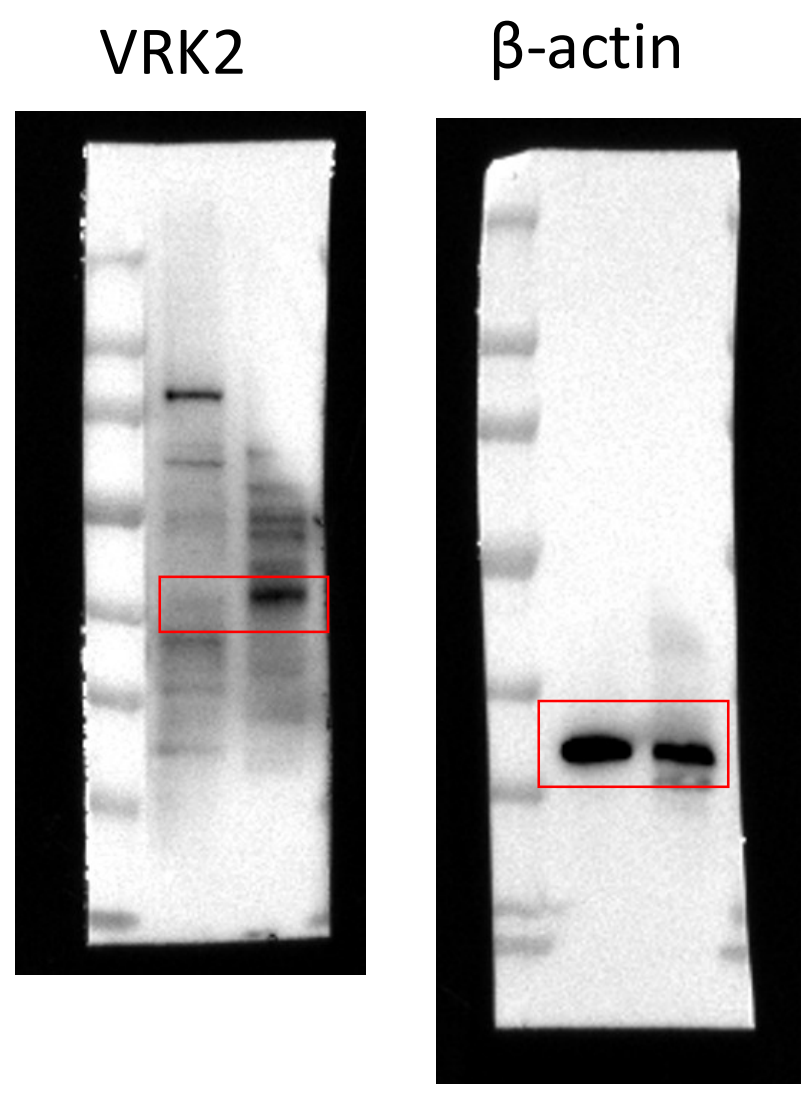

Fig5 E

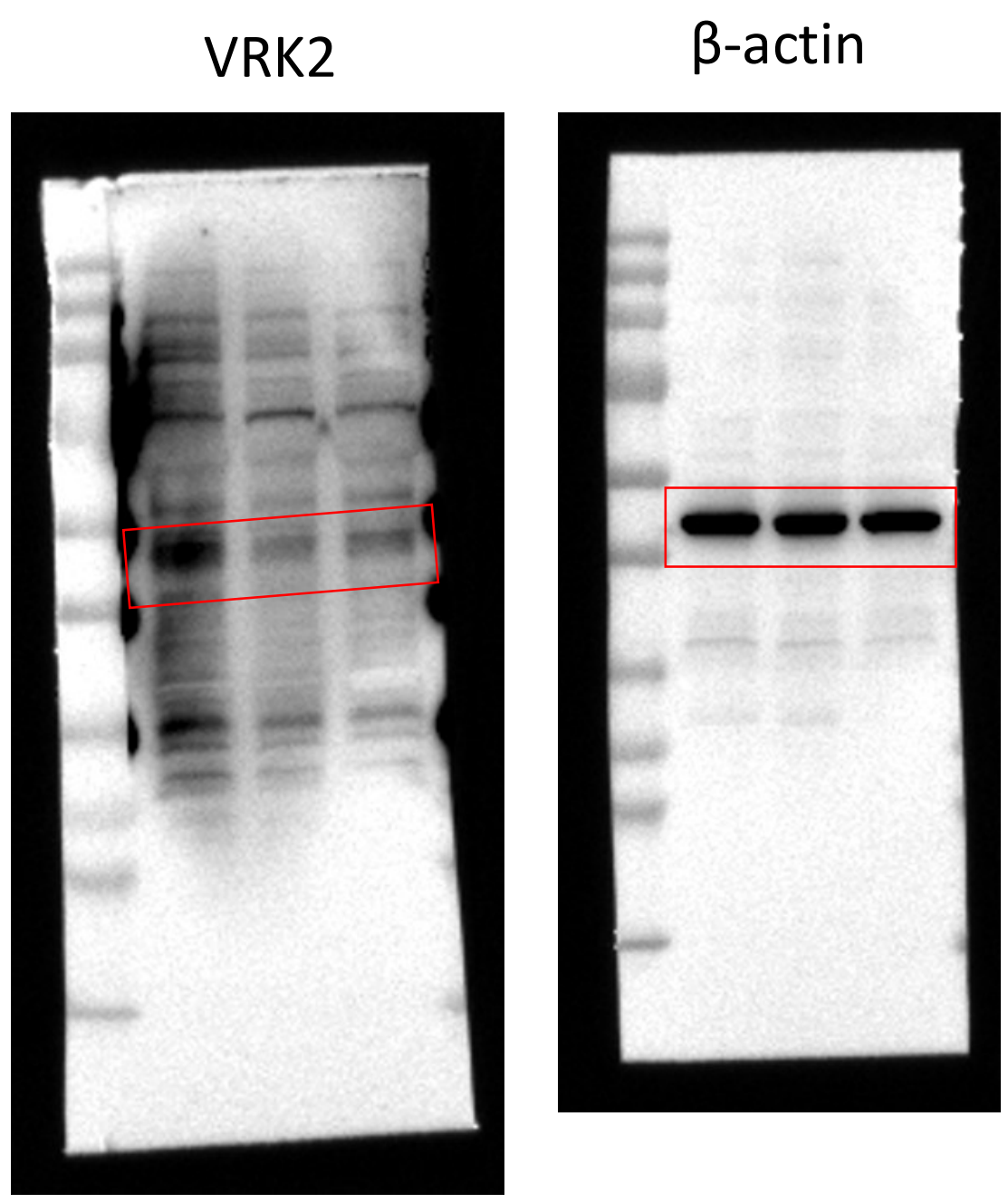

Fig5 F

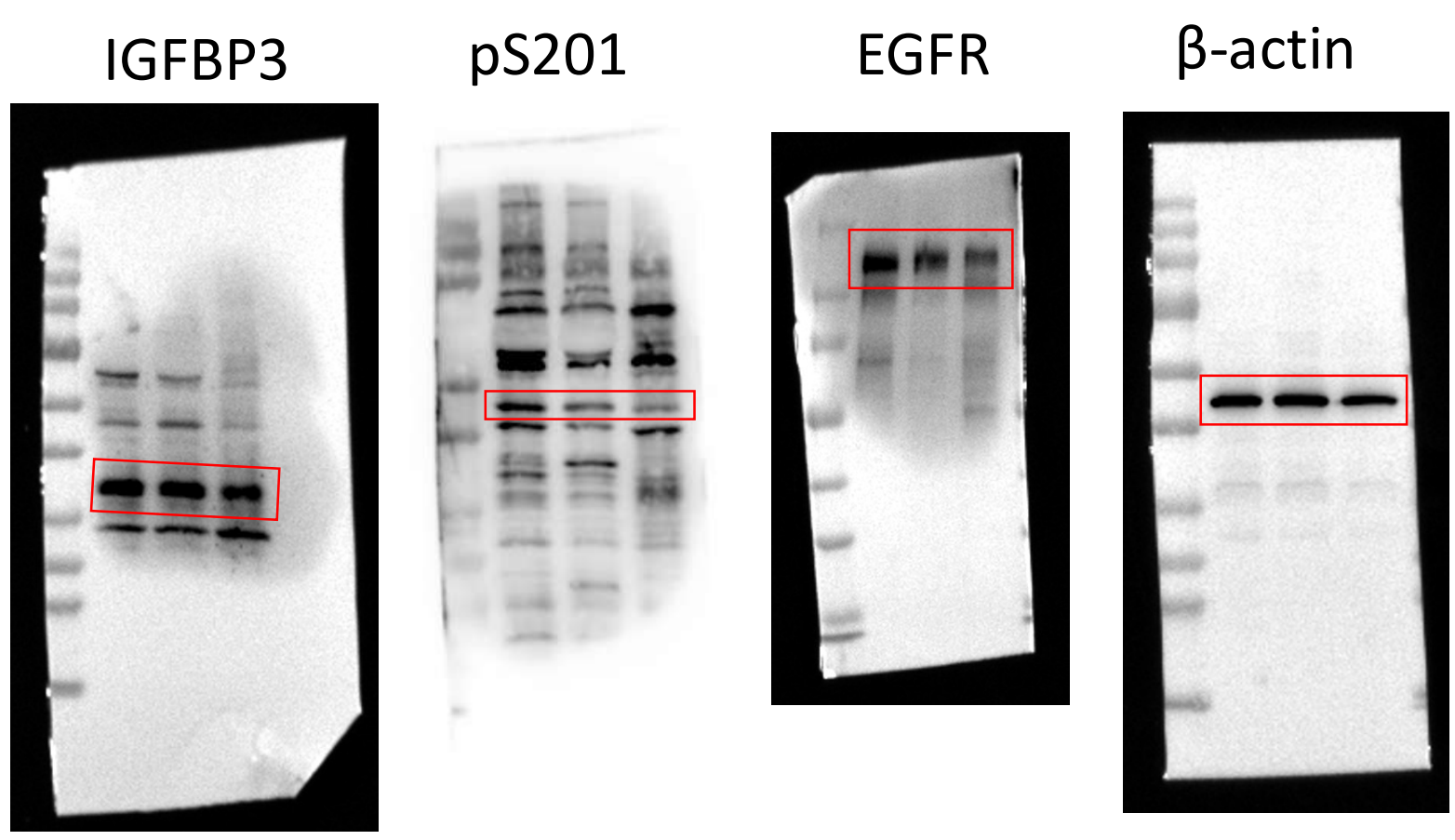

Fig5 G

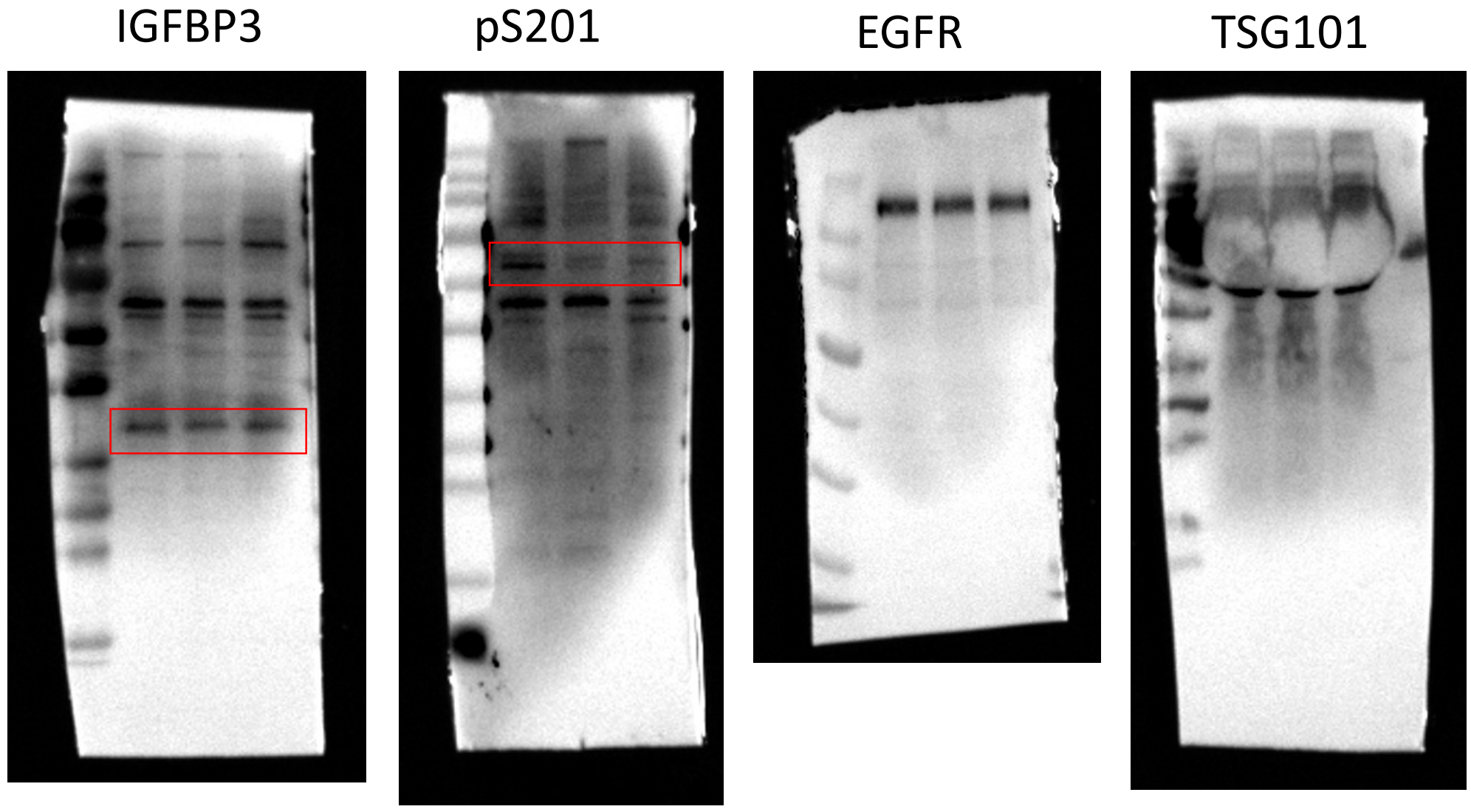
IGFBP3
pS201
EGFR
TSG101

Fig5 J

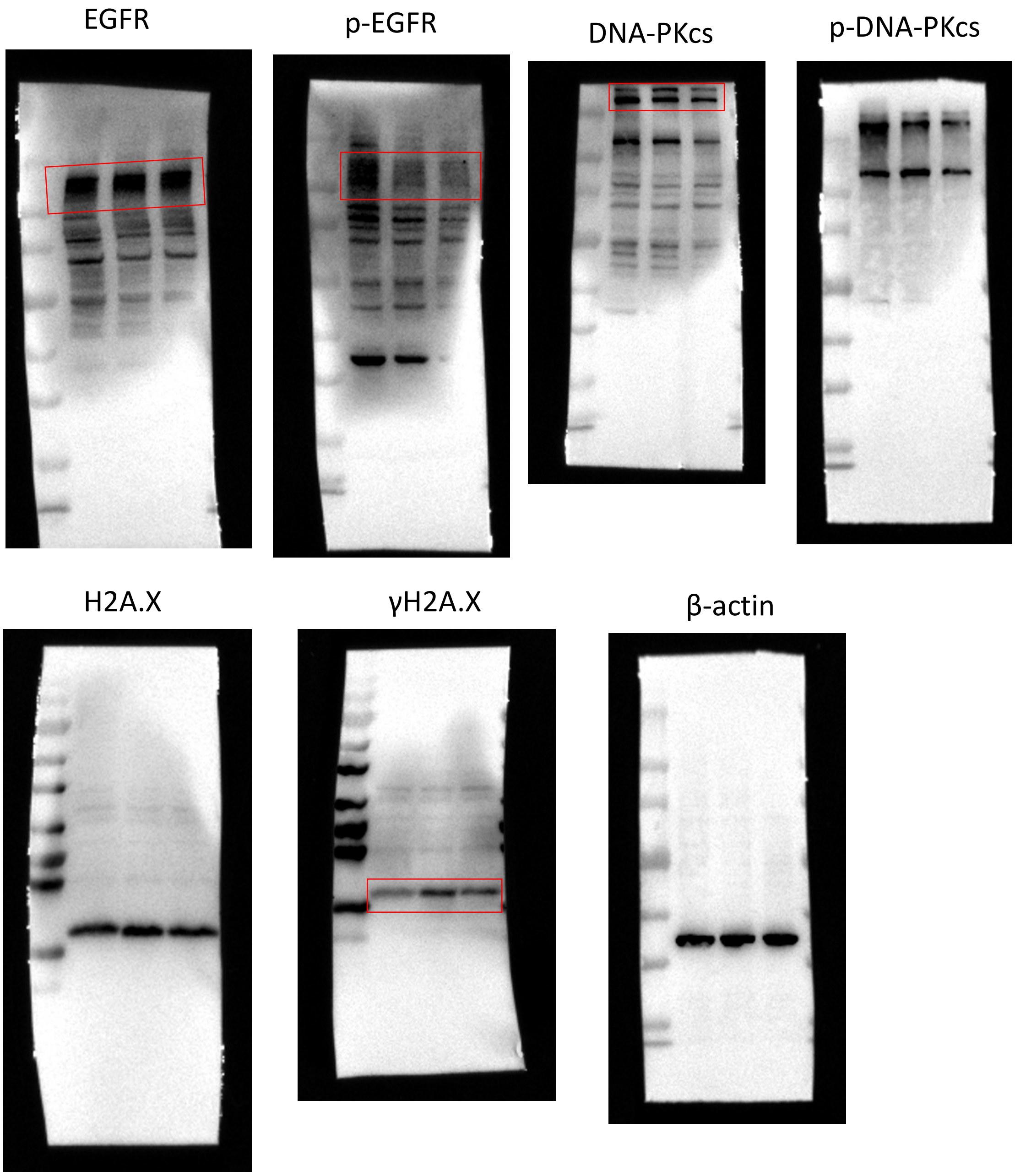
EGFR
p-EGFR
DNA-PKcs
p-DNA-PKcs
H2A.X
γH2A.X
β-actin

Fig6 D

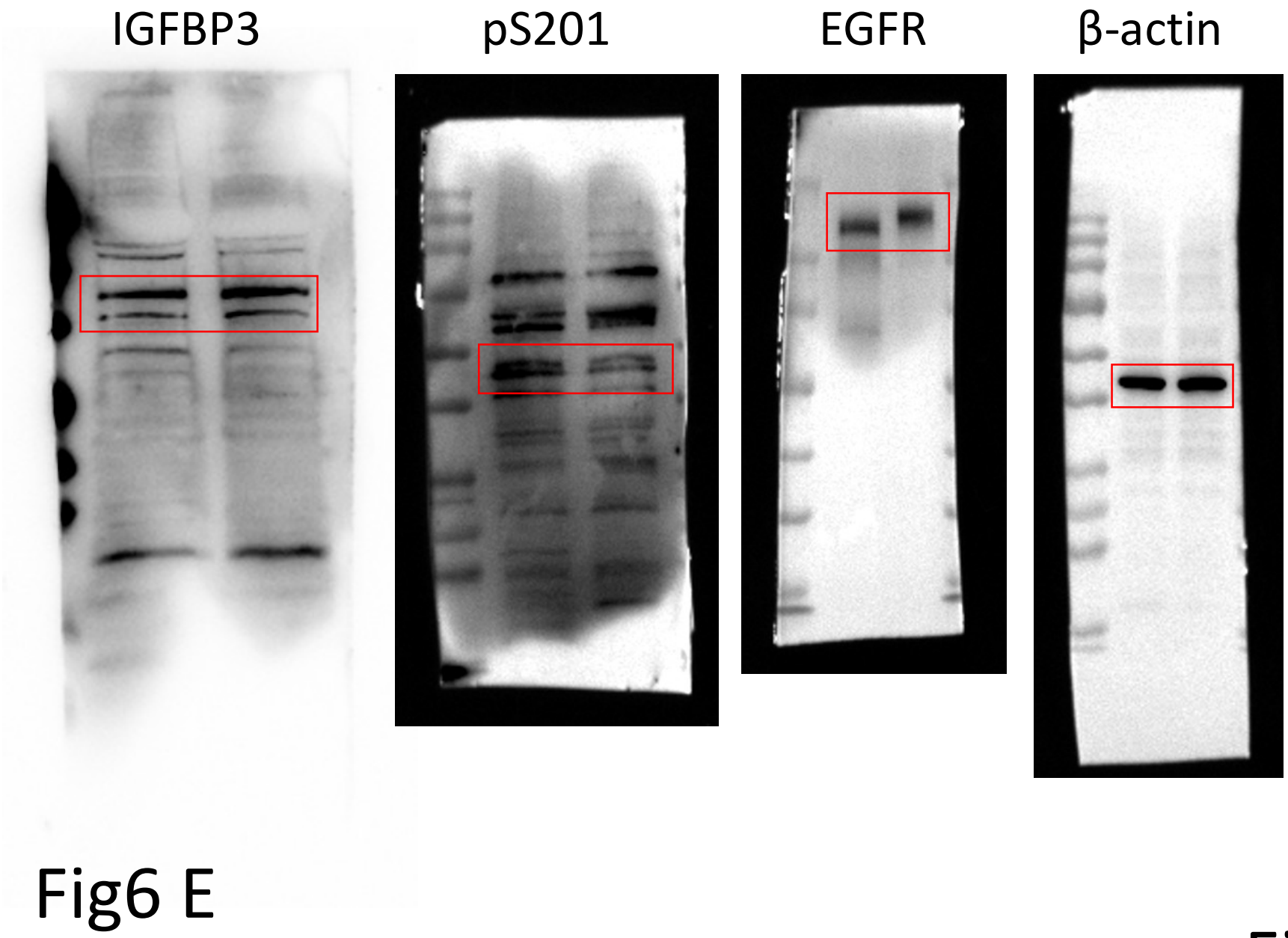

Fig6 E

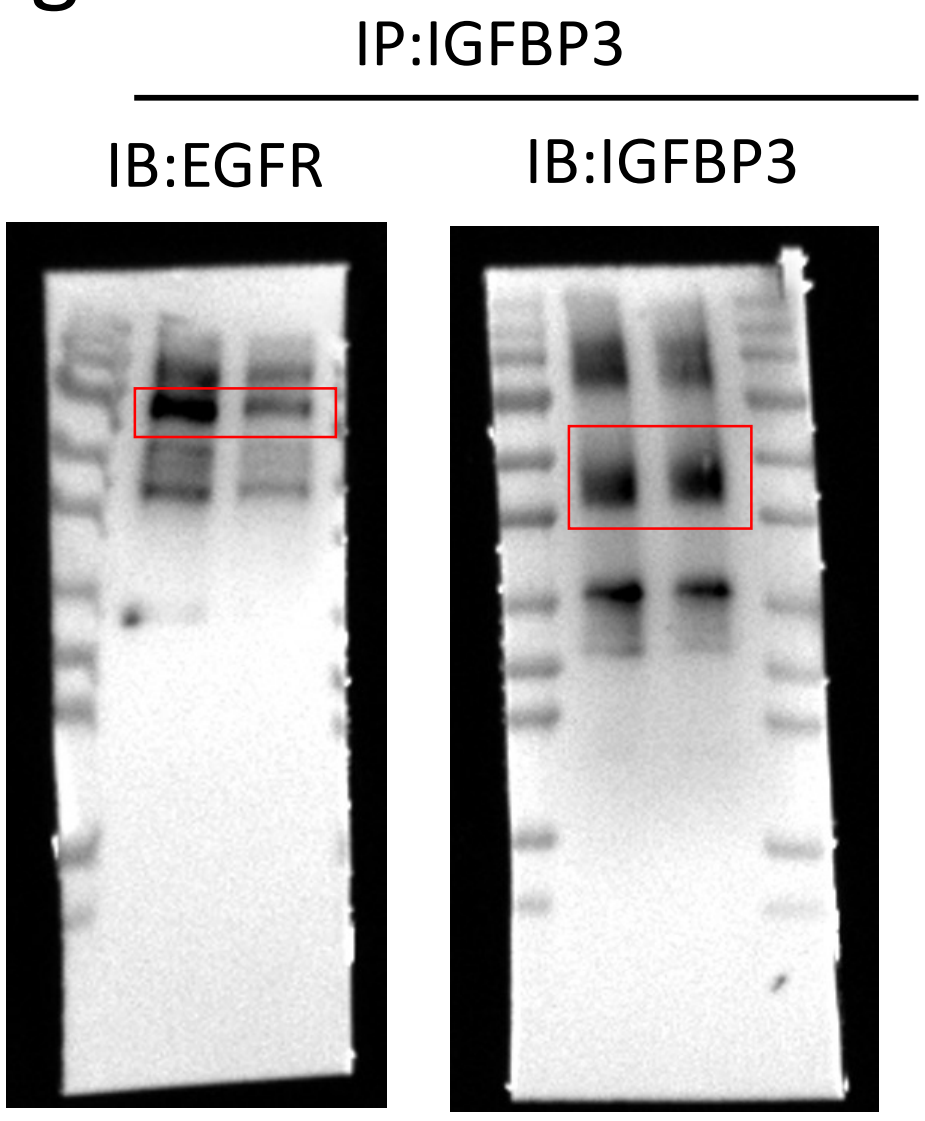

Fig6 E

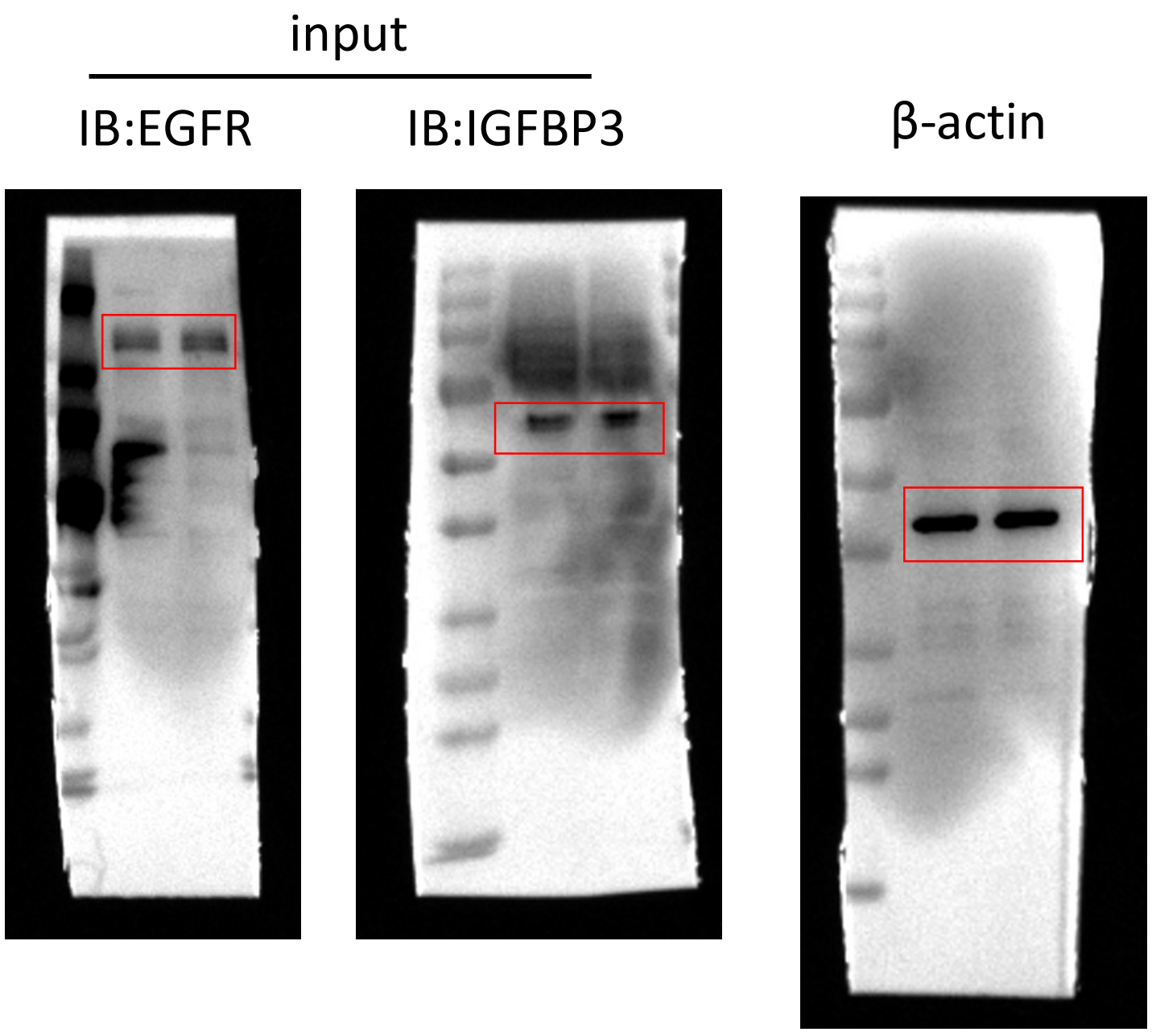

Fig6 H

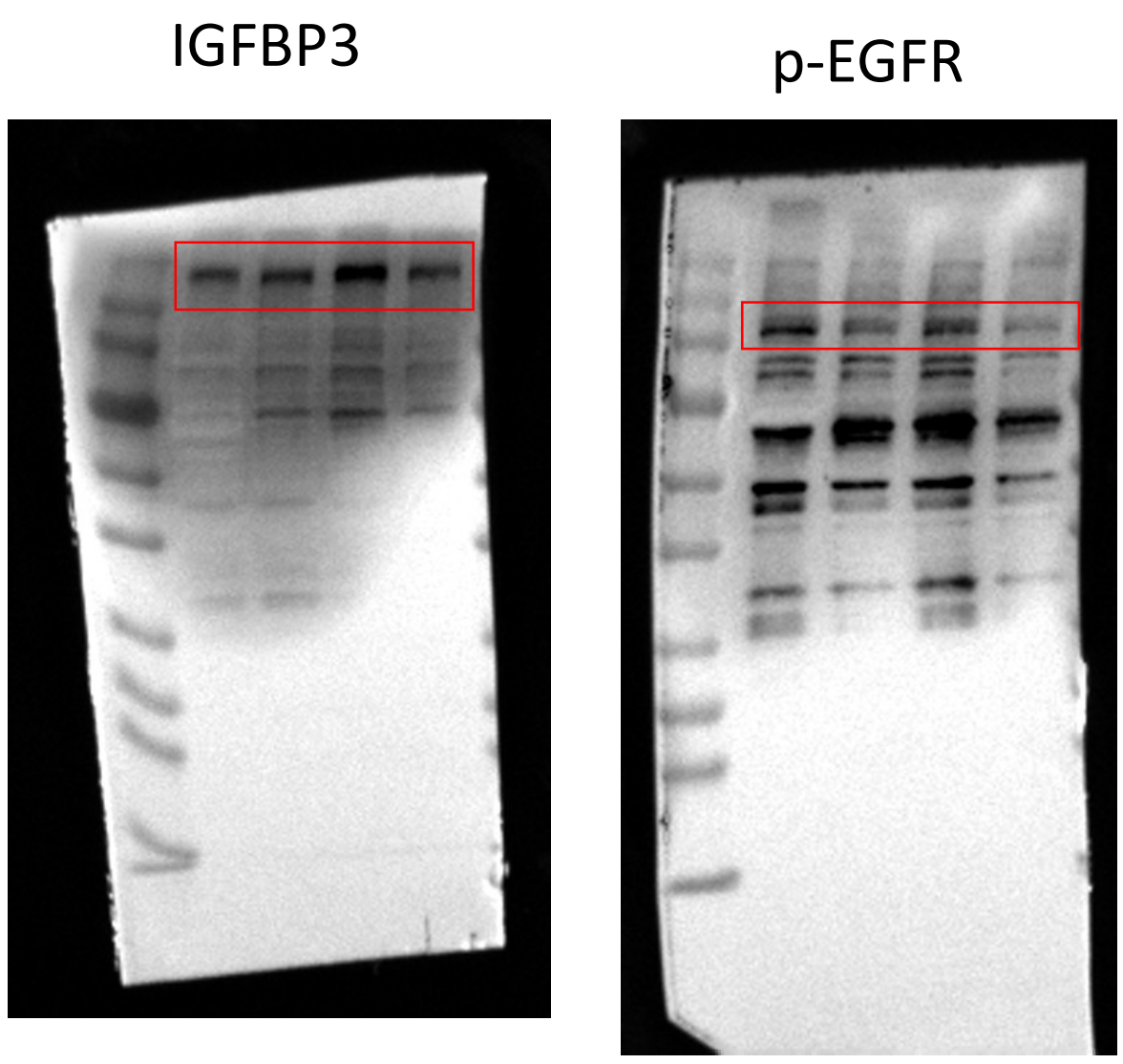

Fig6 H

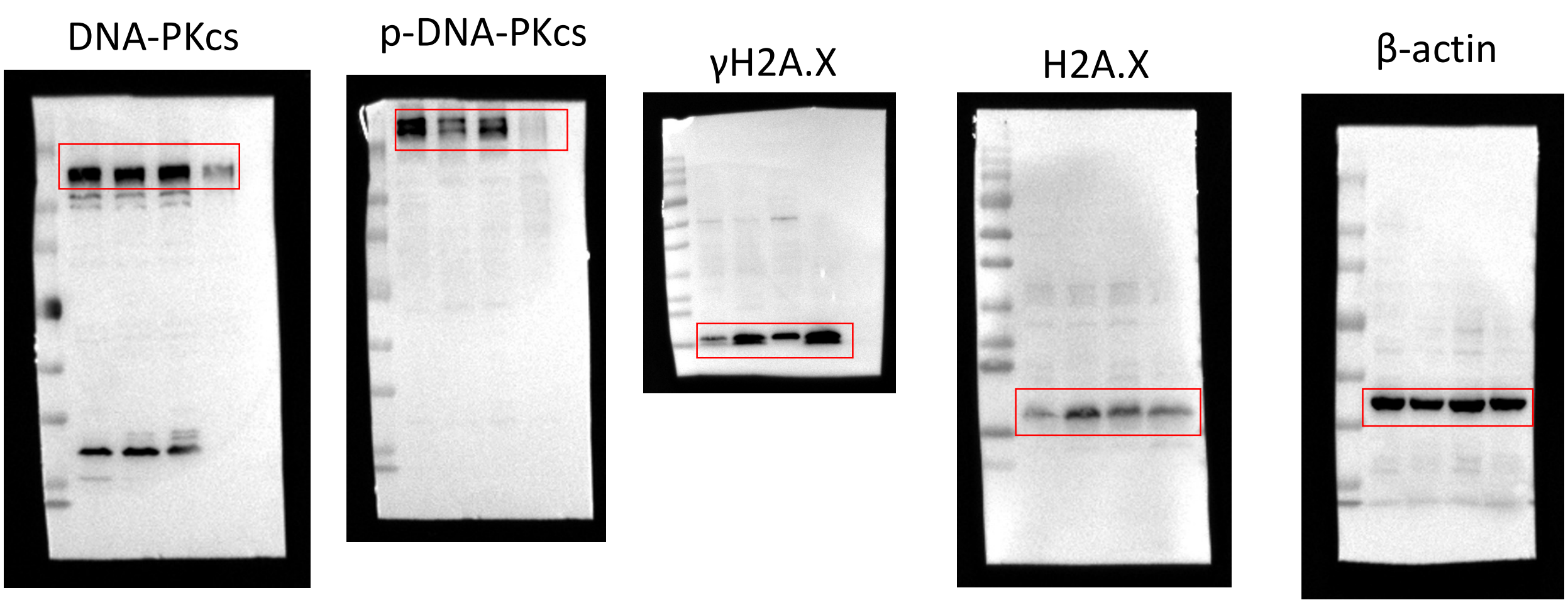

FigS1 D

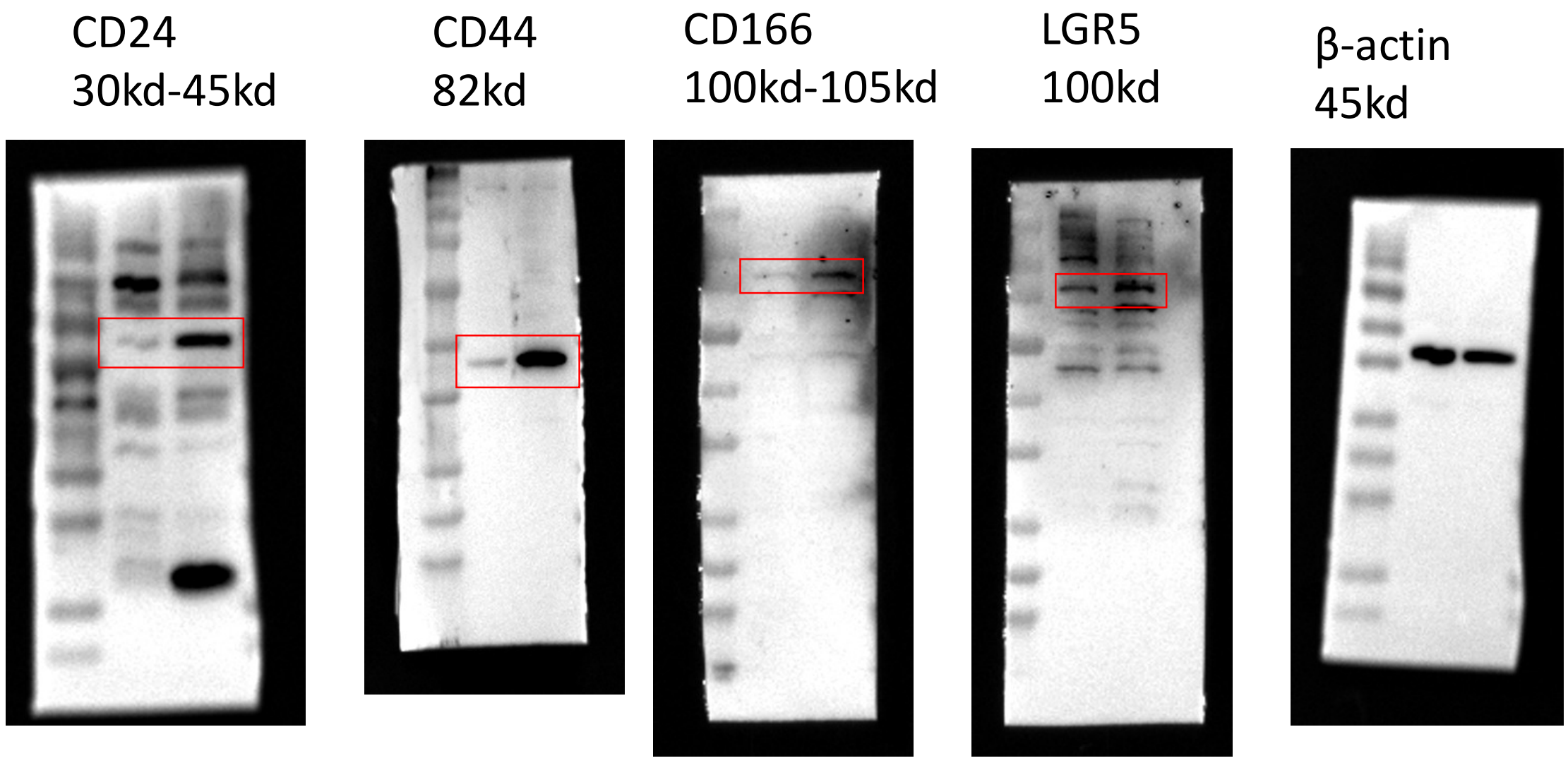

FigS1 H

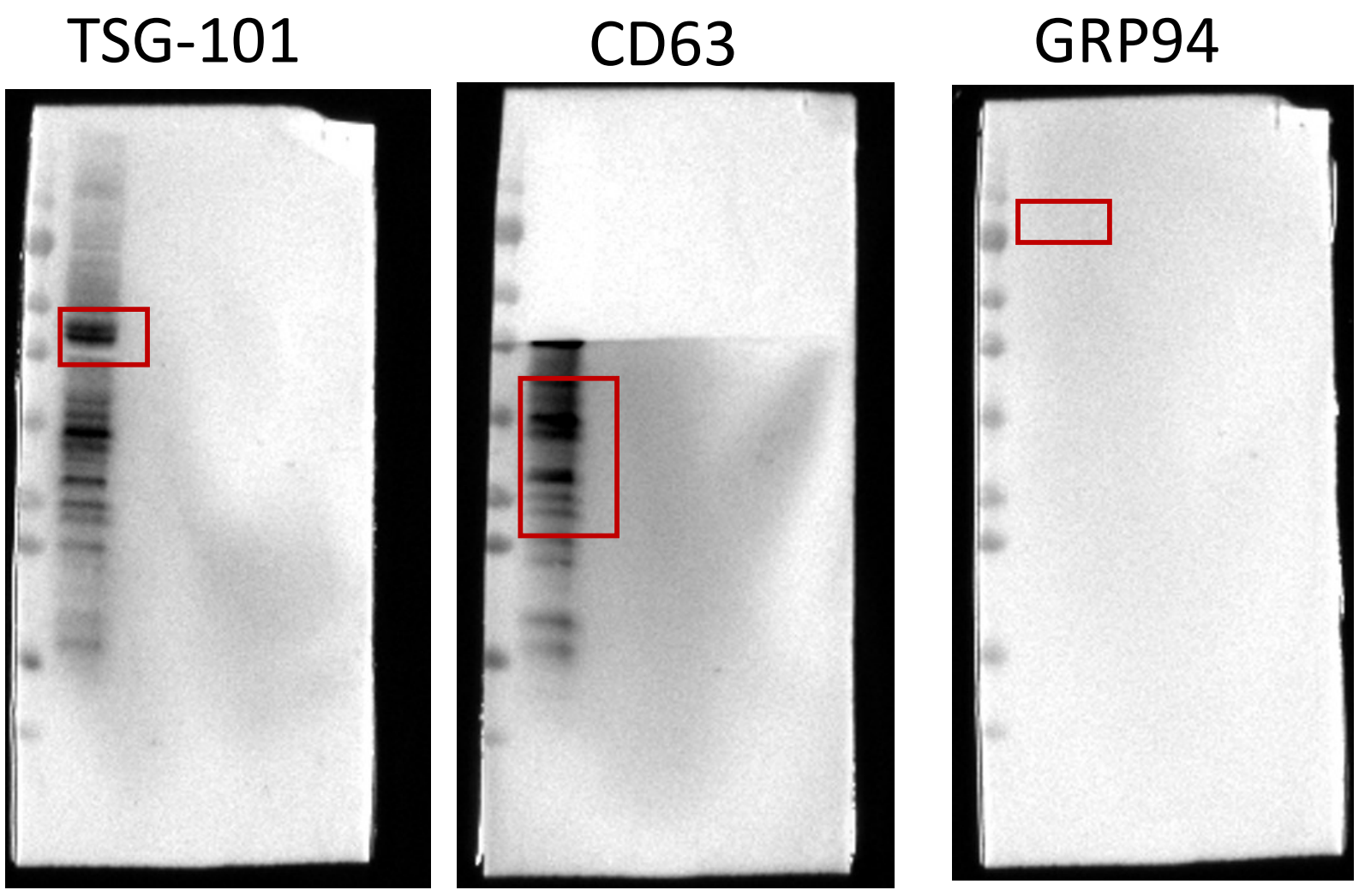

FigS2 G

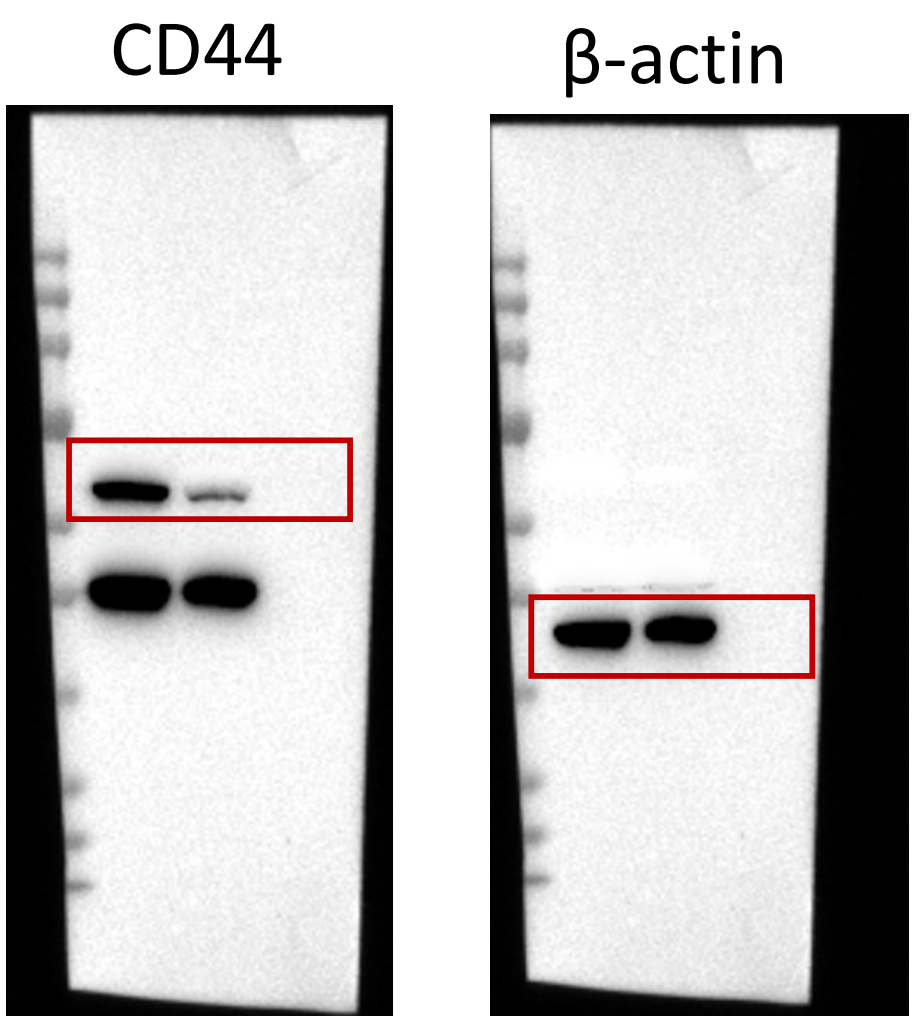

FigS3 A

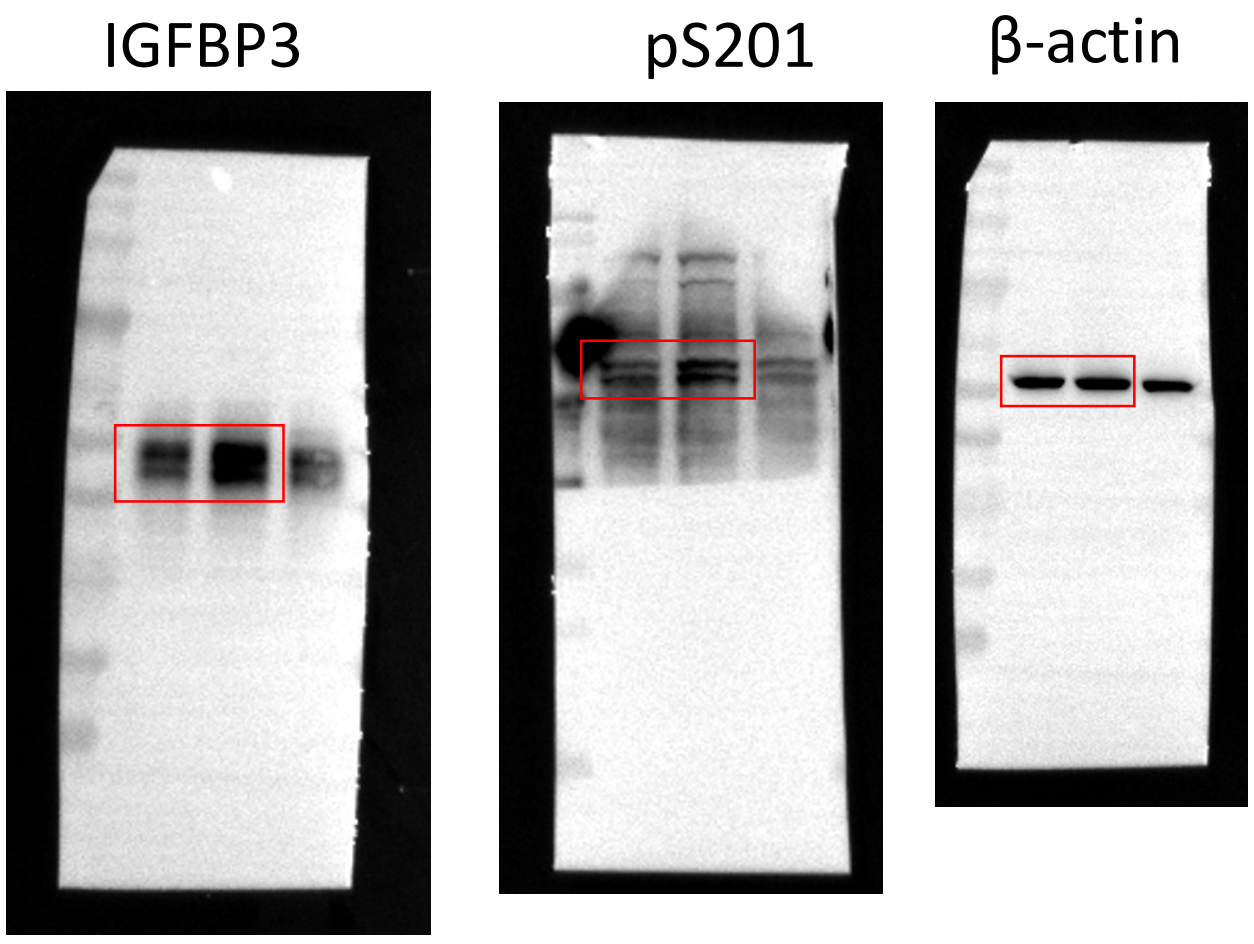

FigS4 C

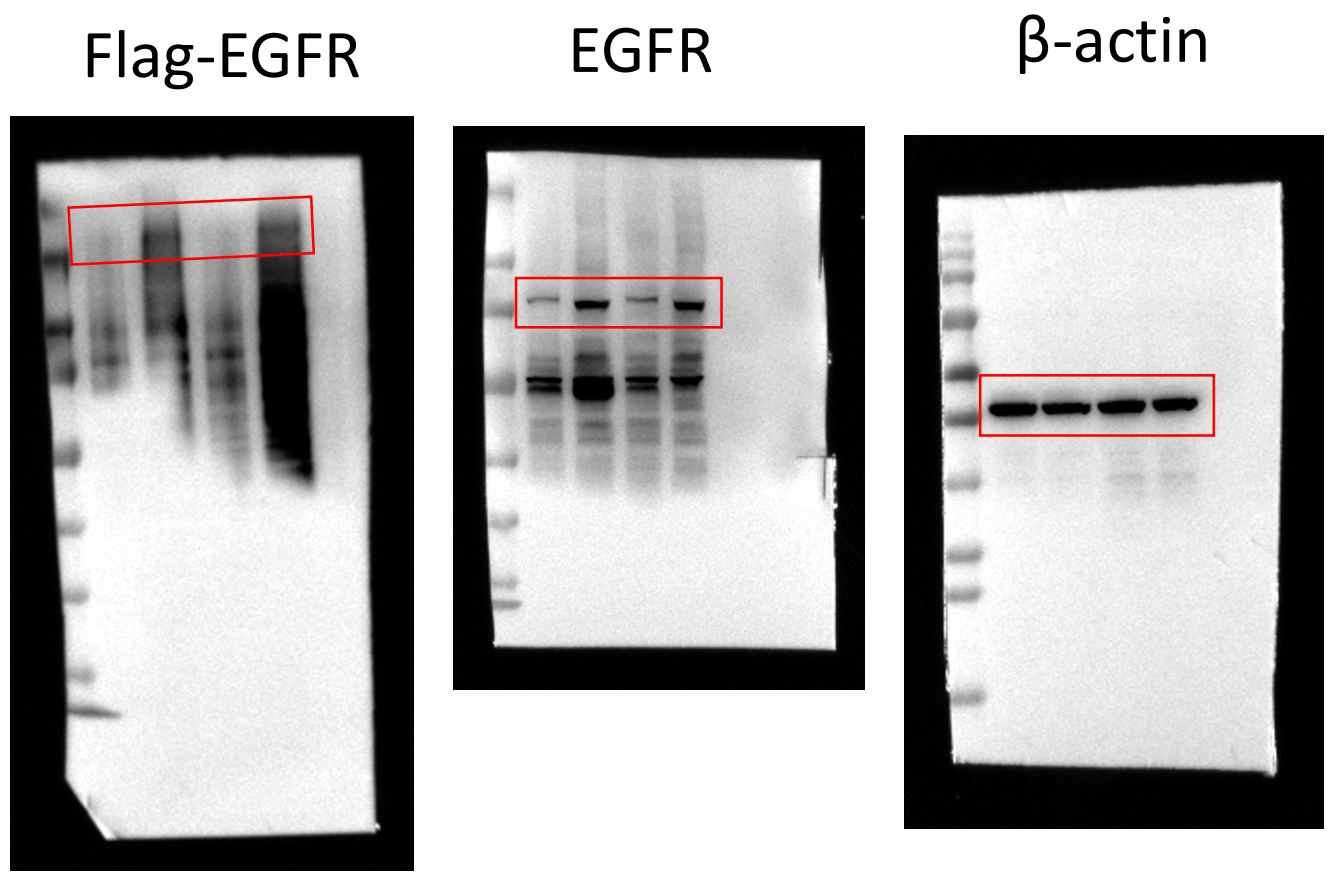

FigS5 A

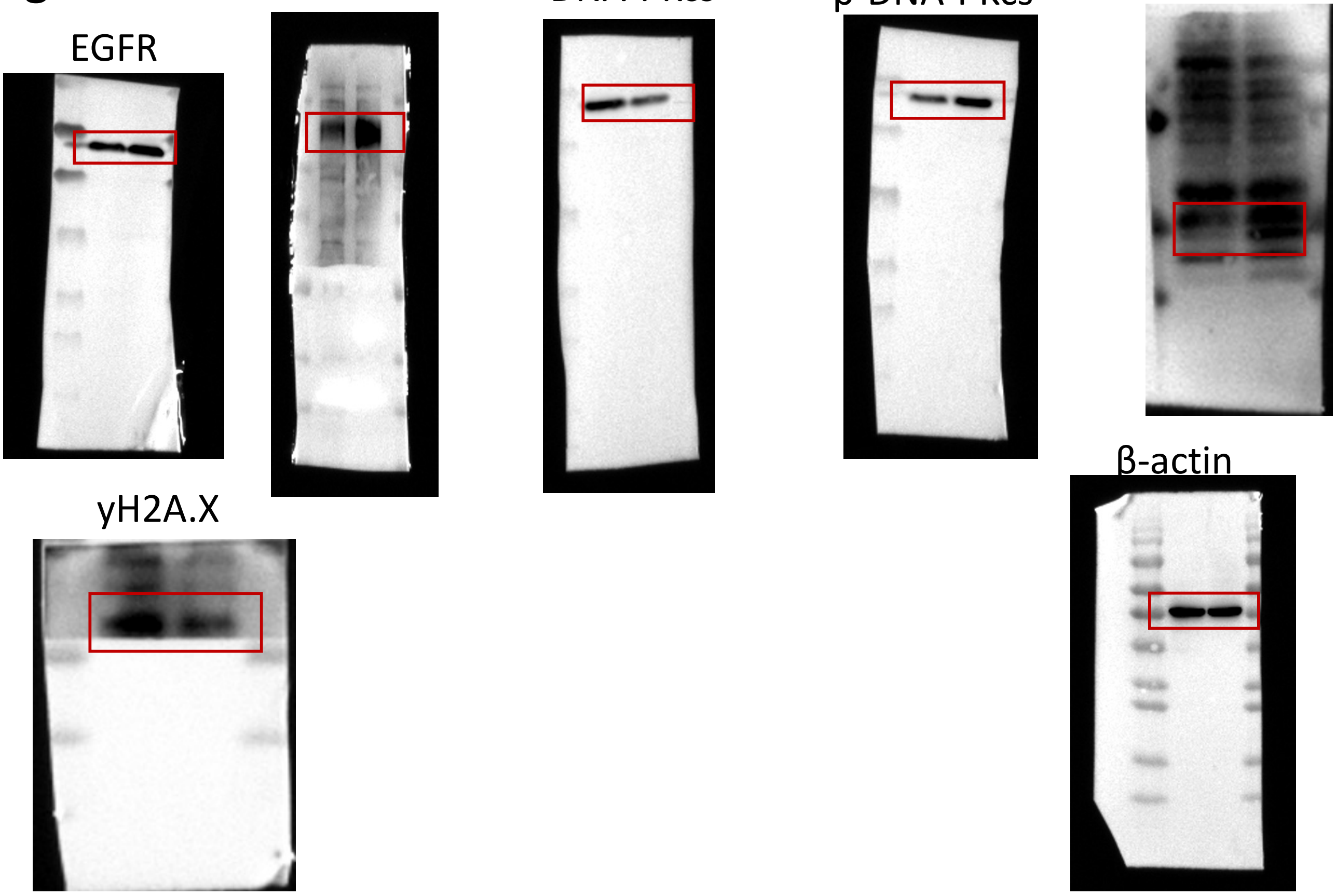

FigS5 B

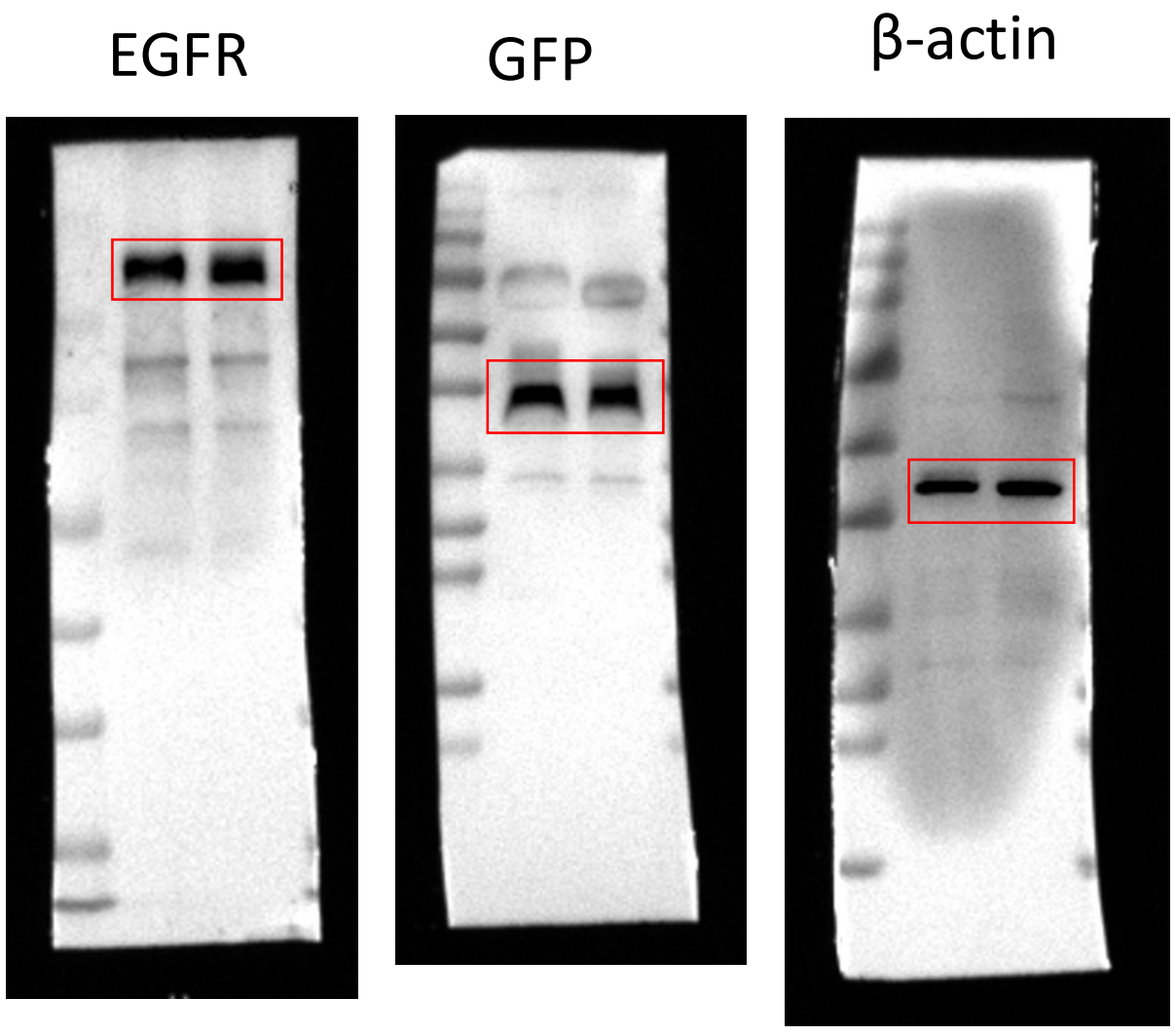

FigS5 C

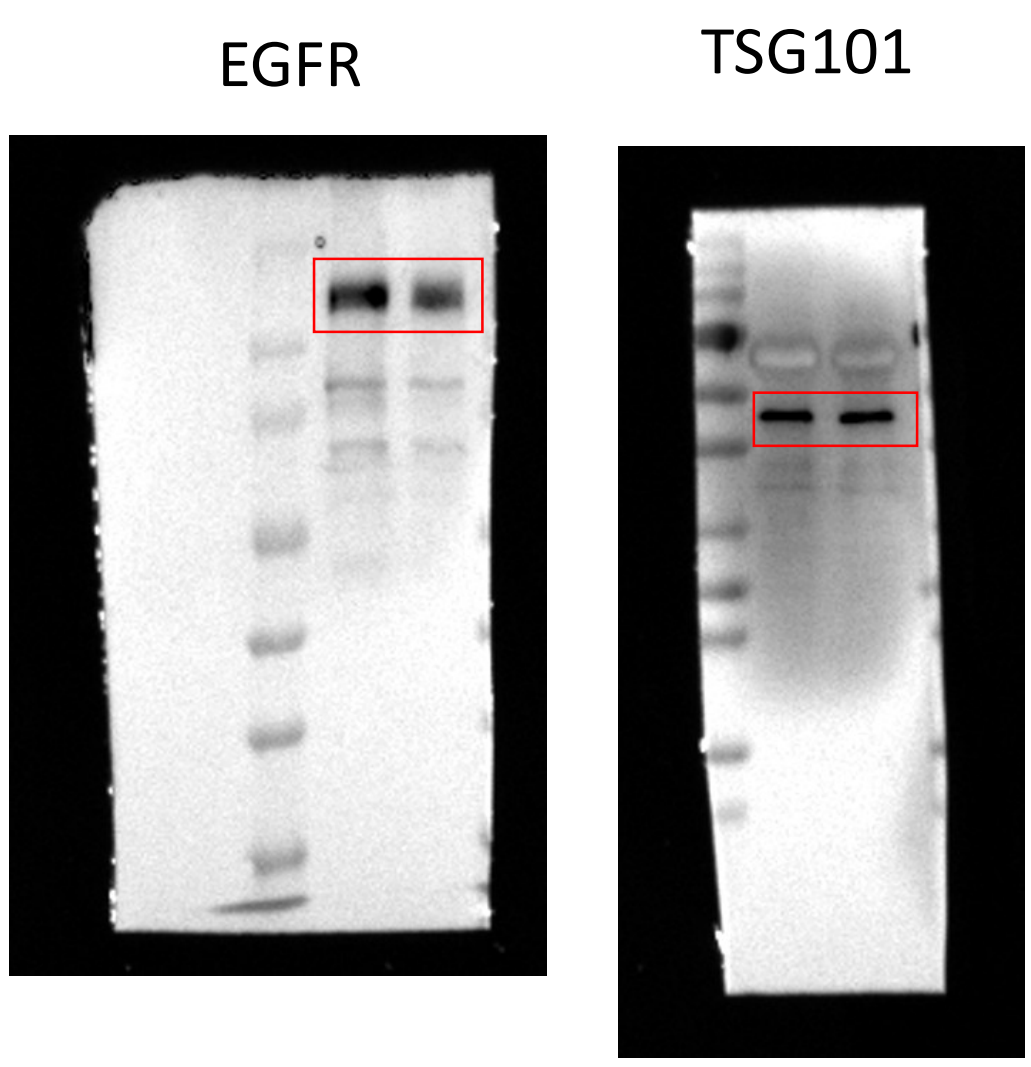

FigS5 D

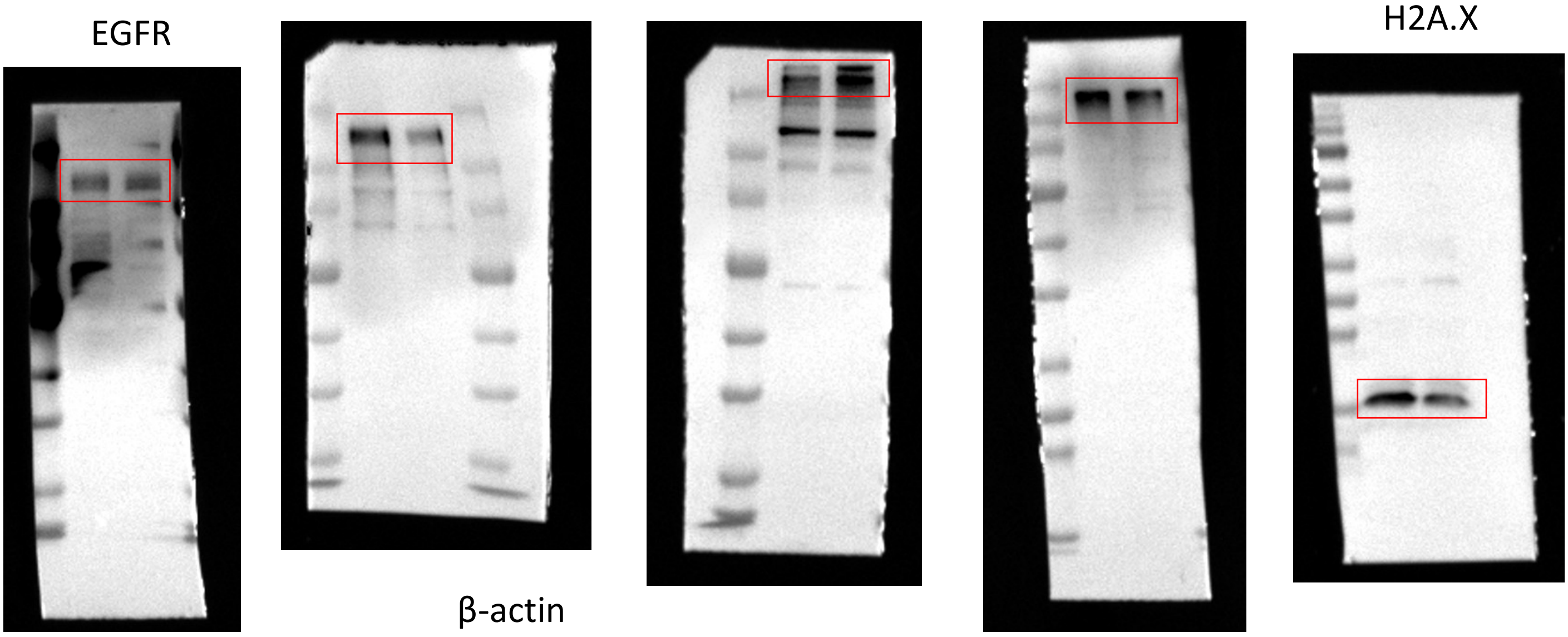

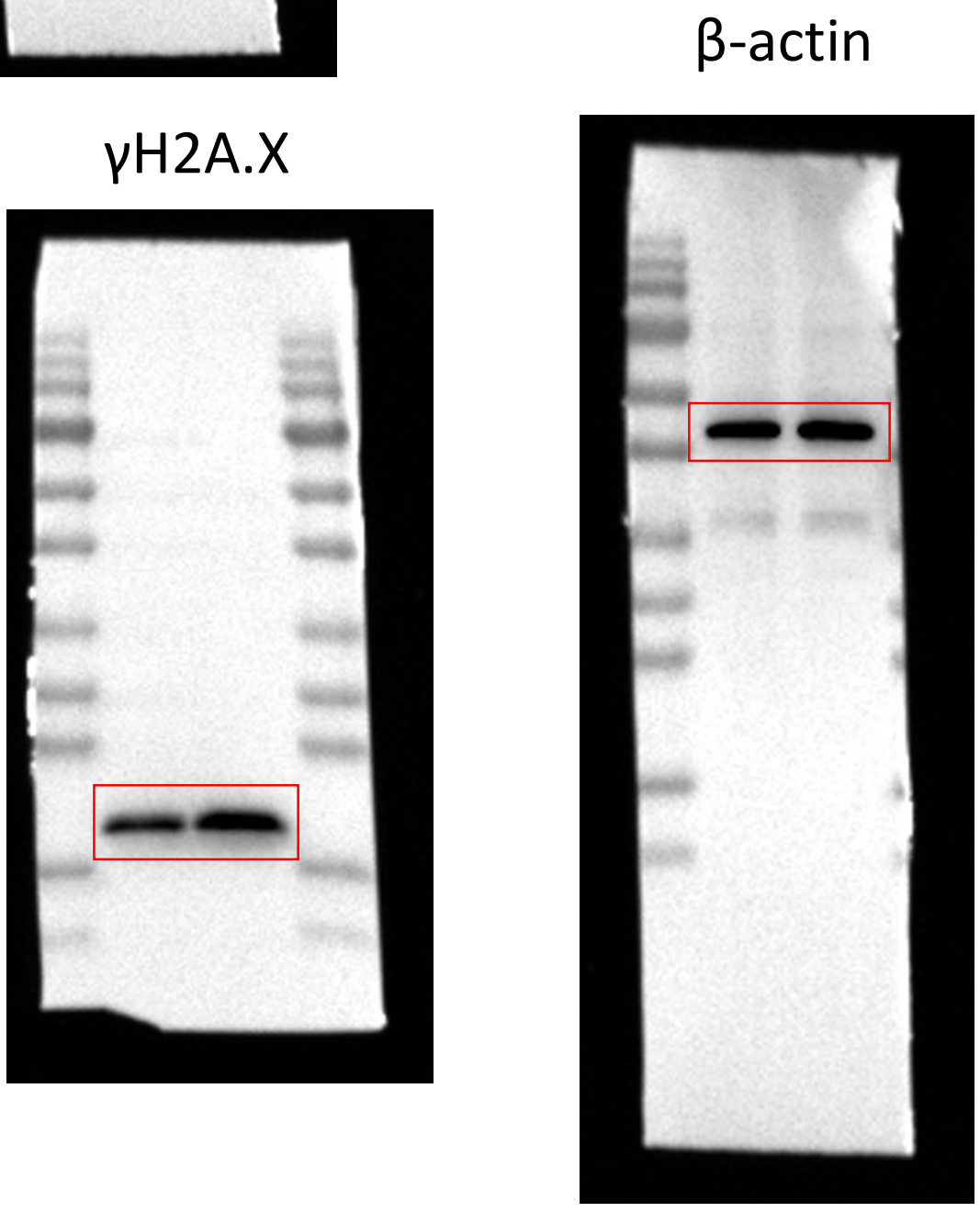

FigS5 E

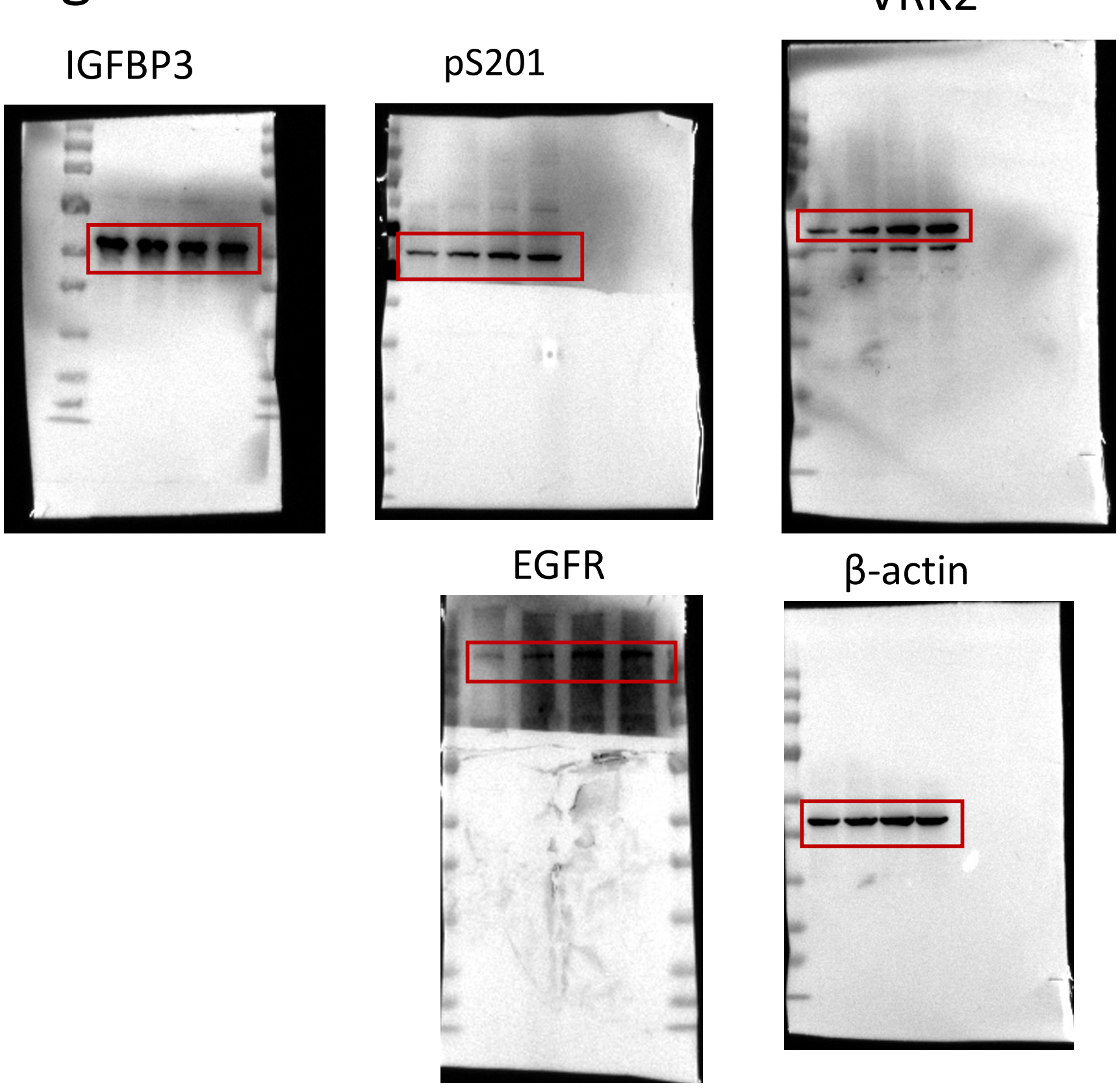

FigS5 F

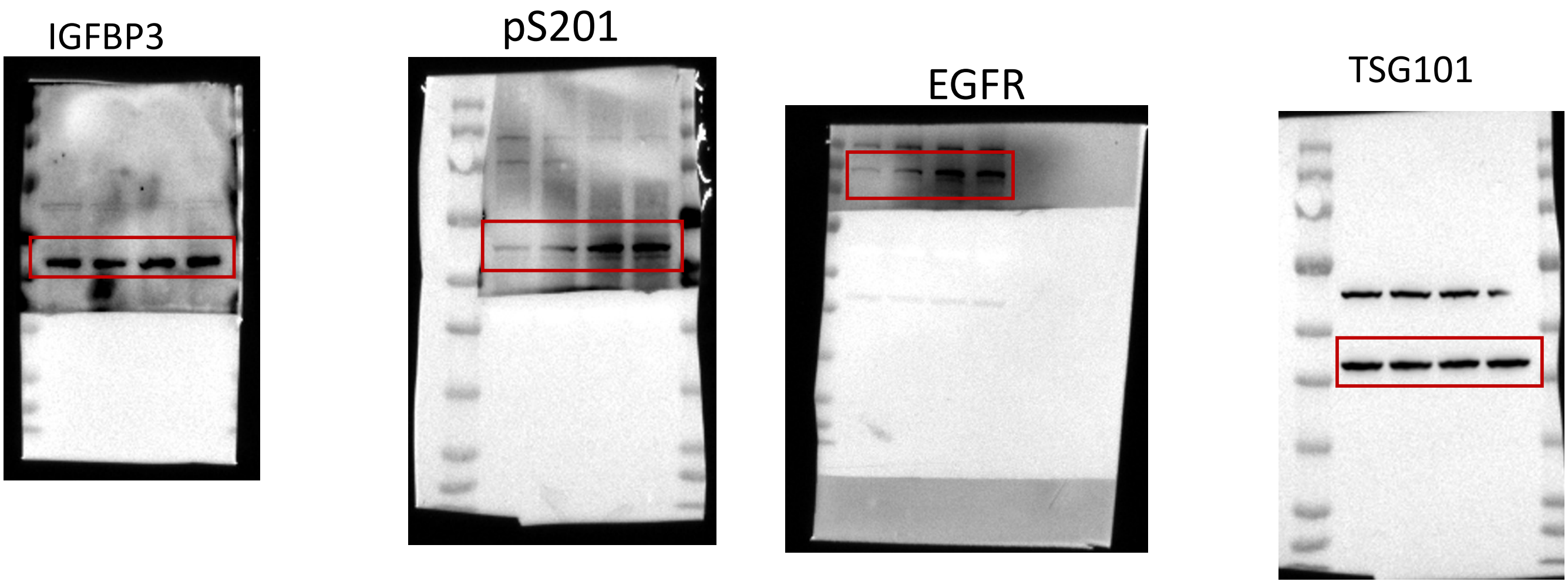

Supplement: Supplementary file 1 [file 41419_2026_9009_MOESM5_ESM.pdf]
