# Supplementary material for: EGFR-dependent delivery of phosphorylated IGFBP3 by cancer stem cells drives chemotherapy resistance in colorectal cancer
Source: Cell Death Dis. 2026 Jun 29;17(1):804. doi: 10.1038/s41419-026-09009-0 (PMC13578265; doi:10.1038/s41419-026-09009-0)
Supplement: Supplementary file 2 [file 41419_2026_9009_MOESM1_ESM.docx]

**Supplementary Materials**

Figure S1. Characterization of CCSCs and their sEVs.

(A) Schematic of CCSC enrichment under specific culture factor conditions.

(B) SW480-derived CCSCs were enriched in a low-adhesion plate using stem cell culture medium supplemented with growth factors, and sphere formation was observed. Scale bar: 100 μm.

(C) Cell cycle analysis of CCSCs and SW480 cells, with the proportions of each phase calculated.

(D,E) Western blot and flow cytometry analysis of surface-specific markers expressed on RKO-derived CCSCs: CD44, CD24, CD166, and LGR5.

(F) Transmission electron microscopy images of SW480-derived sEVs (SsEV) morphology. Scale bar: 100 nm.

(G) Nanoparticle tracking analysis of the sEVs particle size distribution.

(H) Western blot analysis of sEVs-positive markers TSG101 and CD63, and negative marker GRP94 expression. Data are presented as mean ± SD (n=3; **P < 0.01, ***P < 0.001).

Figure S2. CCSCs-derived sEVs contributed to chemotherapy resistance.

(A) PKH67 fluorescence staining of SW480 (SsEV) and their CSC-derived sEVs (CsEV), with fluorescence microscopy showing sEV uptake, and cell nuclei stained with DAPI. Scale bar: 50 μm.

(B-E) SW480 or RKO cells were co-cultured with sEVs at a concentration of 10^9^ particles/mL, followed by treatment with varying concentrations of oxaliplatin (0.1, 0.5, 1, 5, 20, 50 μM) or 5-FU (0.05, 1, 5, 10, 20, 50, 100 μM) for 48 hours. Cell viability was assessed by measuring the absorbance at 450 nm in the CCK-8 assay, and the IC50 values were calculated for each treatment.

(F) RKO cells were labeled with CoraLitePlus 488-conjugated CD44 antibody, and then separated into CD44-positive and CD44-negative RKO cells by flow cytometry.

(G) Western blot analysis of CD44 expression in CD44+ and CD44- RKO cells.

(H) sEVs derived from CD44+ and CD44- RKO cells were collected (CD44+ RsEV and CD44- RsEV). CD44+ RKO cell-derived CCSCs were enriched, followed by the collection of sEVs derived from CD44+ CCSCs (CD44+ CsEV). RKO cells were co-cultured with CD44- RsEV, CD44+ RsEV, CsEV or CD44+ CsEV at a concentration of 10^9^ particles/mL and treated with 5 μM oxaliplatin or 10 μM 5-FU for 48 hours. Cell viability was measured using a CCK-8 assay. Data are presented as mean ± SD (n=3; ns: not significant, ***P < 0.001).

Figure S3. sEVs derived from CCSCs enhance IGFBP3 and its phosphorylation levels in SW480 cells.

sEVs secreted by CCSCs and SW480 cells were added to SW480 cells and cultured for 48 hours. The protein expression of IGFBP3 and pS201 was then analyzed using Western blot (A) and immunofluorescence (B).

Figure S4. Overexpression of intracellular IGFBP3 enhances chemotherapy sensitivity in CRC cells.

(A) RKO cells were treated with 1 μg/mL IGF and 1 μg/mL IGFBP3, followed by 48-hour treatment with 5 μM oxaliplatin or 10 μM 5-FU. Cell viability was assessed by CCK-8 assay.

(B) Apoptosis rates were measured by Annexin V-PerCP and PI double staining after 48-hour treatment with 10 μM 5-FU in RKO and IGFBP3-overexpressing cell lines.

(C) Flag-tagged EGFR plasmid or control plasmid was transfected into RKO and IGFBP3-overexpressing cell lines, and Western blot analysis was performed to detect the expression of exogenous and endogenous EGFR. Data are presented as mean ± SD (n=3; ns: not significant, *P < 0.05, **P < 0.01, ***P < 0.001).

Figure S5. IGFBP3 S201 phosphorylation delivered by CsEVs promotes DNA damage repair in an EGFR-dependent manner.

(A) Western blotting was conducted to analyze the expression of DNA damage and repair-associated proteins in RKO cells treated with 10 μM 5-FU and 10⁹ particles/mL RsEVs or CsEVs for 48 hours.

(B) CCSCs were enriched from RKO cell lines stably expressing IGFBP3 (IGFBP3/CCSC) or S201A (S201A/CCSC), and EGFR expression in these CCSCs was analyzed by Western blot.

(C) sEVs were isolated from IGFBP3/CCSC (IGFBP3-CsEV) and S201A/CCSC (S201A-CsEV) cells, and EGFR levels in the sEVs were examined by Western blot.

(D) RKO cells were treated with 10⁹ particles/mL IGFBP3-CsEV or S201A-CsEV in combination with 10 μM 5-FU, and expression of DNA damage and repair-related proteins was detected by Western blot.

(E) Expression levels of IGFBP3, pS201, VRK2 and EGFR were analyzed by Western blot in CD44− RKO, CD44+ RKO, CCSCs and CD44+ CCSCs.

(F) sEVs were isolated from CD44− RKO, CD44+ RKO, CCSCs and CD44+ CCSCs, and the presence of IGFBP3, pS201, and EGFR in the sEVs was evaluated by Western blot.

(G) CCSCs were treated with 10 μM 5-FU, 20 μM D4476 alone, or in combination for 48 hours. Cell spheroids were digested with trypsin prior to counting to assess cell viability.

(H) Subcutaneous tumor implantation in mice using RKO cells expressing luciferase, followed by treatment with 10 mg/kg 5-FU, 20 mg/kg D4476, or 10 mg/kg 5-FU + 20 mg/kg D4476 for 10 days. Bioluminescence imaging was performed on representative mice's tumors.

Data are presented as mean ± SD (n=3; ***P < 0.001).

Figure S6. Expression of IGFBP3, EGFR, and VRK2 in human colorectal cancer tissues

(A) TCGA analysis showing the expression of IGFBP3 across various cancer types.

(B-D) GEPIA database demonstrating differential gene expression of IGFBP3, EGFR, and VRK2 in colon adenocarcinoma (COAD), rectal adenocarcinoma (READ), and normal tissues.

(E-G) Pearson correlation analysis of the expression correlation between IGFBP3 and VRK2, IGFBP3 and EGFR, and pS201 and VRK2 in cancer tissues.
