# Supplementary material for: EGFR-dependent delivery of phosphorylated IGFBP3 by cancer stem cells drives chemotherapy resistance in colorectal cancer
Source: Cell Death Dis. 2026 Jun 29;17(1):804. doi: 10.1038/s41419-026-09009-0 (PMC13578265; doi:10.1038/s41419-026-09009-0)
Supplement: Supplementary file 3 [file 41419_2026_9009_MOESM3_ESM.docx]

Supplementary Table 2. Putative phosphorylation sites of IGFBP3 and corresponding protein kinases predicted using the NetPhos 3.1 tool

| # Sequence # x Context Score Kinase Answer | | | |  |  |
| --- | --- | --- | --- | --- | --- |
|  | | |  |  |  |
| # Sequence 30 S RAGASSAGL 0.476 CaM-II . | | | |  |  |
| # Sequence 30 S RAGASSAGL 0.451 GSK3 . | | | |  |  |
| # Sequence 30 S RAGASSAGL 0.403 PKC . | | | |  |  |
| # Sequence 30 S RAGASSAGL 0.373 CKI . | | | |  |  |
| # Sequence 30 S RAGASSAGL 0.366 cdc2 . | | | |  |  |
| # Sequence 30 S RAGASSAGL 0.356 PKA . | | | |  |  |
| # Sequence 30 S RAGASSAGL 0.354 RSK . | | | |  |  |
| # Sequence 30 S RAGASSAGL 0.350 DNAPK . | | | |  |  |
| # Sequence 30 S RAGASSAGL 0.335 p38MAPK . | | | |  |  |
| # Sequence 30 S RAGASSAGL 0.285 PKG . | | | |  |  |
| # Sequence 30 S RAGASSAGL 0.267 ATM . | | | |  |  |
| # Sequence 30 S RAGASSAGL 0.258 CKII . | | | |  |  |
| # Sequence 30 S RAGASSAGL 0.192 cdk5 . | | | |  |  |
| # Sequence 30 S RAGASSAGL 0.081 PKB . | | | |  |  |
| # Sequence 30 S RAGASSAGL 0.025 unsp . | | | |  |  |
| # |  |  |  |  |  |
| # Sequence 31 S AGASSAGLG 0.672 unsp YES | | | |  |  |
| # Sequence 31 S AGASSAGLG 0.466 GSK3 . | | | |  |  |
| # Sequence 31 S AGASSAGLG 0.427 CKI . | | | |  |  |
| # Sequence 31 S AGASSAGLG 0.422 CaM-II . | | | |  |  |
| # Sequence 31 S AGASSAGLG 0.404 cdc2 . | | | |  |  |
| # Sequence 31 S AGASSAGLG 0.401 PKA . | | | |  |  |
| # Sequence 31 S AGASSAGLG 0.357 DNAPK . | | | |  |  |
| # Sequence 31 S AGASSAGLG 0.355 CKII . | | | |  |  |
| # Sequence 31 S AGASSAGLG 0.328 PKG . | | | |  |  |
| # Sequence 31 S AGASSAGLG 0.314 ATM . | | | |  |  |
| # Sequence 31 S AGASSAGLG 0.309 PKB . | | | |  |  |
| # Sequence 31 S AGASSAGLG 0.293 p38MAPK . | | | |  |  |
| # Sequence 31 S AGASSAGLG 0.278 RSK . | | | |  |  |
| # Sequence 31 S AGASSAGLG 0.209 cdk5 . | | | |  |  |
| # Sequence 31 S AGASSAGLG 0.123 PKC . | | | |  |  |
| # |  |  |  |  |  |
| # Sequence 76 S TCALSEGQP 0.539 CKI YES | | | |  |  |
| # Sequence 76 S TCALSEGQP 0.462 GSK3 . | | | |  |  |
| # Sequence 76 S TCALSEGQP 0.456 cdc2 . | | | |  |  |
| # Sequence 76 S TCALSEGQP 0.455 CaM-II . | | | |  |  |
| # Sequence 76 S TCALSEGQP 0.352 CKII . | | | |  |  |
| # Sequence 76 S TCALSEGQP 0.347 DNAPK . | | | |  |  |
| # Sequence 76 S TCALSEGQP 0.312 RSK . | | | |  |  |
| # Sequence 76 S TCALSEGQP 0.312 p38MAPK . | | | |  |  |
| # Sequence 76 S TCALSEGQP 0.297 ATM . | | | |  |  |
| # Sequence 76 S TCALSEGQP 0.282 unsp . | | | |  |  |
| # Sequence 76 S TCALSEGQP 0.236 PKG . | | | |  |  |
| # Sequence 76 S TCALSEGQP 0.216 PKC . | | | |  |  |
| # Sequence 76 S TCALSEGQP 0.197 cdk5 . | | | |  |  |
| # Sequence 76 S TCALSEGQP 0.191 PKA . | | | |  |  |
| # Sequence 76 S TCALSEGQP 0.091 PKB . | | | |  |  |
| # |  |  |  |  |  |
| # Sequence 90 S ERCGSGLRC 0.602 PKA YES | | | |  |  |
| # Sequence 90 S ERCGSGLRC 0.522 cdc2 YES | | | |  |  |
| # Sequence 90 S ERCGSGLRC 0.483 CaM-II . | | | |  |  |
| # Sequence 90 S ERCGSGLRC 0.482 PKC . | | | |  |  |
| # Sequence 90 S ERCGSGLRC 0.451 GSK3 . | | | |  |  |
| # Sequence 90 S ERCGSGLRC 0.404 RSK . | | | |  |  |
| # Sequence 90 S ERCGSGLRC 0.371 unsp . | | | |  |  |
| # Sequence 90 S ERCGSGLRC 0.371 p38MAPK . | | | |  |  |
| # Sequence 90 S ERCGSGLRC 0.362 CKI . | | | |  |  |
| # Sequence 90 S ERCGSGLRC 0.348 DNAPK . | | | |  |  |
| # Sequence 90 S ERCGSGLRC 0.296 PKG . | | | |  |  |
| # Sequence 90 S ERCGSGLRC 0.274 ATM . | | | |  |  |
| # Sequence 90 S ERCGSGLRC 0.274 CKII . | | | |  |  |
| # Sequence 90 S ERCGSGLRC 0.213 cdk5 . | | | |  |  |
| # Sequence 90 S ERCGSGLRC 0.184 PKB . | | | |  |  |
| # |  |  |  |  |  |
| # Sequence 97 S RCQPSPDEA 0.991 unsp YES | | | |  |  |
| # Sequence 97 S RCQPSPDEA 0.496 GSK3 . | | | |  |  |
| # Sequence 97 S RCQPSPDEA 0.495 CaM-II . | | | |  |  |
| # Sequence 97 S RCQPSPDEA 0.479 cdk5 . | | | |  |  |
| # Sequence 97 S RCQPSPDEA 0.455 p38MAPK . | | | |  |  |
| # Sequence 97 S RCQPSPDEA 0.429 cdc2 . | | | |  |  |
| # Sequence 97 S RCQPSPDEA 0.411 CKII . | | | |  |  |
| # Sequence 97 S RCQPSPDEA 0.369 CKI . | | | |  |  |
| # Sequence 97 S RCQPSPDEA 0.366 RSK . | | | |  |  |
| # Sequence 97 S RCQPSPDEA 0.354 DNAPK . | | | |  |  |
| # Sequence 97 S RCQPSPDEA 0.349 PKG . | | | |  |  |
| # Sequence 97 S RCQPSPDEA 0.284 ATM . | | | |  |  |
| # Sequence 97 S RCQPSPDEA 0.139 PKA . | | | |  |  |
| # Sequence 97 S RCQPSPDEA 0.085 PKB . | | | |  |  |
| # Sequence 97 S RCQPSPDEA 0.045 PKC . | | | |  |  |
| # |  |  |  |  |  |
| # Sequence 118 S CVNASAVSR 0.514 cdc2 YES | | | |  |  |
| # Sequence 118 S CVNASAVSR 0.453 CaM-II . | | | |  |  |
| # Sequence 118 S CVNASAVSR 0.434 GSK3 . | | | |  |  |
| # Sequence 118 S CVNASAVSR 0.375 p38MAPK . | | | |  |  |
| # Sequence 118 S CVNASAVSR 0.364 CKI . | | | |  |  |
| # Sequence 118 S CVNASAVSR 0.358 DNAPK . | | | |  |  |
| # Sequence 118 S CVNASAVSR 0.314 PKA . | | | |  |  |
| # Sequence 118 S CVNASAVSR 0.301 PKG . | | | |  |  |
| # Sequence 118 S CVNASAVSR 0.262 ATM . | | | |  |  |
| # Sequence 118 S CVNASAVSR 0.261 RSK . | | | |  |  |
| # Sequence 118 S CVNASAVSR 0.217 CKII . | | | |  |  |
| # Sequence 118 S CVNASAVSR 0.208 cdk5 . | | | |  |  |
| # Sequence 118 S CVNASAVSR 0.172 PKC . | | | |  |  |
| # Sequence 118 S CVNASAVSR 0.089 PKB . | | | |  |  |
| # Sequence 118 S CVNASAVSR 0.005 unsp . | | | |  |  |
| # |  |  |  |  |  |
| # Sequence 121 S ASAVSRLRA 0.543 cdc2 YES | | | |  |  |
| # Sequence 121 S ASAVSRLRA 0.469 CaM-II . | | | |  |  |
| # Sequence 121 S ASAVSRLRA 0.452 GSK3 . | | | |  |  |
| # Sequence 121 S ASAVSRLRA 0.394 unsp . | | | |  |  |
| # Sequence 121 S ASAVSRLRA 0.375 CKI . | | | |  |  |
| # Sequence 121 S ASAVSRLRA 0.338 DNAPK . | | | |  |  |
| # Sequence 121 S ASAVSRLRA 0.337 CKII . | | | |  |  |
| # Sequence 121 S ASAVSRLRA 0.326 PKG . | | | |  |  |
| # Sequence 121 S ASAVSRLRA 0.268 p38MAPK . | | | |  |  |
| # Sequence 121 S ASAVSRLRA 0.253 PKC . | | | |  |  |
| # Sequence 121 S ASAVSRLRA 0.249 ATM . | | | |  |  |
| # Sequence 121 S ASAVSRLRA 0.242 RSK . | | | |  |  |
| # Sequence 121 S ASAVSRLRA 0.217 cdk5 . | | | |  |  |
| # Sequence 121 S ASAVSRLRA 0.134 PKA . | | | |  |  |
| # Sequence 121 S ASAVSRLRA 0.078 PKB . | | | |  |  |
| # |  |  |  |  |  |
| # Sequence 138 S PGNASESEE 0.988 unsp YES | | | |  |  |
| # Sequence 138 S PGNASESEE 0.674 CKII YES | | | |  |  |
| # Sequence 138 S PGNASESEE 0.448 cdc2 . | | | |  |  |
| # Sequence 138 S PGNASESEE 0.437 CaM-II . | | | |  |  |
| # Sequence 138 S PGNASESEE 0.431 GSK3 . | | | |  |  |
| # Sequence 138 S PGNASESEE 0.384 DNAPK . | | | |  |  |
| # Sequence 138 S PGNASESEE 0.363 CKI . | | | |  |  |
| # Sequence 138 S PGNASESEE 0.336 RSK . | | | |  |  |
| # Sequence 138 S PGNASESEE 0.297 PKG . | | | |  |  |
| # Sequence 138 S PGNASESEE 0.276 ATM . | | | |  |  |
| # Sequence 138 S PGNASESEE 0.274 p38MAPK . | | | |  |  |
| # Sequence 138 S PGNASESEE 0.212 PKA . | | | |  |  |
| # Sequence 138 S PGNASESEE 0.161 cdk5 . | | | |  |  |
| # Sequence 138 S PGNASESEE 0.085 PKB . | | | |  |  |
| # Sequence 138 S PGNASESEE 0.071 PKC . | | | |  |  |
| # |  |  |  |  |  |
| # Sequence 140 S NASESEEDR 0.997 unsp YES | | | |  |  |
| # Sequence 140 S NASESEEDR 0.612 CKII YES | | | |  |  |
| # Sequence 140 S NASESEEDR 0.456 cdc2 . | | | |  |  |
| # Sequence 140 S NASESEEDR 0.429 GSK3 . | | | |  |  |
| # Sequence 140 S NASESEEDR 0.408 CKI . | | | |  |  |
| # Sequence 140 S NASESEEDR 0.398 CaM-II . | | | |  |  |
| # Sequence 140 S NASESEEDR 0.364 DNAPK . | | | |  |  |
| # Sequence 140 S NASESEEDR 0.317 ATM . | | | |  |  |
| # Sequence 140 S NASESEEDR 0.282 RSK . | | | |  |  |
| # Sequence 140 S NASESEEDR 0.270 p38MAPK . | | | |  |  |
| # Sequence 140 S NASESEEDR 0.226 PKG . | | | |  |  |
| # Sequence 140 S NASESEEDR 0.203 cdk5 . | | | |  |  |
| # Sequence 140 S NASESEEDR 0.105 PKC . | | | |  |  |
| # Sequence 140 S NASESEEDR 0.088 PKA . | | | |  |  |
| # Sequence 140 S NASESEEDR 0.073 PKB . | | | |  |  |
| # |  |  |  |  |  |
| # Sequence 145 S EEDRSAGSV 0.865 unsp YES | | | |  |  |
| # Sequence 145 S EEDRSAGSV 0.463 GSK3 . | | | |  |  |
| # Sequence 145 S EEDRSAGSV 0.427 CaM-II . | | | |  |  |
| # Sequence 145 S EEDRSAGSV 0.389 cdc2 . | | | |  |  |
| # Sequence 145 S EEDRSAGSV 0.384 CKII . | | | |  |  |
| # Sequence 145 S EEDRSAGSV 0.379 CKI . | | | |  |  |
| # Sequence 145 S EEDRSAGSV 0.369 DNAPK . | | | |  |  |
| # Sequence 145 S EEDRSAGSV 0.359 p38MAPK . | | | |  |  |
| # Sequence 145 S EEDRSAGSV 0.316 ATM . | | | |  |  |
| # Sequence 145 S EEDRSAGSV 0.280 PKG . | | | |  |  |
| # Sequence 145 S EEDRSAGSV 0.262 PKA . | | | |  |  |
| # Sequence 145 S EEDRSAGSV 0.229 cdk5 . | | | |  |  |
| # Sequence 145 S EEDRSAGSV 0.218 RSK . | | | |  |  |
| # Sequence 145 S EEDRSAGSV 0.096 PKB . | | | |  |  |
| # Sequence 145 S EEDRSAGSV 0.055 PKC . | | | |  |  |
| # |  |  |  |  |  |
| # Sequence 148 S RSAGSVESP 0.993 unsp YES | | | |  |  |
| # Sequence 148 S RSAGSVESP 0.748 PKC YES | | | |  |  |
| # Sequence 148 S RSAGSVESP 0.583 cdc2 YES | | | |  |  |
| # Sequence 148 S RSAGSVESP 0.474 CaM-II . | | | |  |  |
| # Sequence 148 S RSAGSVESP 0.462 GSK3 . | | | |  |  |
| # Sequence 148 S RSAGSVESP 0.414 CKI . | | | |  |  |
| # Sequence 148 S RSAGSVESP 0.407 ATM . | | | |  |  |
| # Sequence 148 S RSAGSVESP 0.368 DNAPK . | | | |  |  |
| # Sequence 148 S RSAGSVESP 0.349 p38MAPK . | | | |  |  |
| # Sequence 148 S RSAGSVESP 0.336 CKII . | | | |  |  |
| # Sequence 148 S RSAGSVESP 0.299 RSK . | | | |  |  |
| # Sequence 148 S RSAGSVESP 0.197 PKG . | | | |  |  |
| # Sequence 148 S RSAGSVESP 0.194 cdk5 . | | | |  |  |
| # Sequence 148 S RSAGSVESP 0.167 PKA . | | | |  |  |
| # Sequence 148 S RSAGSVESP 0.087 PKB . | | | |  |  |
| # |  |  |  |  |  |
| # Sequence 151 S GSVESPSVS 0.935 unsp YES | | | |  |  |
| # Sequence 151 S GSVESPSVS 0.510 cdk5 YES | | | |  |  |
| # Sequence 151 S GSVESPSVS 0.504 GSK3 YES | | | |  |  |
| # Sequence 151 S GSVESPSVS 0.494 CKI . | | | |  |  |
| # Sequence 151 S GSVESPSVS 0.488 p38MAPK . | | | |  |  |
| # Sequence 151 S GSVESPSVS 0.474 cdc2 . | | | |  |  |
| # Sequence 151 S GSVESPSVS 0.433 CaM-II . | | | |  |  |
| # Sequence 151 S GSVESPSVS 0.370 DNAPK . | | | |  |  |
| # Sequence 151 S GSVESPSVS 0.346 RSK . | | | |  |  |
| # Sequence 151 S GSVESPSVS 0.307 CKII . | | | |  |  |
| # Sequence 151 S GSVESPSVS 0.288 ATM . | | | |  |  |
| # Sequence 151 S GSVESPSVS 0.249 PKG . | | | |  |  |
| # Sequence 151 S GSVESPSVS 0.099 PKC . | | | |  |  |
| # Sequence 151 S GSVESPSVS 0.092 PKA . | | | |  |  |
| # Sequence 151 S GSVESPSVS 0.084 PKB . | | | |  |  |
| # |  |  |  |  |  |
| # Sequence 153 S VESPSVSST 0.985 unsp YES | | | |  |  |
| # Sequence 153 S VESPSVSST 0.487 cdc2 . | | | |  |  |
| # Sequence 153 S VESPSVSST 0.452 GSK3 . | | | |  |  |
| # Sequence 153 S VESPSVSST 0.446 CaM-II . | | | |  |  |
| # Sequence 153 S VESPSVSST 0.363 CKI . | | | |  |  |
| # Sequence 153 S VESPSVSST 0.356 DNAPK . | | | |  |  |
| # Sequence 153 S VESPSVSST 0.314 p38MAPK . | | | |  |  |
| # Sequence 153 S VESPSVSST 0.278 PKC . | | | |  |  |
| # Sequence 153 S VESPSVSST 0.272 ATM . | | | |  |  |
| # Sequence 153 S VESPSVSST 0.266 CKII . | | | |  |  |
| # Sequence 153 S VESPSVSST 0.257 RSK . | | | |  |  |
| # Sequence 153 S VESPSVSST 0.222 PKG . | | | |  |  |
| # Sequence 153 S VESPSVSST 0.182 cdk5 . | | | |  |  |
| # Sequence 153 S VESPSVSST 0.180 PKA . | | | |  |  |
| # Sequence 153 S VESPSVSST 0.086 PKB . | | | |  |  |
| # |  |  |  |  |  |
| # Sequence 155 S SPSVSSTHR 0.471 GSK3 . | | | |  |  |
| # Sequence 155 S SPSVSSTHR 0.448 CaM-II . | | | |  |  |
| # Sequence 155 S SPSVSSTHR 0.376 cdc2 . | | | |  |  |
| # Sequence 155 S SPSVSSTHR 0.375 CKI . | | | |  |  |
| # Sequence 155 S SPSVSSTHR 0.346 DNAPK . | | | |  |  |
| # Sequence 155 S SPSVSSTHR 0.323 cdk5 . | | | |  |  |
| # Sequence 155 S SPSVSSTHR 0.323 unsp . | | | |  |  |
| # Sequence 155 S SPSVSSTHR 0.321 CKII . | | | |  |  |
| # Sequence 155 S SPSVSSTHR 0.294 p38MAPK . | | | |  |  |
| # Sequence 155 S SPSVSSTHR 0.289 PKG . | | | |  |  |
| # Sequence 155 S SPSVSSTHR 0.285 RSK . | | | |  |  |
| # Sequence 155 S SPSVSSTHR 0.274 ATM . | | | |  |  |
| # Sequence 155 S SPSVSSTHR 0.197 PKA . | | | |  |  |
| # Sequence 155 S SPSVSSTHR 0.154 PKC . | | | |  |  |
| # Sequence 155 S SPSVSSTHR 0.084 PKB . | | | |  |  |
| # |  |  |  |  |  |
| # Sequence 156 S PSVSSTHRV 0.759 unsp YES | | | |  |  |
| # Sequence 156 S PSVSSTHRV 0.706 PKC YES | | | |  |  |
| # Sequence 156 S PSVSSTHRV 0.442 cdc2 . | | | |  |  |
| # Sequence 156 S PSVSSTHRV 0.433 CaM-II . | | | |  |  |
| # Sequence 156 S PSVSSTHRV 0.430 GSK3 . | | | |  |  |
| # Sequence 156 S PSVSSTHRV 0.371 CKI . | | | |  |  |
| # Sequence 156 S PSVSSTHRV 0.350 DNAPK . | | | |  |  |
| # Sequence 156 S PSVSSTHRV 0.328 CKII . | | | |  |  |
| # Sequence 156 S PSVSSTHRV 0.301 ATM . | | | |  |  |
| # Sequence 156 S PSVSSTHRV 0.295 cdk5 . | | | |  |  |
| # Sequence 156 S PSVSSTHRV 0.290 RSK . | | | |  |  |
| # Sequence 156 S PSVSSTHRV 0.262 p38MAPK . | | | |  |  |
| # Sequence 156 S PSVSSTHRV 0.244 PKG . | | | |  |  |
| # Sequence 156 S PSVSSTHRV 0.115 PKA . | | | |  |  |
| # Sequence 156 S PSVSSTHRV 0.085 PKB . | | | |  |  |
| # |  |  |  |  |  |
| # Sequence 161 S THRVSDPKF 0.958 unsp YES | | | |  |  |
| # Sequence 161 S THRVSDPKF 0.506 cdc2 YES | | | |  |  |
| # Sequence 161 S THRVSDPKF 0.447 PKC . | | | |  |  |
| # Sequence 161 S THRVSDPKF 0.445 GSK3 . | | | |  |  |
| # Sequence 161 S THRVSDPKF 0.441 CaM-II . | | | |  |  |
| # Sequence 161 S THRVSDPKF 0.411 PKG . | | | |  |  |
| # Sequence 161 S THRVSDPKF 0.364 CKI . | | | |  |  |
| # Sequence 161 S THRVSDPKF 0.356 PKA . | | | |  |  |
| # Sequence 161 S THRVSDPKF 0.354 DNAPK . | | | |  |  |
| # Sequence 161 S THRVSDPKF 0.326 CKII . | | | |  |  |
| # Sequence 161 S THRVSDPKF 0.307 RSK . | | | |  |  |
| # Sequence 161 S THRVSDPKF 0.274 ATM . | | | |  |  |
| # Sequence 161 S THRVSDPKF 0.255 cdk5 . | | | |  |  |
| # Sequence 161 S THRVSDPKF 0.242 p38MAPK . | | | |  |  |
| # Sequence 161 S THRVSDPKF 0.088 PKB . | | | |  |  |
| # |  |  |  |  |  |
| # Sequence 170 S HPLHSKIII 0.578 PKC YES | | | |  |  |
| # Sequence 170 S HPLHSKIII 0.449 CaM-II . | | | |  |  |
| # Sequence 170 S HPLHSKIII 0.447 GSK3 . | | | |  |  |
| # Sequence 170 S HPLHSKIII 0.405 CKI . | | | |  |  |
| # Sequence 170 S HPLHSKIII 0.393 PKG . | | | |  |  |
| # Sequence 170 S HPLHSKIII 0.367 cdc2 . | | | |  |  |
| # Sequence 170 S HPLHSKIII 0.345 ATM . | | | |  |  |
| # Sequence 170 S HPLHSKIII 0.340 DNAPK . | | | |  |  |
| # Sequence 170 S HPLHSKIII 0.337 CKII . | | | |  |  |
| # Sequence 170 S HPLHSKIII 0.300 RSK . | | | |  |  |
| # Sequence 170 S HPLHSKIII 0.291 p38MAPK . | | | |  |  |
| # Sequence 170 S HPLHSKIII 0.233 PKA . | | | |  |  |
| # Sequence 170 S HPLHSKIII 0.154 cdk5 . | | | |  |  |
| # Sequence 170 S HPLHSKIII 0.080 PKB . | | | |  |  |
| # Sequence 170 S HPLHSKIII 0.054 unsp . | | | |  |  |
| # |  |  |  |  |  |
| # Sequence 183 S HAKDSQRYK 0.854 unsp YES | | | |  |  |
| # Sequence 183 S HAKDSQRYK 0.842 PKC YES | | | |  |  |
| # Sequence 183 S HAKDSQRYK 0.521 ATM YES | | | |  |  |
| # Sequence 183 S HAKDSQRYK 0.448 GSK3 . | | | |  |  |
| # Sequence 183 S HAKDSQRYK 0.427 CaM-II . | | | |  |  |
| # Sequence 183 S HAKDSQRYK 0.421 DNAPK . | | | |  |  |
| # Sequence 183 S HAKDSQRYK 0.399 cdc2 . | | | |  |  |
| # Sequence 183 S HAKDSQRYK 0.367 CKI . | | | |  |  |
| # Sequence 183 S HAKDSQRYK 0.326 p38MAPK . | | | |  |  |
| # Sequence 183 S HAKDSQRYK 0.320 PKG . | | | |  |  |
| # Sequence 183 S HAKDSQRYK 0.314 RSK . | | | |  |  |
| # Sequence 183 S HAKDSQRYK 0.314 CKII . | | | |  |  |
| # Sequence 183 S HAKDSQRYK 0.177 cdk5 . | | | |  |  |
| # Sequence 183 S HAKDSQRYK 0.136 PKA . | | | |  |  |
| # Sequence 183 S HAKDSQRYK 0.080 PKB . | | | |  |  |
| # |  |  |  |  |  |
| # Sequence 192 S VDYESQSTD 0.839 unsp YES | | | |  |  |
| # Sequence 192 S VDYESQSTD 0.624 DNAPK YES | | | |  |  |
| # Sequence 192 S VDYESQSTD 0.502 CKII YES | | | |  |  |
| # Sequence 192 S VDYESQSTD 0.467 ATM . | | | |  |  |
| # Sequence 192 S VDYESQSTD 0.460 cdc2 . | | | |  |  |
| # Sequence 192 S VDYESQSTD 0.444 GSK3 . | | | |  |  |
| # Sequence 192 S VDYESQSTD 0.443 CKI . | | | |  |  |
| # Sequence 192 S VDYESQSTD 0.440 CaM-II . | | | |  |  |
| # Sequence 192 S VDYESQSTD 0.298 p38MAPK . | | | |  |  |
| # Sequence 192 S VDYESQSTD 0.272 PKG . | | | |  |  |
| # Sequence 192 S VDYESQSTD 0.269 RSK . | | | |  |  |
| # Sequence 192 S VDYESQSTD 0.256 PKA . | | | |  |  |
| # Sequence 192 S VDYESQSTD 0.151 PKC . | | | |  |  |
| # Sequence 192 S VDYESQSTD 0.151 cdk5 . | | | |  |  |
| # Sequence 192 S VDYESQSTD 0.083 PKB . | | | |  |  |
| # |  |  |  |  |  |
| # Sequence 194 S YESQSTDTQ 0.561 CKII YES | | | |  |  |
| # Sequence 194 S YESQSTDTQ 0.460 cdc2 . | | | |  |  |
| # Sequence 194 S YESQSTDTQ 0.438 CaM-II . | | | |  |  |
| # Sequence 194 S YESQSTDTQ 0.433 GSK3 . | | | |  |  |
| # Sequence 194 S YESQSTDTQ 0.368 CKI . | | | |  |  |
| # Sequence 194 S YESQSTDTQ 0.358 DNAPK . | | | |  |  |
| # Sequence 194 S YESQSTDTQ 0.319 p38MAPK . | | | |  |  |
| # Sequence 194 S YESQSTDTQ 0.317 RSK . | | | |  |  |
| # Sequence 194 S YESQSTDTQ 0.300 ATM . | | | |  |  |
| # Sequence 194 S YESQSTDTQ 0.243 PKG . | | | |  |  |
| # Sequence 194 S YESQSTDTQ 0.163 unsp . | | | |  |  |
| # Sequence 194 S YESQSTDTQ 0.160 cdk5 . | | | |  |  |
| # Sequence 194 S YESQSTDTQ 0.147 PKA . | | | |  |  |
| # Sequence 194 S YESQSTDTQ 0.136 PKC . | | | |  |  |
| # Sequence 194 S YESQSTDTQ 0.096 PKB . | | | |  |  |
| # |  |  |  |  |  |
| # Sequence 201 S TQNFSSESK 0.955 unsp YES | | | |  |  |
| # Sequence 201 S TQNFSSESK 0.550 CKI YES | | | |  |  |
| # Sequence 201 S TQNFSSESK 0.466 CKII . | | | |  |  |
| # Sequence 201 S TQNFSSESK 0.447 GSK3 . | | | |  |  |
| # Sequence 201 S TQNFSSESK 0.436 CaM-II . | | | |  |  |
| # Sequence 201 S TQNFSSESK 0.394 cdc2 . | | | |  |  |
| # Sequence 201 S TQNFSSESK 0.366 DNAPK . | | | |  |  |
| # Sequence 201 S TQNFSSESK 0.361 ATM . | | | |  |  |
| # Sequence 201 S TQNFSSESK 0.322 p38MAPK . | | | |  |  |
| # Sequence 201 S TQNFSSESK 0.288 PKC . | | | |  |  |
| # Sequence 201 S TQNFSSESK 0.266 PKG . | | | |  |  |
| # Sequence 201 S TQNFSSESK 0.250 RSK . | | | |  |  |
| # Sequence 201 S TQNFSSESK 0.182 cdk5 . | | | |  |  |
| # Sequence 201 S TQNFSSESK 0.083 PKA . | | | |  |  |
| # Sequence 201 S TQNFSSESK 0.077 PKB . | | | |  |  |
| # |  |  |  |  |  |
| # Sequence 202 S QNFSSESKR 0.766 unsp YES | | | |  |  |
| # Sequence 202 S QNFSSESKR 0.516 CKII YES | | | |  |  |
| # Sequence 202 S QNFSSESKR 0.510 cdc2 YES | | | |  |  |
| # Sequence 202 S QNFSSESKR 0.456 CKI . | | | |  |  |
| # Sequence 202 S QNFSSESKR 0.432 GSK3 . | | | |  |  |
| # Sequence 202 S QNFSSESKR 0.430 CaM-II . | | | |  |  |
| # Sequence 202 S QNFSSESKR 0.393 RSK . | | | |  |  |
| # Sequence 202 S QNFSSESKR 0.359 DNAPK . | | | |  |  |
| # Sequence 202 S QNFSSESKR 0.272 PKC . | | | |  |  |
| # Sequence 202 S QNFSSESKR 0.269 ATM . | | | |  |  |
| # Sequence 202 S QNFSSESKR 0.262 PKG . | | | |  |  |
| # Sequence 202 S QNFSSESKR 0.249 p38MAPK . | | | |  |  |
| # Sequence 202 S QNFSSESKR 0.205 cdk5 . | | | |  |  |
| # Sequence 202 S QNFSSESKR 0.156 PKA . | | | |  |  |
| # Sequence 202 S QNFSSESKR 0.079 PKB . | | | |  |  |
| # |  |  |  |  |  |
| # Sequence 204 S FSSESKRET 0.996 unsp YES | | | |  |  |
| # Sequence 204 S FSSESKRET 0.614 PKC YES | | | |  |  |
| # Sequence 204 S FSSESKRET 0.586 CKII YES | | | |  |  |
| # Sequence 204 S FSSESKRET 0.439 cdc2 . | | | |  |  |
| # Sequence 204 S FSSESKRET 0.437 CaM-II . | | | |  |  |
| # Sequence 204 S FSSESKRET 0.430 GSK3 . | | | |  |  |
| # Sequence 204 S FSSESKRET 0.422 CKI . | | | |  |  |
| # Sequence 204 S FSSESKRET 0.350 DNAPK . | | | |  |  |
| # Sequence 204 S FSSESKRET 0.311 p38MAPK . | | | |  |  |
| # Sequence 204 S FSSESKRET 0.288 ATM . | | | |  |  |
| # Sequence 204 S FSSESKRET 0.263 RSK . | | | |  |  |
| # Sequence 204 S FSSESKRET 0.244 PKG . | | | |  |  |
| # Sequence 204 S FSSESKRET 0.141 cdk5 . | | | |  |  |
| # Sequence 204 S FSSESKRET 0.119 PKA . | | | |  |  |
| # Sequence 204 S FSSESKRET 0.083 PKB . | | | |  |  |
| # |  |  |  |  |  |
| # Sequence 231 S LNVLSPRGV 0.576 p38MAPK YES | | | |  |  |
| # Sequence 231 S LNVLSPRGV 0.510 GSK3 YES | | | |  |  |
| # Sequence 231 S LNVLSPRGV 0.452 cdk5 . | | | |  |  |
| # Sequence 231 S LNVLSPRGV 0.446 CaM-II . | | | |  |  |
| # Sequence 231 S LNVLSPRGV 0.404 unsp . | | | |  |  |
| # Sequence 231 S LNVLSPRGV 0.386 CKI . | | | |  |  |
| # Sequence 231 S LNVLSPRGV 0.348 DNAPK . | | | |  |  |
| # Sequence 231 S LNVLSPRGV 0.328 cdc2 . | | | |  |  |
| # Sequence 231 S LNVLSPRGV 0.315 ATM . | | | |  |  |
| # Sequence 231 S LNVLSPRGV 0.274 RSK . | | | |  |  |
| # Sequence 231 S LNVLSPRGV 0.267 CKII . | | | |  |  |
| # Sequence 231 S LNVLSPRGV 0.228 PKG . | | | |  |  |
| # Sequence 231 S LNVLSPRGV 0.116 PKA . | | | |  |  |
| # Sequence 231 S LNVLSPRGV 0.094 PKC . | | | |  |  |
| # Sequence 231 S LNVLSPRGV 0.077 PKB . | | | |  |  |
| # |  |  |  |  |  |
| # Sequence 254 S QCRPSKGRK 0.996 unsp YES | | | |  |  |
| # Sequence 254 S QCRPSKGRK 0.804 PKC YES | | | |  |  |
| # Sequence 254 S QCRPSKGRK 0.470 GSK3 . | | | |  |  |
| # Sequence 254 S QCRPSKGRK 0.443 PKG . | | | |  |  |
| # Sequence 254 S QCRPSKGRK 0.422 CaM-II . | | | |  |  |
| # Sequence 254 S QCRPSKGRK 0.374 CKI . | | | |  |  |
| # Sequence 254 S QCRPSKGRK 0.360 RSK . | | | |  |  |
| # Sequence 254 S QCRPSKGRK 0.358 cdc2 . | | | |  |  |
| # Sequence 254 S QCRPSKGRK 0.356 DNAPK . | | | |  |  |
| # Sequence 254 S QCRPSKGRK 0.337 PKA . | | | |  |  |
| # Sequence 254 S QCRPSKGRK 0.325 p38MAPK . | | | |  |  |
| # Sequence 254 S QCRPSKGRK 0.284 ATM . | | | |  |  |
| # Sequence 254 S QCRPSKGRK 0.212 cdk5 . | | | |  |  |
| # Sequence 254 S QCRPSKGRK 0.211 CKII . | | | |  |  |
| # Sequence 254 S QCRPSKGRK 0.101 PKB . | | | |  |  |
| # |  |  |  |  |  |
| # Sequence 287 S VHCYSMQSK 0.463 GSK3 . | | | |  |  |
| # Sequence 287 S VHCYSMQSK 0.432 CaM-II . | | | |  |  |
| # Sequence 287 S VHCYSMQSK 0.408 cdc2 . | | | |  |  |
| # Sequence 287 S VHCYSMQSK 0.379 CKII . | | | |  |  |
| # Sequence 287 S VHCYSMQSK 0.374 DNAPK . | | | |  |  |
| # Sequence 287 S VHCYSMQSK 0.373 CKI . | | | |  |  |
| # Sequence 287 S VHCYSMQSK 0.340 p38MAPK . | | | |  |  |
| # Sequence 287 S VHCYSMQSK 0.314 ATM . | | | |  |  |
| # Sequence 287 S VHCYSMQSK 0.309 RSK . | | | |  |  |
| # Sequence 287 S VHCYSMQSK 0.303 PKC . | | | |  |  |
| # Sequence 287 S VHCYSMQSK 0.257 PKG . | | | |  |  |
| # Sequence 287 S VHCYSMQSK 0.201 cdk5 . | | | |  |  |
| # Sequence 287 S VHCYSMQSK 0.199 PKA . | | | |  |  |
| # Sequence 287 S VHCYSMQSK 0.097 PKB . | | | |  |  |
| # Sequence 287 S VHCYSMQSK 0.012 unsp . | | | |  |  |
| # |  |  |  |  |  |
| # Sequence 290 S YSMQSK--- 0.586 PKC YES | | | |  |  |
| # Sequence 290 S YSMQSK--- 0.442 CaM-II . | | | |  |  |
| # Sequence 290 S YSMQSK--- 0.441 GSK3 . | | | |  |  |
| # Sequence 290 S YSMQSK--- 0.423 cdc2 . | | | |  |  |
| # Sequence 290 S YSMQSK--- 0.371 CKI . | | | |  |  |
| # Sequence 290 S YSMQSK--- 0.371 CKII . | | | |  |  |
| # Sequence 290 S YSMQSK--- 0.346 DNAPK . | | | |  |  |
| # Sequence 290 S YSMQSK--- 0.341 RSK . | | | |  |  |
| # Sequence 290 S YSMQSK--- 0.321 ATM . | | | |  |  |
| # Sequence 290 S YSMQSK--- 0.316 p38MAPK . | | | |  |  |
| # Sequence 290 S YSMQSK--- 0.261 PKG . | | | |  |  |
| # Sequence 290 S YSMQSK--- 0.256 PKA . | | | |  |  |
| # Sequence 290 S YSMQSK--- 0.181 cdk5 . | | | |  |  |
| # Sequence 290 S YSMQSK--- 0.089 PKB . | | | |  |  |
| # Sequence 290 S YSMQSK--- 0.013 unsp . | | | |  |  |
