# Supplementary material for: EGFR-dependent delivery of phosphorylated IGFBP3 by cancer stem cells drives chemotherapy resistance in colorectal cancer
Source: Cell Death Dis. 2026 Jun 29;17(1):804. doi: 10.1038/s41419-026-09009-0 (PMC13578265; doi:10.1038/s41419-026-09009-0)
Supplement: Supplementary file 4 [file 41419_2026_9009_MOESM4_ESM.docx]

Supplementary Table 3. Members of the CK1 kinase family responsible for phosphorylation at the S201 site of IGFBP3 predicted by the iGPS software

| **ID** | **Position** | **Code** | **Peptide** | **Matched ID** | **Gene Name** | **Kinase ID** | **Kinase Name** | **Interaction** | **Predictor** | **Score** | **Cutoff** |
| --- | --- | --- | --- | --- | --- | --- | --- | --- | --- | --- | --- |
| NP_000589.2 | 201 | S | STDTQNFSSESKRET | P17936 | IGFBP3 | P49674 | CK1e | String | CK1 | 2.68 | 2.15 |
| NP_000589.2 | 201 | S | STDTQNFSSESKRET | P17936 | IGFBP3 | Q5TCY1 | TTBK1 | String | CK1 | 2.68 | 2.15 |
| NP_000589.2 | 201 | S | STDTQNFSSESKRET | P17936 | IGFBP3 | Q8IV63 | VRK3 | String | CK1 | 2.68 | 2.15 |
| NP_000589.2 | 201 | S | STDTQNFSSESKRET | P17936 | IGFBP3 | Q9HCP0 | CK1g1 | String | CK1 | 2.68 | 2.15 |
| NP_000589.2 | 201 | S | STDTQNFSSESKRET | P17936 | IGFBP3 | Q6IQ55 | TTBK2 | String | CK1 | 2.68 | 2.15 |
| NP_000589.2 | 201 | S | STDTQNFSSESKRET | P17936 | IGFBP3 | Q86Y07 | VRK2 | String | CK1 | 2.68 | 2.15 |
| NP_000589.2 | 201 | S | STDTQNFSSESKRET | P17936 | IGFBP3 | P48730 | CK1d | String | CK1 | 2.68 | 2.15 |
| NP_000589.2 | 201 | S | STDTQNFSSESKRET | P17936 | IGFBP3 | P78368 | CK1g2 | String | CK1 | 2.68 | 2.15 |
